# The antibody landscapes following AS03 and MF59 adjuvanted H5N1 vaccination

**DOI:** 10.1038/s41541-022-00524-7

**Published:** 2022-08-30

**Authors:** Johannes B. Goll, Aarti Jain, Travis L. Jensen, Rafael Assis, Rie Nakajima, Algis Jasinskas, Lynda Coughlan, Sami R. Cherikh, Casey E. Gelber, S. Khan, D. Huw Davies, Philip Meade, Daniel Stadlbauer, Shirin Strohmeier, Florian Krammer, Wilbur H. Chen, Philip L. Felgner

**Affiliations:** 1 The Emmes Company, LLC, Rockville, MD USA; 2 Vaccine R&D Center, Department of Physiology and Biophysics, University of California-Irvine, Irvine, CA USA; 3 Center for Vaccine Development and Global Health, University of Maryland School of Medicine, Baltimore, MD USA; 4 Department of Microbiology and Immunology, University of Maryland School of Medicine, Baltimore, MD USA; 5 Department of Microbiology, Icahn School of Medicine at Mount. Sinai, New York City, NY USA; 6 Present Address: Moderna Inc., Cambridge, MA USA

**Keywords:** Influenza virus, Randomized controlled trials

## Abstract

Current seasonal and pre-pandemic influenza vaccines induce short-lived predominantly strain-specific and limited heterosubtypic responses. To better understand how vaccine adjuvants AS03 and MF59 may provide improved antibody responses to vaccination, we interrogated serum from subjects who received 2 doses of inactivated monovalent influenza A/Indonesia/05/2005 vaccine with or without AS03 or MF59 using hemagglutinin (HA) microarrays (NCT01317758 and NCT01317745). The arrays were designed to reflect both full-length and globular head HA derived from 17 influenza A subtypes (H1 to H16 and H18) and influenza B strains. We observed significantly increased strain-specific and broad homo- and heterosubtypic antibody responses with both AS03 and MF59 adjuvanted vaccination with AS03 achieving a higher titer and breadth of IgG responses relative to MF59. The adjuvanted vaccine was also associated with the elicitation of stalk-directed antibody. We established good correlation of the array antibody responses to H5 antigens with standard HA inhibition and microneutralization titers.

## Introduction

Avian influenza viruses represent a continuous pandemic threat, as illustrated by the frequent emergence of novel reassortant viruses which have resulted in sporadic spillover events from the zoonotic reservoir in the past few decades^1–3^. These antigenically distinct influenza viruses are categorized into subtypes (i.e., H5, H7) based on phylogenetic characterization and sequence homology of the hemagglutinin (HA) gene, and are further sub-divided into clades. A key component of the US pandemic preparedness has been ongoing surveillance of prominent influenza viral clades, which may lead to their selection for development into vaccines that can be added to the National Pre-Pandemic Influenza Vaccine Stockpile (NPIVS)^4^. The rationale behind the stockpile is that a vaccine from pre-pandemic subtype viruses can provide partial cross-protection^5^, thereby benefiting vaccinated priority groups before a better-matched vaccine against the pandemic strain becomes available. The NPIVS program currently contains multiple pre-pandemic influenza antigens, representing various H5Nx and H7N9 avian influenza viruses.

Inactivated subvirion influenza vaccines against avian HA strains have demonstrated poor immunogenicity in unprimed populations^6^. Therefore, the NPIVS program also maintains two immune-stimulating adjuvants (AS03^7^ and MF59^8^). These adjuvants are intended to be deployed with a respective vaccine antigen in a mix and match strategy to provide better immune responses to vaccination, which might translate to dose-sparing of the limited supply and faster onset, greater breadth, and/or longer duration of protection with vaccination^9–11^. One of the present gaps in knowledge is an understanding of the effect of these adjuvants on the breadth of the antibody responses elicited by pre-pandemic vaccines.

Influenza HA is a major target for humoral immune responses, with antibodies directed towards the antigenically variable head domain (HA1) and the highly conserved HA stalk domain (HA2)^12,13^. In recent years, it has been determined that non-neutralizing, HA stalk-specific antibodies, which display the breadth of reactivity by ELISA, can confer protection from heterosubtypic influenza virus challenges in animal models^14–16^. Furthermore, such broadly reactive antibodies have recently been proposed as a correlate of protection in human cohort studies^17^ of natural influenza virus infection. Therefore, there is growing interest in identifying adjuvants that are capable of inducing such broadly reactive stalk-specific immune responses with the potential to confer breadth of protection against diverse influenza viruses^18^.

One novel method for more comprehensively assessing the breadth of antibody responses is protein microarrays^19–21^. We constructed two sets of influenza-specific high-density protein microarrays which comprised purified HA proteins derived from 17 influenza A virus subtypes and influenza B virus strains, including conformationally correct stabilize trimers. Our present study was designed to measure the landscape of the antibody responses in response to vaccination with an inactivated, monovalent, subvirion influenza A/Indonesia/05/2005 (H5N1) strain vaccine when administered alone (unadjuvanted) or with AS03 or MF59 adjuvant. Importantly, using stabilized trimeric headless stalk protein (HA2) in competitive inhibition assays, we indirectly assessed the elicitation of stalk-directed antibodies. Finally, the concordance between microarray subtype-specific antibody levels and HAI and MN titers were evaluated.

## Results

### Pre-vaccination reactivity against influenzas viruses and the impact of prior seasonal influenza vaccination

**Fig. 1:**
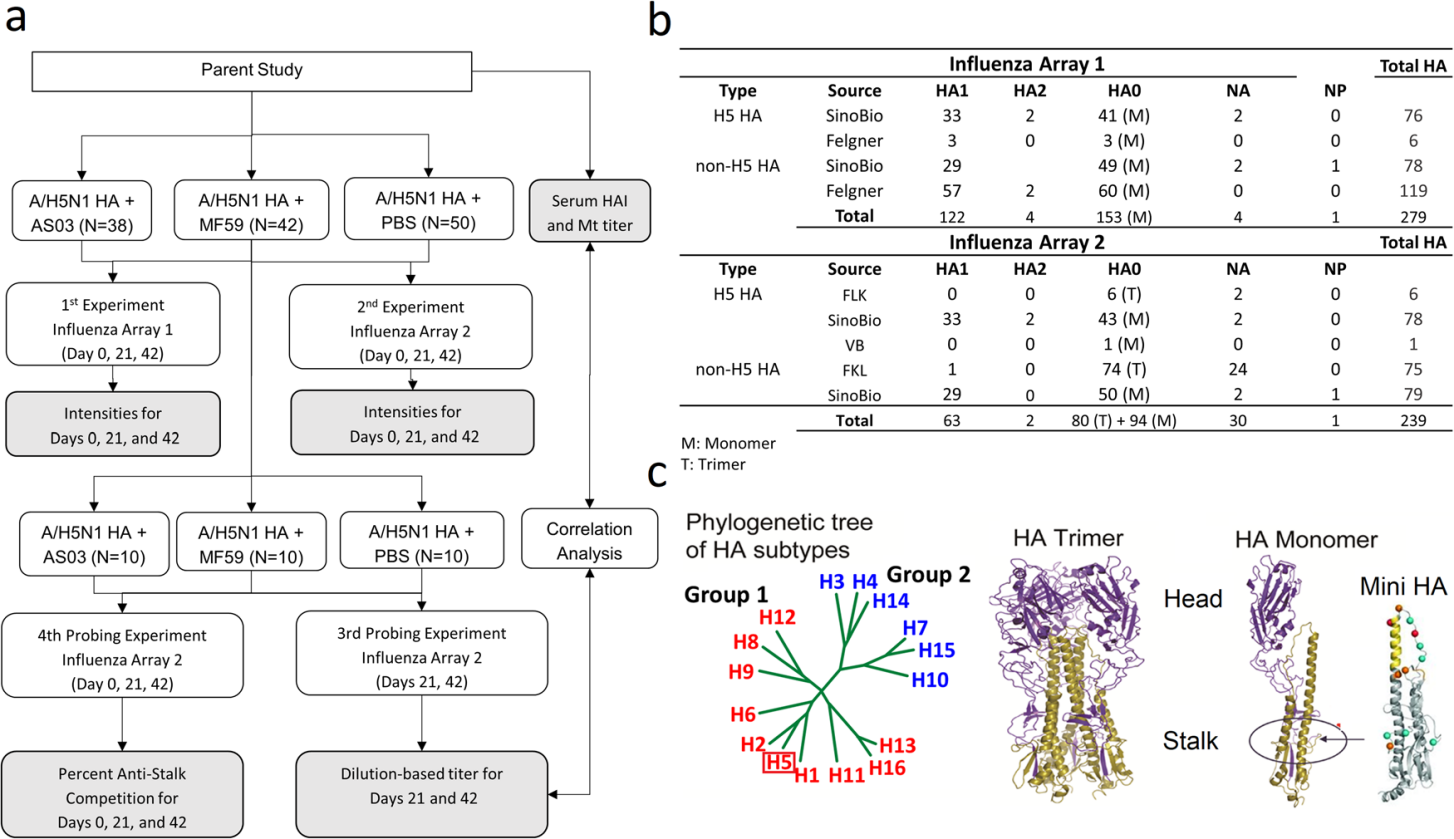
Study overview. **a** Study design. **b** Protein array antigen composition. **c** HA Subtype relationship^51^, H5 trimer structure, Mini-HA binding used for assessing percent anti-stalk competition^49^.

We interrogated 390 serum specimens from 130 clinical trial volunteers who received two doses administered 21 days apart of inactivated influenza A/Indonesia/05/2005 (H5N1 clade 2.2.3) virus vaccine containing 15 µg of HA using two different protein microarrays. The vaccines were either unadjuvanted or included AS03^11^ or MF59^22^ adjuvants. Age ranged from 19 to 47 years with a median age of 27-28.5 years (Supplementary Table 1). Figure 1 summarizes our experimental design and array composition. Array #2 included 80 stabilized HA trimers not present on Array #1. Our results showed that these full-length stabilized trimeric HA0 molecules detected more antibodies than monomeric HA0 (Supplementary Fig. 1). We attributed this difference to the presence of additional conformational epitopes present in the stabilized trimers resulting in increased reactivity.

**Fig. 2:**
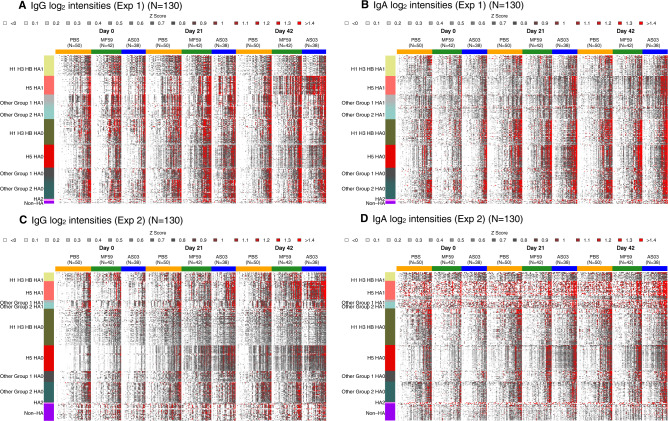
Heatmaps summarizing experiments 1 & 2 protein array IgG and IgA results by vaccine group, day, HA molecule, and HA subtype. **A**, **B** log_2_ fluorescent intensity results by HA subtype for Experiment 1 IgG and IgA antibodies, respectively. **C**, **D** Corresponding results for Experiment 2. Each column represents one sample from a subject, and each row represents an antigen. The antigens are organized into groups, headgroup (HA1) or full-length molecule (HA0). The HA subtypes were grouped as H1/H3/FluB, H5, Other Group 1, and Other Group 2. Fluorescent intensities were background corrected, log_2_ transformed, and standardized across rows (*z* score, mean = 0, variance = 1). In red: intensities strongly increased compared to the mean log_2_ intensity, in dark gray: moderately increased compared to the mean in white decreased relative to the mean. Samples were ordered by increasing reactivity.

A birds’ eye overview of all IgG and IgA results for both arrays and antibody isotypes (IgG and IgA) is shown in Fig. 2. The heatmaps show pre-vaccination (day 0) reactivity patterns for each of the 3 vaccine groups were similar. For both Array #1 and Array #2, pre-vaccination IgG and IgA antibodies were highest against seasonal influenza virus antigens (H1, H3, or FluB) HA0 and HA1 (Supplementary Fig. 2), with higher responses against HA0 than HA1. While H5 antibodies ranked in the lower third for HA1 responses, their responses ranked slightly lower than seasonal virus antigens based on HA0 responses. We inferred that these reactivities against H5 HA0 as caused by cross-reactive antibodies were originally induced by seasonal H1 exposure, as these were presumed H5-naive subjects with no overt reason for prior exposure to H5 influenza. This prompted us to explore the impact of prior seasonal influenza vaccination on the microarray antibody responses. We found that subjects reporting seasonal influenza vaccination within the prior 2 years had lower fold changes in antibody intensities against vaccine subtype antigens (H5 HAs) relative to pre-vaccination irrespective of experiment, vaccine group, antibody type, or timepoint (Supplementary Fig. 3). Sex and age did not have a large impact.

### Adjuvant-mediated induction of antibodies against the HA1 and HA0 molecule

**Fig. 3:**
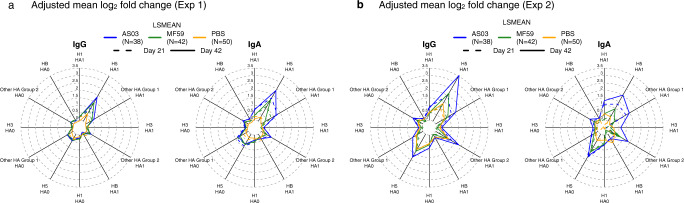
Adjuvants boosted IgG and IgA antibodies against head H5 HA proteins post the second dose. **a** Experiment 1 IgG and IgA radar plots summarizing the mean log_2_ fold change relative to pre-vaccination adjusted for prior seasonal influenza vaccination. **b** Corresponding Experiment 2 results. For each antigen, first, the mean log_2_ fold change in antibody fluorescent intensity relative to pre-vaccination adjusted for prior seasonal influenza vaccination was determined (LSMEAN). The value for each ray in the radar plot was then calculated as the mean of the LSMEANs for the respective antigen group, vaccine group, and timepoint combination.

Next, we analyzed vaccine responses after adjusting for pre-vaccination levels and prior seasonal influenza vaccination. The mean log_2_ fold change relative to pre-vaccination adjusted for seasonal influenza vaccination is summarized in Fig. 3. The analysis compares the subtype-specific change in antibody levels to HA1 and HA0 induced by the three vaccine groups. Except for HB HA1, compared to adjuvanted vaccines, the unadjuvanted vaccine induced smaller log_2_ fold changes relative to pre-vaccination against HA1 among all the HA subtypes at both day 21 and day 42. The adjuvanted vaccines induced a substantial increase in IgG and IgA against H5 HA1 after the first dose and there was a noticeable further increase after the second dose. These adjuvanted vaccine responses against H5 HA1 were greater for AS03 than MF59 (Fig. 3). This average H5 HA1 boosting effect was observed for the majority of individual H5 HA1 antigens (Supplementary Fig. 4).

However, the response patterns against H5 HA0 were less strong, in particular for Array #1 for Exp 1 (Fig. 3a). We attributed the more profound responses to HA1 compared to HA0 to cross-reactivity of antibody against the H5 stalk at pre-vaccination, which could have been elicited from prior seasonal group 1 vaccination or infection diminishing the fold change effect for H5 HA0. In addition, the day 21 IgG and IgA response for Array #2 used for Exp 2 against H5 HA0 for the adjuvanted groups was more pronounced than for Array #1 used for Exp 1 (Fig. 3b). This increase in H5 HA0 reactivity was also seen for the unadjuvanted vaccine group for IgG but not IgA. We attributed this difference between arrays to the presence of additional conformational epitopes present in 80 stabilized trimers resulting in increased average reactivity (Supplementary Fig. 1). Furthermore, a heterosubtypic cross-reactive response against group 1 HA1 and other group 2 HA1 was evident, in particular in the AS03-adjuvanted vaccine group, that was not much boosted with a second dose, which we interpreted as indicating heterosubtypic cross-reactivity against the conserved stalk (Fig. 3b). It is possible the heterosubtypic group 1 HA1 responses were to the N-terminus of HA1, which contains a conserved 115 amino acid sequence that forms part of the cross-reactive stalk (Fig. 1c).

### Adjuvant effect on heterosubtypic responses

**Fig. 4:**
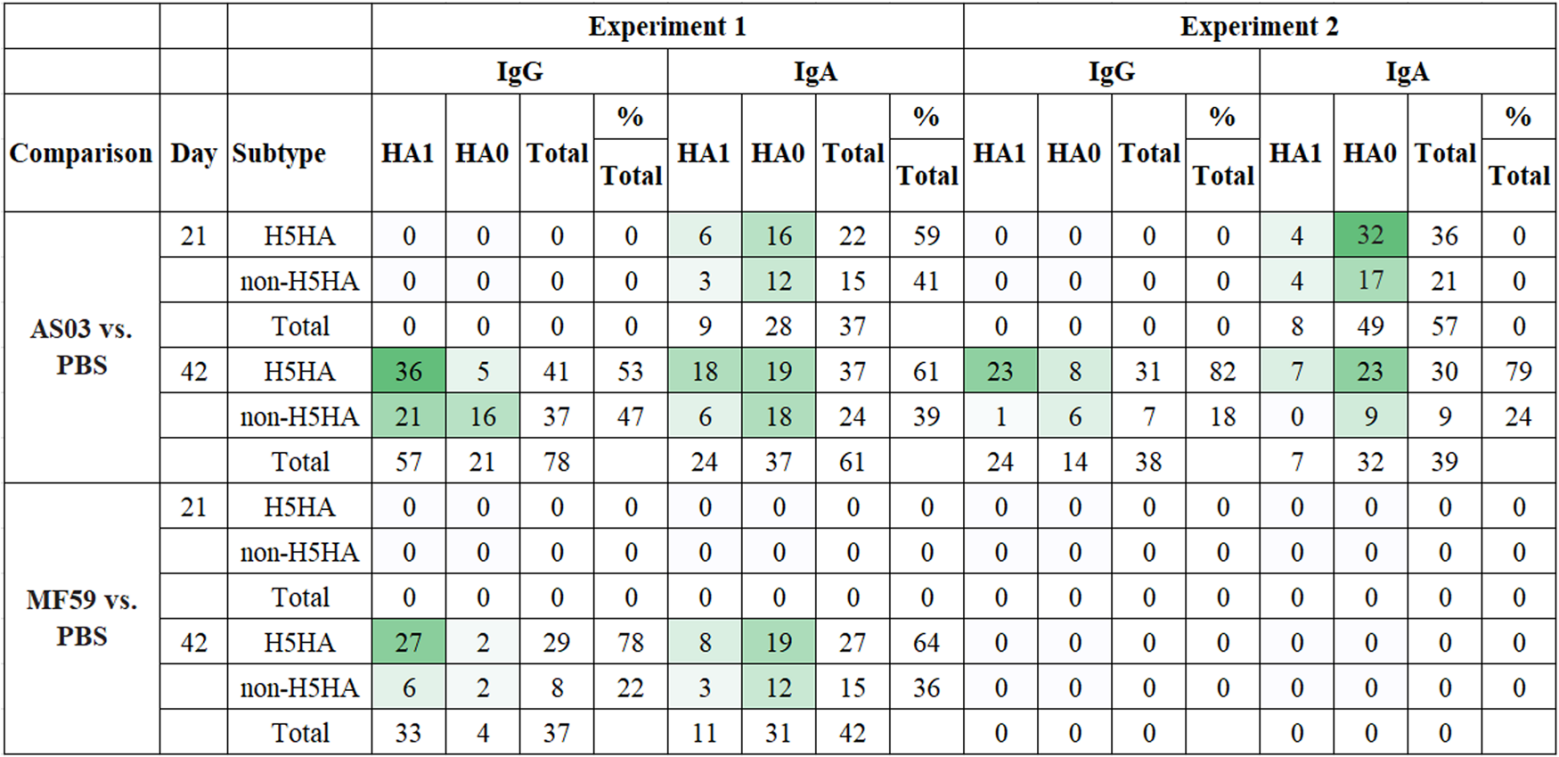
Summary of statistically significant adjuvant-boosted antibody responses. For each antigen, an ANOVA model was fitted to day 21 and day 42 log_2_ fold change responses to assess the statistical significance of contrasts that compared adjuvanted vs. control group responses (AS03–PBS and MF59–PBS) adjusting for prior seasonal influenza vaccination using an FDR-adjusted *p* value <0.1.

To statistically assess the impact of the adjuvants on heterosubtypic breadth of coverage, we fit ANOVA models for days 21 and 42 comparing adjuvanted vs. unadjuvanted responses adjusting for pre-vaccination and prior seasonal influenza vaccination (Fig. 4). For Array #1 Exp 1, at day 42 (21 days post second dose), AS03-adjuvanted vaccine elicited over twice as many significant IgG responses against HA antigens and broader coverage of significant responses against non-H5 HAs compared to MF59. This was demonstrated by a significant increase in IgG responses against 78 unique HA antigens in the AS03 group (47% non-H5 HA antigens) and 37 unique HA antigens in the MF59 group (22% non-H5 HA antigens). This difference in the proportion of recognition of non-H5 HA antigens was statistically significant (two-sided Fisher’s exact test <0.01) with the AS03 group eliciting more responses against non-H5 HA0 antigens (Fig. 4). All 37 unique HA antigens identified for the MF59 group were a complete subset of the 78 unique HA antigens in the AS03 group. For both adjuvanted groups, the most frequent responses relative to the unadjuvanted group were IgG responses against HA1 antigens (AS03: 73%, MF59: 89%), and these were predominantly among H5 HA1 antigens (AS03: 63%, MF59: 82%). When assessing IgA responses in Array #1 Exp 1, the AS03 group showed statistically significant responses against 28 unique antigens on day 21 and 37 antigens on day 42. Most of these AS03 responses (76%) were against HA0 at day 21, of which 43% were against non-H5 HA0; at day 42, 61% of the unique antigens were against HA0, of which 49% were against non-H5 HA0. A substantial additional number of H5 HA1 antigens were recognized by IgA at day 42 (30%) relative to day 21 (16%). In contrast to IgG, there were more IgA responses against HA0 with AS03 at day 42 (two-sided Fisher’s exact test <0.001).

For the MF59 group, 42 statistically significant IgA responses were identified, of which 31 (74%) were against HA0 and from which 39% were against non-H5 HA0. In contrast to IgG, the difference in H5 vs. non-H5 HA antigen recognition on day 42 between the AS03 and MF59 group was not statistically significant. In Array #2 Exp 2, only the AS03 adjuvant was associated with statistically significant responses that differed compared to the unadjuvanted group (Fig. 4). This included IgG against 38 antigens, of which 31 (82%) were H5 HA and 7 (18%) were non-H5 HA antigens. While most H5 HA IgG responses were observed against HA1 (74%), responses against non-H5 HA were primarily observed against HA0 (86%). In addition, the AS03 group elicited statistically significant IgA response against 57 antigens on day 21 and 39 antigens on day 42. The majority of these responses were against HA0 antigens, of which 35% were against non-H5 HA0 on day 21 and 28% on day 42.

### Adjuvant effect on the antibody landscape of group 1 and 2 influenza virus antigens

**Fig. 5:**
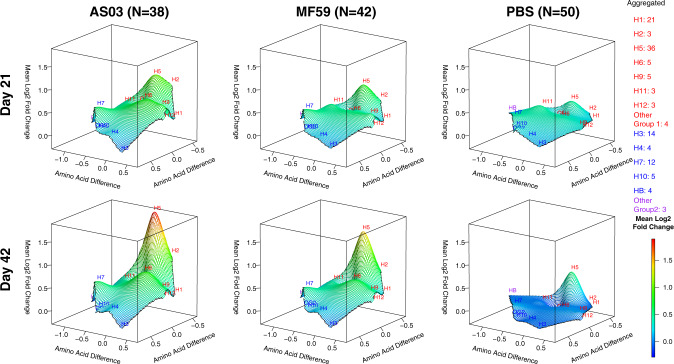
Adjuvants boosted IgG antibodies against head H5 HA proteins post second dose. IgG antibody landscapes for HA1 subunit antigens based on mean log_2_ fold change relative to pre-vaccination adjusted for seasonal influenza vaccination (Experiment 1). In red: Group 1 HA1 antigens, in blue: Group 2 HA1 antigens, in purple: influenza B HA1. The *y* and *x* axes project antigen protein sequence divergence as measured in the number of amino acid differences between antigen protein using multidimensional scaling. For each antigen, first the mean log_2_ fold change in antibody fluorescent intensity relative to pre-vaccination adjusted for prior seasonal influenza vaccination was determined (LSMEAN). For subtypes with multiple antigens, the mean LSMEAN and MDS dimensions were used to present the results in the 3D space.

**Fig. 6:**
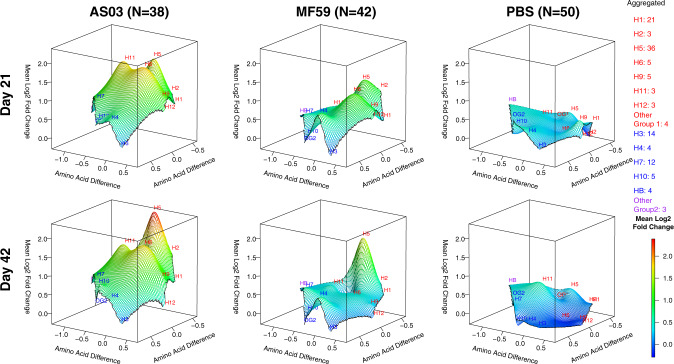
Adjuvants boosted IgA antibodies against head H5 HA proteins post second dose. IgA antibody landscapes for HA1 subunit antigens based on mean log_2_ fold change relative to pre-vaccination adjusted for seasonal influenza vaccination (Experiment 1). In red: Group 1 HA1 antigens, in blue: Group 2 HA1 antigens, in purple: influenza B HA1. The *y* and *x* axes project antigen protein sequence divergence as measured in the number of amino acid differences between antigen protein using multidimensional scaling. For each antigen, first the mean log_2_ fold change in antibody fluorescent intensity relative to pre-vaccination adjusted for prior seasonal influenza vaccination was determined (LSMEAN). For subtypes with multiple antigens, the mean LSMEAN and MDS dimensions were used to present the results in the 3D space.

**Fig. 7:**
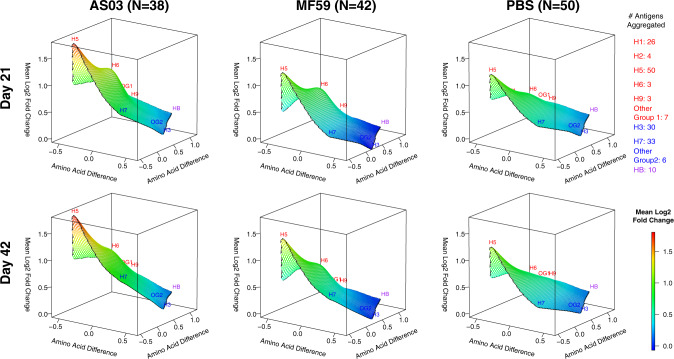
All three vaccines elicited increased IgG antibodies against full-length H5 proteins after the first dose without further boosting after the second dose. IgG antibody landscapes for HA0 antigens based on mean log_2_ fold change relative to pre-vaccination adjusted for seasonal influenza vaccination (Experiment 2). In red: Group 1 HA0 antigens, in blue: Group 2 HA0 antigens, in purple: influenza B HA0. The *y* and *x* axes project antigen protein sequence divergence as measured in the number of amino acid differences between antigen proteins using multidimensional scaling. For each antigen, first the mean log_2_ fold change in antibody fluorescent intensity relative to pre-vaccination adjusted for prior seasonal influenza vaccination was determined (LSMEAN). For subtypes with multiple antigens, the mean LSMEAN and MDS dimensions were used to present the results in the 3D space.

**Fig. 8:**
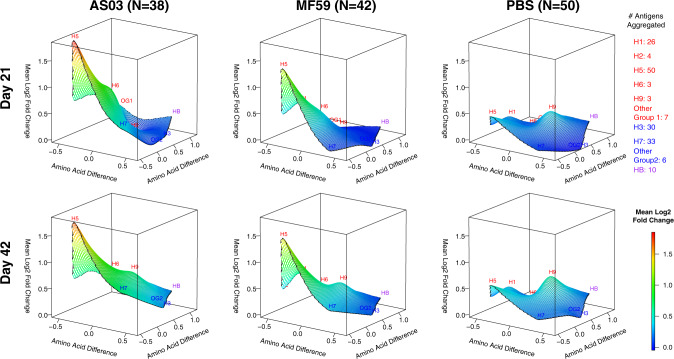
Only adjuvanted vaccines elicited increased IgA antibodies against full-length H5 proteins after the first dose. IgA antibody landscapes for HA0 antigens based on mean log_2_ fold change relative to pre-vaccination adjusted for seasonal influenza vaccination (Experiment 2). In red: Group 1 HA0 antigens, in blue: Group 2 HA0 antigens, in purple: influenza B HA0. The *y* and *x* axes project antigen protein sequence divergence as measured in the number of amino acid differences between antigen proteins using multidimensional scaling. For each antigen, first, the mean log_2_ fold change in antibody fluorescent intensity relative to pre-vaccination adjusted for prior seasonal influenza vaccination was determined (LSMEAN). For subtypes with multiple antigens, the mean LSMEAN and MDS dimensions were used to present the results in the 3D space.

Next, to further assess heterosubtypic breath, we generated antibody landscapes that profile the antibody response against influenza virus antigens as a function of antigenic differences on the sequence level (Figs. 5–8). The landscapes contrast homosubtypic and heterosubtypic breadth and intensity of the IgG and IgA response against HA variants elicited by the three vaccine groups separately, against either the HA1 (Exp 1: Figs. 5, 6) or the HA0 molecules (Exp 2: Figs. 7, 8) and were adjusted for prior-influenza vaccination. On day 21, AS03-adjuvanted vaccine induced homosubtypic responses against H5 HA1 variants and heterosubtypic cross-reactive responses against closely related group 1 antigens that increased on day 42 (Figs. 5, 6). A similar pattern was seen with MF59, but the effect was less pronounced than for AS03. In contrast, the response with unadjuvanted vaccine, while still showing a distinct homosubtypic response against H5 HAs, was much weaker. Antibody landscapes for IgG against HA0 are shown in Fig. 7. In the adjuvanted vaccine groups, the IgG responses after a second dose (day 42) with HA1 (Fig. 5) were not seen against HA0 (Fig. 7). However, all three vaccine groups induced increased IgG against H5 HA0 proteins with similar intensities after one and two doses (Fig. 7). For IgA, in contrast to IgG, the higher response to a second dose was only seen in the adjuvanted groups and was absent from the unadjuvanted group (Fig. 8).

### Antibody directed against HA stalk

In order to estimate the relative levels of anti-stalk responses, we performed anti-stalk experiments for a subset of sera from 30 subjects (10 per vaccine group) (Fig. 1a, c). Trends of anti-stalk blocking effects by mini H1 HA concentration are provided in Supplementary Fig. 5. The pre-incubation of serum specimens with mini-HA reduced up to 67% of the IgG and 57% of the IgA antibody binding responses against H5 detected on Array #2 relative to the negative control when using the intensity area under the curve for both concentrations (Fig. 10). We attributed this reduction in detected binding responses to competitive binding of the mini-HA to anti-stalk-specific antibody in the sample. At baseline (day 0), about 45% of the IgG responses and 30% of IgA responses against H5 were blocked by the mini-HA stalk. H5 immunization increased the percentage of anti-stalk antibody that was blocked by mini-HA. Baseline (pre-vaccination) specimens demonstrated a ~25–30% diminishment of the H1 HA0 IgG and IgA signal with mini-HA pre-treatment. After vaccination (days 21 and 42), the percentage of H1 HA0 IgG and IgA blocked by the mini-HA increased, consistent with an increase in vaccine-induced heterosubtypic cross-reactive antibody responses against H1.

**Fig. 9:**
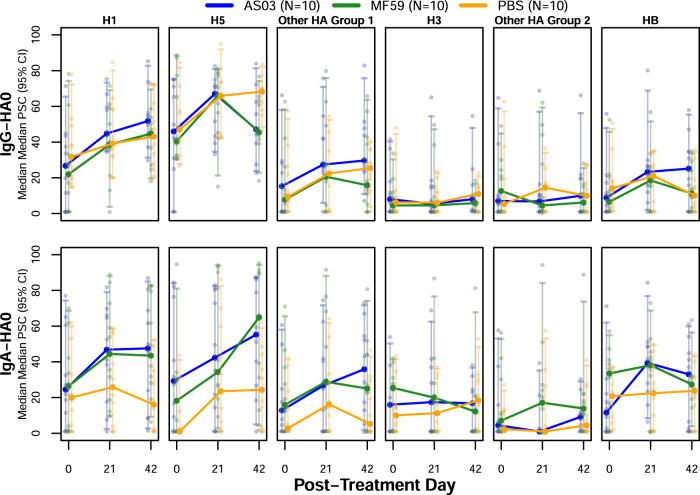
Median average percent anti-stalk blocking results for IgG and IgA antibodies against full-length HA antigens over time. Percent stalk competition (PSC) for each sample and antigen was calculated as: 100−(AUC with competition (mini-HA added)/AUC without competition (RSV-F negative control added) × 100) where the AUC represented the area under the curve for fluorescence intensity for two different concentrations of mini-HA (10 µg and 50 µg/mL). For each subject, PSC values were then aggregated across antigens for the respective antigen group using the mean. For each vaccine group (*n* = 10 each), the median AUC was determined. The error bars represent the 95% bootstrap confidence interval of the median. Dots represent individual average PSC results for each subject and time point.

At day 21 (post-first dose), all three vaccine groups demonstrated the highest increase of stalk-directed responses relative to pre-vaccination. These purported stalk responses were primarily cross-reactive against H1 and H5 but also to a lesser extent other group 1 HA and HB antigens. Except for the IgG responses against H5, day 42 (post second dose) results for H1 and H5 demonstrated a further increase in percent anti-stalk responses, with the strongest increases observed for percent stalk-directed IgA responses against H5. For the adjuvanted groups, the day 42 IgG percent stalk antibody response was lower than that at day 21 while it slightly increased for the PBS group. For both adjuvanted groups, the decrease in percent anti-stalk IgG relative to the PBS group was statistically significant (AS03 and MF59 median=48%, PBS median=65%; two-sided Wilcoxon Rank Sum Test *P* < 0.05). We hypothesize this was the result of the majority of the antibody response being directed against the immunodominant headgroup of H5 subsequent to the second dose of vaccine. Interestingly, at day 42, after two doses of H5N1 vaccine, AS03-adjuvanted H5 vaccination showed an increase in percent anti-stalk IgG antibody against other HA groups 1 as well as HB HA0 antigens while MF59 responses decreased with an absolute median difference between adjuvant groups of ≥14% anti-stalk IgG antibody against HB (Fig. 9).

### Correlation of microneutralization and hemagglutination inhibition titers with microarray dilution-based titers

**Fig. 10:**
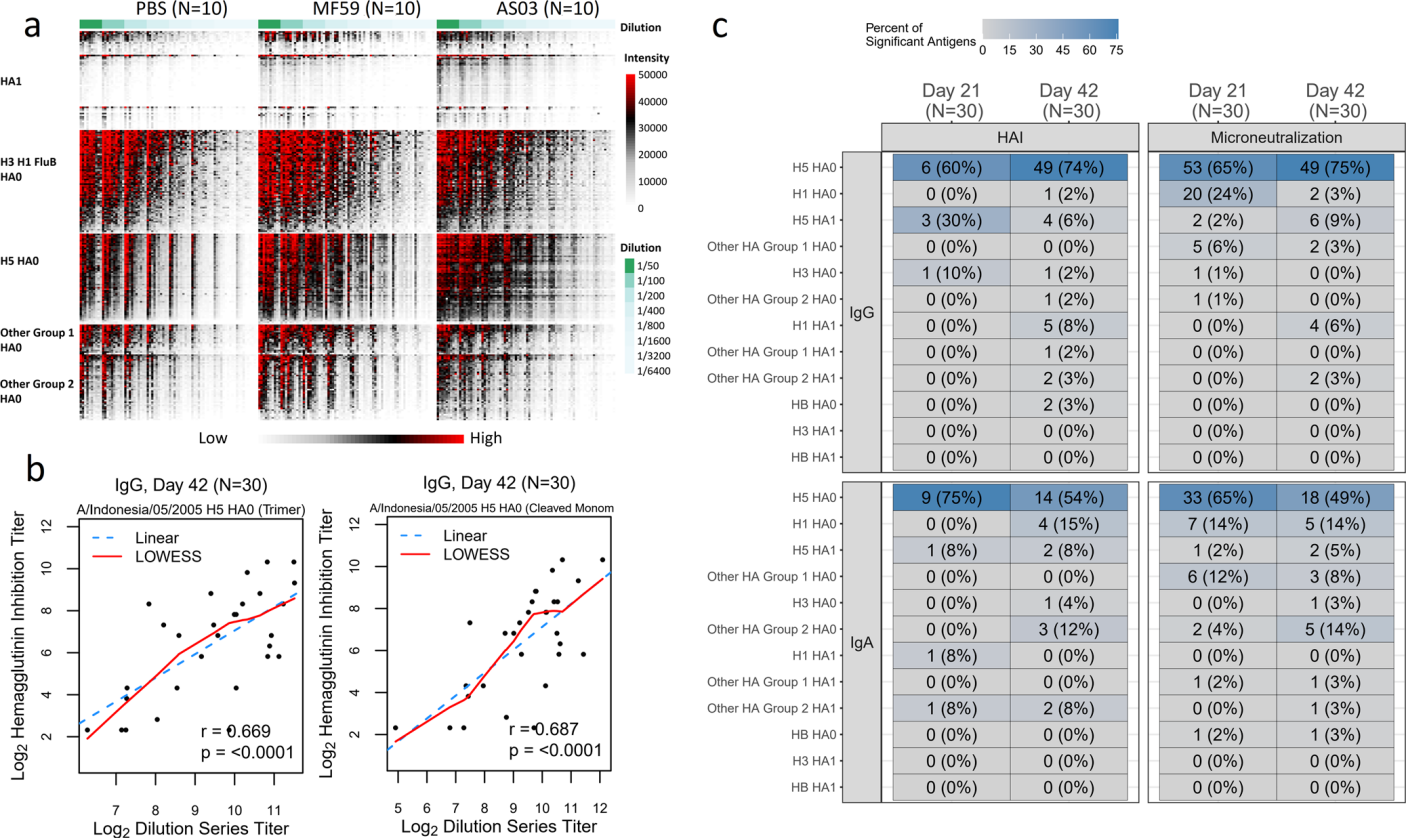
Array-based titers determined in an 8 dilution-series experiment for vaccine antigens and antigens belonging to the same subtype as the vaccine antigen were significantly correlated with HAI and microneutralization titers. **a** Array results for eight dilutions for 60 sera. **b** Scatter plots of day 42 log_2_ dilution series-based titer and log_2_ hemagglutinin inhibition and log_2_ microneutralization titer for influenza A/Indonesia/05/2005 antigens across vaccine groups (*n* = 30) for HA0 trimer (left) and cleaved monomer (right). **c** The number and percentage of antigens with statistically significant Pearson correlations (*p* < 0.05) by assay, molecule, and subtype.

To get a more robust assessment of antibody responses, we determined titers for a subset of sera from 30 subjects (10 per vaccine group) and two timepoints (day 21 and 42) (Fig. 1a, Fig. 10a). For each serum specimen, we performed eight twofold serial dilutions. Titers estimated from these serial-dilution experiments were assessed for correlation against the known hemagglutination inhibition (HAI) and microneutralization (MN) titers in the samples. We first compared the calculated titers from the four HA0 antigens on the array that matched the vaccine HA (H5 A/Indonesia/05/2005). The IgG dilution-based titers for H5 A/Indonesia/05/2005 correlated strongly with microneutralization titers at day 21 (*r*_s_: 0.51–0.56) and 42 (*r*_s_: 0.57–0.80) and with HAI titers at day 42 (*r*_s_: 0.49 to 0.74). Day 42 associations for a trimeric HA0 and the cleaved version of a monomer are presented in Fig. 10b. In all these cases, associations were statistically significant (*p* < 0.05), whereas, the IgA dilution-based titers correlations were less strong and were more dependent on the confirmation of the vaccine antigen analyzed (HAI or MN *r*_s_: 0.31–0.51 for day 21 and *r*_s_: 0.43–0.63) with only the trimeric HA0 reaching statistical significance at day 42 (*r* = 0.63, *p* < 0.05). All day 42 associations are shown in Supplementary Fig. 6.

Next, we assessed the correlation patterns across all antigens (Fig. 10c). Assessment of statistically significant correlations (*p* < 0.05) across HA subtypes showed that these antigens were predominantly H5 full-length antigens which were of the same subtype as the vaccine (Fig. 10c). For IgG, the percentage of correlated responses among all H5 full-length antigens on the array increased from 60% to 74% for HAI following the second dose indicating an increase of 14% in correlated responses. For microneutralization, an increase from 65% to 75% was observed. In contrast, IgA dilution-based titers showed the broadest correlation against H5 full-length antigens on day 21 which decreased from 75% to 54% of all H5 full-length antigens on the array by HAI and 65% to 49% by MN on day 42.

## Discussion

Despite repeated exposures to seasonal influenza viruses and no known exposure to avian H5N1 viruses, our study participants’ serum exhibited antibodies with broad reactivity against all the subtypes of full-length HA represented on both arrays at baseline prior to immunization. Our data demonstrated that a large proportion of these baseline antibodies were directed against the stalk, presumably resulting from prior infection or vaccination. Prior exposure to seasonal viruses may also have elicited antibody against the N-terminus of HA1, containing a conserved 115 amino-acid sequence. These N-terminus HA1 antibody responses would not be diminished with the mini-HA blocking experiments because they were not included in the mini-HA structure. Existence of these antibodies also implied the existence of memory B cells that are highly cross-reactive.

Our results confirmed that the A/Indonesia/05/2005 H5N1 vaccine when given without an adjuvant-induced limited antibody responses. This is likely a combination of the poor immunogenicity of avian HAs as well as the lack of innate immune signaling induced by adjuvants that enhance antigen processing and presentation^23–25^. The poor immunogenicity of unadjuvanted avian influenza vaccines is well documented^23^. One theory is that the head domains of avian (i.e., H5) HAs are so antigenically distinct from seasonal H1, that the head lacks available T cell epitopes which could provide CD4 T cell help/Tfh to B cells^26,27^. The germinal center response is capable of producing IgA response when the correct innate signals from the adjuvanted vaccine are present^28,29^. Alternatively, immune responses specific to the head depend on CD4 help, whereas the anti-stalk response is predominantly from the cross-reactive memory pool.

The H5 HA1 response was low at baseline, slightly increased after the first dose of vaccine (prime), and significantly increased after the second dose (boost) in the context of an adjuvant, most markedly with AS03. In contrast, IgG and IgA responses against H5 HA0 increased after the first dose but were not significantly further increased with the second dose and the H5 HA0 response to the first dose was much more pronounced for the adjuvanted groups relative to the unadjuvanted group for IgA. To account for this differential boosting response, we speculate that the H5N1 HA1 is a novel variant antigen that has not been previously exposed to the immune system^30^. The priming dose is necessary to produce a memory response which is evident in the anamnestic response measured after the boost. Conversely, limited responses for H5N1 HA0 which is conserved across subtypes post the second dose, maybe due to their anti-stalk prime and memory from prior seasonal exposures. The anti-stalk antibody response is anamnestic after the first dose drawing on the memory pool and no further increase is seen after the boost. At baseline, about 50% of the responses against full-length H5 and approximately 25% against full-length H1 could be blocked by competitive inhibition with an H1 stalk trimer, providing evidence that a substantial percentage of the antibody against H5 HA0 was directed against the highly conserved stalk region^31–34^.

Sixty specimens from this study were serially diluted 8 times to produce titration curves. Midpoint titers estimated from these serial-dilution experiments were assessed for correlation with the HAI and MN titers. Statistically significantly correlated antigens were present in predominantly H5 HA0 antigens of the same subtype as the vaccine. The fact that microarray-based dilution titers for vaccine antigens were statistically significantly associated with HAI and MN titer at day 42 lends additional confidence to these results and hints at their potential use as surrogates for HAI and MN titers.

Subtype and drift variant virus specificity experienced with the H5N1 vaccine and with seasonal influenza vaccines may be related to the low level of heterosubtypic antibodies induced by unadjuvanted vaccines and with natural infection. The use of these potent immune-stimulating adjuvants demonstrated IgG and IgA levels which were high and more broadly reactive across drifted variants and subtypes than the unadjuvanted vaccine. This may translate to improved protective efficacy that is less prone to virus variants. Even when considering vaccine-mediated subtype-specific responses and vaccine efficacy, unadjuvanted seasonal influenza vaccination only demonstrated 50% effectiveness^35,36^. The MF59 adjuvated seasonal influenza vaccine (FluAd^®^) demonstrated some improved effectiveness compared to non-adjuvanted vaccines^37,38^. McElhaney *et al*. also report that the AS03-adjuvanted trivalent influenza vaccine in a phase 3 clinical trial resulted in improved outcomes compared to the unadjuvanted vaccine. Our data provide further evidence for both the AS03^39^ and MF59^22^ adjuvant as an effective means of eliciting a higher and broader antibody response compared to the unadjuvanted vaccine. While each adjuvant has previously been shown to enhance antibody responses post-vaccination^11,22,40^, our results add additional details comparatively assessing their breadth relative to the unadjuvanted vaccine using protein microarrays. Even though the two parallel parent clinical studies from which our specimens were derived were not originally designed to directly compare the performance of these two different adjuvants, the clinical protocols are sufficiently similar such that the antibody responses we describe can be generalized and compared. Although both adjuvants appeared to broaden the antibody responses predominantly to phylogenetically related HA proteins, those closely related to the vaccine strain as was evident by the antibody landscape results, AS03 elicited statistically significantly broader heterosubtypic antibody responses, compared to MF59 recognizing statistically significantly more non-H5 HA antigens post second dose. Furthermore, anti-stalk competitive inhibition and blocking experiments indicated that some of these broad antibody responses were directed against the conserved stem region of group 1 HA0s, in particular H1. These results seem to be in contrast with Khurana et al. showing from whole-genome gene fragment phage display libraries (GFPDLs) that for both MF59 and AS03, the adjuvant effect manifested itself mostly as a broader HA1 response with limited impact on HA2^30,41,42^ We attribute this to the lack of stabilized trimeric molecules in phage display libraries. Interestingly, the AS03-adjuvanted group showed an increased percentage in anti-stalk competition for IgG for other group 1 HA0 and HB antigens after second dose relative to the MF59 group which showed a decrease. However, the difference was not statistically significant with our limited sample size warranting larger studies to assess if these broader anti-stalk responses following AS03-adjuvanted vaccination can be generalized.

Results of this study inform efforts to the development of a universal influenza vaccine. While there are a number of universal vaccine candidates which are being developed using non-HA-based strategies (e.g., M2, nucleoprotein, neuraminidase), a large proportion of strategies are focused on conserved regions of HA^43–46^. Our data indicated that potent immune-stimulating adjuvants can improve the antibody responses elicited by standard subvirion vaccines through eliciting more homo- and heterosubtypic antibody. We observed that stalk-directed IgG and IgA antibody was primarily elicited with the first dose. If the elicitation of stalk-directed antibody were the key to protection through a universal influenza vaccine construct, then it may be essential to accompany the first dose with a potent adjuvant.

The main limitation of this study is the relatively small sample size for the serial-dilution and anti-stalk experiments (*n* = 10 for each group). We utilized two arrays that each have certain limitations. While Array 1 contained a wider variety of HA1 antigens, all its HA0 antigens were monomers. In contrast, Array 2 contained fewer HA1 antigens (63 vs. 122) but more HA0 antigens (174 vs. 153) of which 80 (46%) were trimers. We presented results for both arrays so that differences can be contrasted.

In summary, while both adjuvants greatly boosted antibody responses to H5 HA1 after 2 doses, there is evidence in the data of an increased heterosubtypic breadth as well as homosubtypic H5 head antibodies associated with AS03 relative to MF59 adjuvanted vaccination. Nonetheless, our study cannot substantiate an increase in the clinical efficacy of vaccination with AS03 for an H5N1 pandemic vaccine over MF59. In addition, our results highlight the importance of measuring dominant conformational epitopes present in trimeric HA. Together, these results support future clinical studies that utilize vaccine adjuvants to develop more effective, and more universally-reactive influenza vaccines.

## Methods

### Study specimen

The serum samples originated from future-use consenting participants of parallel avian A(H5N1)/Indonesia/05/2005 influenza vaccine mix and match studies involving MF59^®22^ and AS03^11,40^ adjuvant, also known as DMID Protocols 10-0016 and 10-0017. Only the participants that received the 15 µg dose of H5 vaccine were selected for the microarray analysis; three timepoints from each participant were selected and represent baseline (day 0 or pre-vaccination), day 21 (post-first dose of vaccine), and day 42 (21 days post second dose of vaccine).

### Adjuvant

MF59 is an oil-in-water emulsion trademark owned by or licensed to Novartis and AS03 is an adjuvanted system containing DL-α-tocopherol and squalene in an oil-in-water emulsion owned by GlaxoSmithKline.

### Clinical trials registration

NCT01317758 and NCT01317745.

### Experiment

Each serum sample was diluted in protein array blocking buffer (GVS, Sanford, ME) supplemented with *E. coli* lysate (GenScript, Piscataway, NJ) to a final concentration of 10 mg/mL, and pre-incubated at room temperature (RT) for 30 min. Concurrently, arrays were rehydrated in blocking buffer (without lysate) for 30 min. Blocking buffer was removed, and arrays were probed with pre-incubated serum samples using sealed chambers to ensure no cross-contamination of samples between pads. Arrays were incubated overnight at 4 °C with gentle agitation. Arrays were then washed at RT five times with TBS-0.05% Tween 20 (T-TBS), followed by incubation with QDot®-conjugated goat anti-human IgG/ IgA diluted 1:200 in blocking buffer for 2 h at RT. After incubation in secondary antibodies, arrays were then washed three times with T-TBS and once with water. Chips were air dried by centrifugation at 1000×*g* for 5 min and scanned on an ArrayCam^TM^ 400-S Microarray Imaging System from Grace Bio-Labs (Bend, OR) for QDot®. Spot and background intensities were measured using an annotated grid (gal) file. For ArrayCam^TM^ a number of different settings were used to attempt to obtain readings. Microarray spot intensities were quantified using software ArrayCam^TM^ (Grace Bio-Labs) utilizing automatic local background subtraction for each spot. The generated signal intensity values were considered raw values.

### Protein microarray composition

The first array primarily contained monomeric HA (Array #1; Supplementary Table 2) and the second array consisted of both monomeric and trimeric HA (Array #2; Supplementary Table 3). Array #1 used for the first probing experiment (Exp 1) was composed of 284 antigens including 279 unique influenza HA antigens: 94 full-length HA (HA0; HA1 + HA2) and 80 HA1 from group 1 (including H5) influenza viruses; and 56 HA0 and 38 HA1 subunits from group 2 influenza viruses. Among the group 1 antigens were 44 HA0 and 36 HA1 subunits from H5 viruses (the same subtype as the vaccine strain). In addition, there were 4 HA2 subunit antigens (2 × H5, 1 × FluB, 1 × H1, which contain most of the stalk, although misfolded), 4 NA, and 1 NP protein on Array #1. These proteins were all acquired from Sino Biologicals. Array #2 used in the second probing experiment (Exp 2) was composed of 270 unique influenza proteins including 239 HA proteins, representing 161 (57%) proteins that were included in Array #1 (Sino Biologicals), plus 80 HA0 trimers and 22 NA proteins, provided by the Dr. F. Krammer laboratory and Dr. J. Crowe laboratory. Array #2 contained 95 HA0 and 45 HA1 subunits from group 1 influenza viruses and 69 HA0 and 17 HA1 subunits from group 2 influenza viruses. Among the group 1 antigens were 50 HA0 and 33 HA1 subunits from H5 viruses. In addition, 2 HA2 antigens from H5 viruses, 1 NP, and 30 NA proteins were present on Array #2.

### Data normalization

Prior to the analysis, systematic intensity differences in background reactivity as measured by phosphate-buffered saline with tween 20 (PBST) were corrected. This was achieved by subtracting the median raw intensity signal for the 24 PBST intensity measurements for a certain array from the array’s non-PBST antigen intensities. Following background correction, for intensities <1, an intensity value of 1 was imputed.

### Identification of significant adjuvant-boosted antibody responses

For each antigen, an ANOVA model was fit separately to day 21 and day 42 log_2_ fold change (LFC) responses from day 0 as part of the first and second probing experiments. The model was specified to describe the LFC in antibody signal for a certain antigen as a function of vaccine group (fixed effect with three levels: A/H5N1 HA + AS03, A/H5N1 HA + MF59, and A/H5N1 HA + PBS) and receipt of seasonal influenza vaccine during the past two years (fixed effect with two levels: yes, no). The second fixed effect was added to adjust antibody responses for seasonal influenza vaccine effects. Contrasts as implemented in the *lsmeans* R package (Version 2.27.62) were used to assess statistical significance of the mean LFC difference between the adjuvanted vaccine groups (A/H5N1 HA + AS03 and A/H5N1 HA + MF59) and the unadjuvanted control group (A/H5N1 HA + PBS) adjusted for prior receipt of vaccine (*H*_0_: *μ*_adjuvanted vaccine group_ −*μ* _unadjuvanted control group_ = 0, *H*_1_: *μ*_adjuvanted vaccine group_ − *μ*_unadjuvanted control group_ ≠ 0, on the log_2_ scale). To control for testing multiple antigens, a false-discovery rate (FDR) based on the Benjamini-Hochberg procedure as implemented in the R p.adjust function was applied separately for each post-vaccination day (day 21 and 42) and vaccine group comparison. Antibody responses with an FDR-adjusted *p* value <0.1 were deemed statistically significant.

### Determination of percent anti-stalk competition

We used a stabilized trimeric mini H1 HA recombinant stalk molecule^47–49^ (#4900), produced by Dr. L. Coughlan laboratory, which lacks the entire HA1 head including the HA1 segment that interacts with the stalk. As a negative control, we used a pre-fusion stabilized trimeric RSV-F protein (DS-Cav1 5K6I, also produced by Dr. L. Coughlan laboratory)^50^, We performed anti-stalk experiments for a subset of sera from 30 subjects (10 per vaccine group) that were probed on Array #2. Percent stalk competition (PSC) was calculated as: 100−(AUC with competition (mini-HA added)/AUC without competition (RSV-F added) × 100) where the AUC represented the area under the curve for two different concentrations of mini-HA and RSV-F (10 and 50 µg/mL). The AUC was calculated using the trapezoid rule implemented in R. To prevent negative values, for PSCs that were <1%, a value of 1% was imputed.

### Determination of antibody titers from serial-dilution experiments

Dilution experiments consisted of eight serial twofold dilutions (0.04, 0.02, 0.01, 0.005, 0.0025, 0.00125, 0.000625, 0.0003125) for day 21 and 42 specimens for a subset of 30 subjects (10 per vaccine group) that were probed on Array #2. This information was used to estimate the half maximal effective dilution, referred to as a titer. For each sample, the titer was determined using the following binding model $A + B\mathop { \to }\limits^{\rm{yields}} AB$ where A is the antigen and B is the antibody. The functional relationship between intensity (*y*) and dilution (*x*) was described using the Michaelis-Menten equation: $y = \frac{{{{{I}}}_{\rm{max}} \times x}}{{{{{K}}}_{\mathrm{d}} + x}}$. The two model parameters (*I*_max_ and *K*_d_) were then estimated by fitting non-linear models as implemented in the *nls* R package. The resulting titer per sample was determined as 1/*K*_d_ which represented the titer estimate at which half the level of *I*_max_ was reached.

### Reporting summary

Further information on research design is available in the Nature Research Reporting Summary linked to this article.

## Supplementary information

Supplementary Figures

REPORTING SUMMARY

## Data Availability

The data were made available via the GEO database (accession GSE202392).

## References

[1] Kerstetter LJ, Buckley S, Bliss CM, Coughlan L (2021). Adenoviral vectors as vaccines for emerging avian influenza viruses. Front. Immunol..

[2] Subbarao K, Joseph T (2007). Scientific barriers to developing vaccines against avian influenza viruses. Nat. Rev. Immunol..

[3] Fouchier RAM (2004). Avian influenza A virus (H7N7) associated with human conjunctivitis and a fatal case of acute respiratory distress syndrome. Proc. Natl Acad. Sci. USA.

[4] Jennings LC, Monto AS, Chan PK, Szucs TD, Nicholson KG (2008). Stockpiling prepandemic influenza vaccines: a new cornerstone of pandemic preparedness plans. Lancet Infect. Dis..

[5] Belshe RB (2011). Safety and immunogenicity of influenza A H5 subunit vaccines: effect of vaccine schedule and antigenic variant. J. Infect. Dis..

[6] Nicholson KG (2001). Safety and antigenicity of non-adjuvanted and MF59-adjuvanted influenza A/Duck/Singapore/97 (H5N3) vaccine: a randomised trial of two potential vaccines against H5N1 influenza. Lancet.

[7] Garçon N, Vaughn DW, Didierlaurent AM (2012). Development and evaluation of AS03, an adjuvant system containing α-tocopherol and squalene in an oil-in-water emulsion. Expert Rev. Vaccines.

[8] Ott G, Radhakrishnan R, Fang J-H, Hora M (2003). The adjuvant MF59: a 10-year perspective. Vaccine Adjuv..

[9] Jackson LA (2015). Effect of varying doses of a monovalent H7N9 influenza vaccine with and without AS03 and MF59 adjuvants on immune response a randomized clinical trial. JAMA.

[10] Belshe RB (2014). Immunogenicity of avian influenza A/Anhui/01/2005(H5N1) vaccine with MF59 adjuvant a randomized clinical trial. JAMA.

[11] Chen, W. H. et al. Safety, reactogenicity, and immunogenicity of inactivated monovalent influenza A(H5N1) virus vaccine administered with or without AS03 adjuvant. *Open Forum Inf. Dis.*10.1093/ofid/ofu091 (2014). 10.1093/ofid/ofu091 PMC4324222 25734159

[12] Angeletti D (2017). Defining B cell immunodominance to viruses. Nat. Immunol..

[13] Altman MO, Angeletti D, Yewdell JW (2018). Antibody immunodominance: the key to understanding influenza virus antigenic drift. Viral Immunol..

[14] Henry Dunand CJ (2016). Both neutralizing and non-neutralizing human H7N9 influenza vaccine-induced monoclonal antibodies confer protection. Cell Host Microbe.

[15] Tan GS (2016). Broadly-reactive neutralizing and non-neutralizing antibodies directed against the H7 influenza virus hemagglutinin reveal divergent mechanisms of protection. PLoS Pathog..

[16] Coughlan L, Palese P (2018). Overcoming barriers in the path to a universal influenza virus vaccine. Cell Host Microbe.

[17] Ng S (2019). Novel correlates of protection against pandemic H1N1 influenza A virus infection. Nat. Med..

[18] Paules CI, Marston HD, Eisinger RW, Baltimore D, Fauci AS (2017). The pathway to a universal influenza vaccine. Immunity.

[19] Nakajima R (2018). Protein microarray analysis of the specificity and cross-reactivity of influenza virus hemagglutinin-specific antibodies. mSphere.

[20] Desbien AL (2013). Development of a high density hemagglutinin protein microarray to determine the breadth of influenza antibody responses. Biotechniques.

[21] Van Hoeven, N. et al. A formulated TLR7/8 agonist is a flexible, highly potent and effective adjuvant for pandemic influenza vaccines. *Sci. Rep.*10.1038/srep46426 (2017). 10.1038/srep46426 PMC5399443 28429728

[22] Mulligan, M. J. et al. Point-of-Use Mixing of Influenza H5N1 Vaccine and MF59 Adjuvant for Pandemic Vaccination Preparedness: Antibody Responses and Safety. A Phase 1 Clinical Trial. *Open forum infectious diseases*10.1093/ofid/ofu102 (2014). 10.1093/ofid/ofu102 PMC4324215 25734170

[23] Howard LM (2017). Cell-based systems biology analysis of human AS03-adjuvanted H5N1 avian influenza vaccine responses: a phase i randomized controlled trial. PLoS One.

[24] Del Giudice G, Rappuoli R, Didierlaurent AM (2018). Correlates of adjuvanticity: a review on adjuvants in licensed vaccines. Semin. Immunol..

[25] Nguyen-Contant, P., Sangster, M. Y. & Topham, D. J. Squalene-based influenza vaccine adjuvants and their impact on the hemagglutinin-specific b cell response. *Pathogens*10.3390/pathogens10030355 (2021). 10.3390/pathogens10030355 PMC8002393 33802803

[26] DiPiazza AT (2019). A novel vaccine strategy to overcome poor immunogenicity of avian influenza vaccines through mobilization of memory CD4 T cells established by seasonal influenza. J. Immunol..

[27] De Groot AS (2013). Low immunogenicity predicted for emerging avian-origin H7N9: Implication for influenza vaccine design. Hum. Vaccines Immunother..

[28] Moris P (2011). H5N1 influenza vaccine formulated with AS03 A induces strong Cross-reactive and polyfunctional CD4 T-Cell responses. J. Clin. Immunol..

[29] Van Der Most RG (2014). Influenza: Seeking help: B cells adapting to flu variability. Sci. Transl. Med..

[30] Khurana S (2009). Antigenic fingerprinting of H5N1 avian influenza using convalescent sera and monoclonal antibodies reveals potential vaccine and diagnostic targets. PLoS Med..

[31] Nachbagauer R (2014). Induction of broadly reactive anti-hemagglutinin stalk antibodies by an H5N1 vaccine in humans. J. Virol..

[32] Ellebedy AH (2014). Induction of broadly cross-reactive antibody responses to the influenza HA stem region following H5N1 vaccination in humans. Proc. Natl Acad. Sci. USA.

[33] Ellebedy AH (2020). Adjuvanted H5N1 influenza vaccine enhances both cross-reactive memory B cell and strain-specific naive B cell responses in humans. Proc. Natl Acad. Sci..

[34] Nachbagauer, R. et al. Pandemic influenza virus vaccines boost hemagglutinin stalk-specific antibody responses in primed adult and pediatric cohorts. *NPJ Vaccines*10.1038/s41541-019-0147-z (2019). 10.1038/s41541-019-0147-z PMC6898674 31839997

[35] Rose A (2020). Interim 2019/20 influenza vaccine effectiveness: six European studies, September 2019 to January 2020. Eurosurveillance.

[36] Dawood FS, Chung JR, Kim SS, Zimmerman RK, Nowalk MP (2020). Interim estimates of 2019 – 20 seasonal influenza vaccine effectiveness —United States, february 2020. Morb. Mortal. Wkly. Rep..

[37] Van Buynder PG (2013). The comparative effectiveness of adjuvanted and unadjuvanted trivalent inactivated influenza vaccine (TIV) in the elderly. Vaccine.

[38] Mannino S (2012). Effectiveness of adjuvanted influenza vaccination in elderly subjects in northern Italy. Am. J. Epidemiol..

[39] McElhaney JE (2013). AS03-adjuvanted versus non-adjuvanted inactivated trivalent influenza vaccine against seasonal influenza in elderly people: a phase 3 randomised trial. Lancet Infect. Dis..

[40] Chen WH (2016). Persistence of antibody to influenza A/H5N1 vaccine virus: impact of AS03 adjuvant. Clin. Vaccine Immunol..

[41] Khurana, S. et al. Vaccines with MF59 adjuvant expand the antibody repertoire to target protective sites of pandemic avian H5N1 influenza virus. *Sci. Transl. Med.*10.1126/scitranslmed.3000624 (2010). 10.1126/scitranslmed.3000624 20371470

[42] Khurana, S. et al. MF59 adjuvant enhances diversity and affinity of antibody-mediated immune response to pandemic influenza vaccines. *Sci. Transl. Med.*10.1126/scitranslmed.3002336 (2011). 10.1126/scitranslmed.3002336 PMC3501657 21632986

[43] Sautto, G. A., Kirchenbaum, G. A. & Ross, T. M. Towards a universal influenza vaccine: different approaches for one goal. *Virol. J.*10.1186/s12985-017-0918-y (2018). 10.1186/s12985-017-0918-y PMC5785881 29370862

[44] Darricarrère, N. et al. Broad neutralization of H1 and H3 viruses by adjuvanted influenza HA stem vaccines in nonhuman primates. *Sci. Transl. Med.*10.1126/scitranslmed.abe5449 (2021). 10.1126/scitranslmed.abe5449 33658355

[45] Bullard, B. L. & Weaver, E. A. Strategies targeting hemagglutinin as a universal influenza vaccine. *Vaccines*10.3390/vaccines9030257 (2021). 10.3390/vaccines9030257 PMC7998911 33805749

[46] Adar Y (2009). A universal epitope-based influenza vaccine and its efficacy against H5N1. Vaccine.

[47] Nachbagauer R (2021). A chimeric hemagglutinin-based universal influenza virus vaccine approach induces broad and long-lasting immunity in a randomized, placebo-controlled phase I trial. Nat. Med..

[48] Carreño, J. M. et al. Development and assessment of a pooled serum as candidate standard to measure influenza A virus group 1 hemagglutinin stalk-reactive antibodies. *Vaccines*10.3390/vaccines8040666 (2020). 10.3390/vaccines8040666 PMC7712758 33182279

[49] Antonietta I (2015). A stable trimeric influenza hemagglutinin stem as a broadly protective immunogen. Science.

[50] Joyce MG (2016). Iterative structure-based improvement of a fusion-glycoprotein vaccine against RSV. Nat. Struct. Mol. Biol..

[51] Russell RJ (2008). Structure of influenza hemagglutinin in complex with an inhibitor of membrane fusion. Proc. Natl Acad. Sci. USA.

