# Supplementary material for: The antibody landscapes following AS03 and MF59 adjuvanted H5N1 vaccination
Source: NPJ Vaccines. 2022 Aug 30;7:103. doi: 10.1038/s41541-022-00524-7 (PMC9427073; doi:10.1038/s41541-022-00524-7)
Supplement: Supplementary file 1 — Supplementary Figures [file 41541_2022_524_MOESM1_ESM.pdf]

Supplementary Information for "The antibody landscapes against Group 1 and 2 influenza virus hemagglutinin following AS03 and MF59 adjuvanted H5N1 vaccination"

Supplementary Figure 1 Comparison of trimeric vs. non-trimeric HA0 response by subtype (Array 2, Experiment 2). HA0: antibody responses against full length HA proteins. Antibody reactivity of monomeric HA0s was compared with stabilized trimeric HA0s. Each boxplot summarizes the geometric mean intensity across all samples by antigen type (trimeric vs. monomeric) irrespective of post-vaccination day and vaccine group. N represents the number of antigens. Results within subjects for multiple antigens were aggregated using the geometric mean intensity. The horizontal line in each boxplot represents the median.

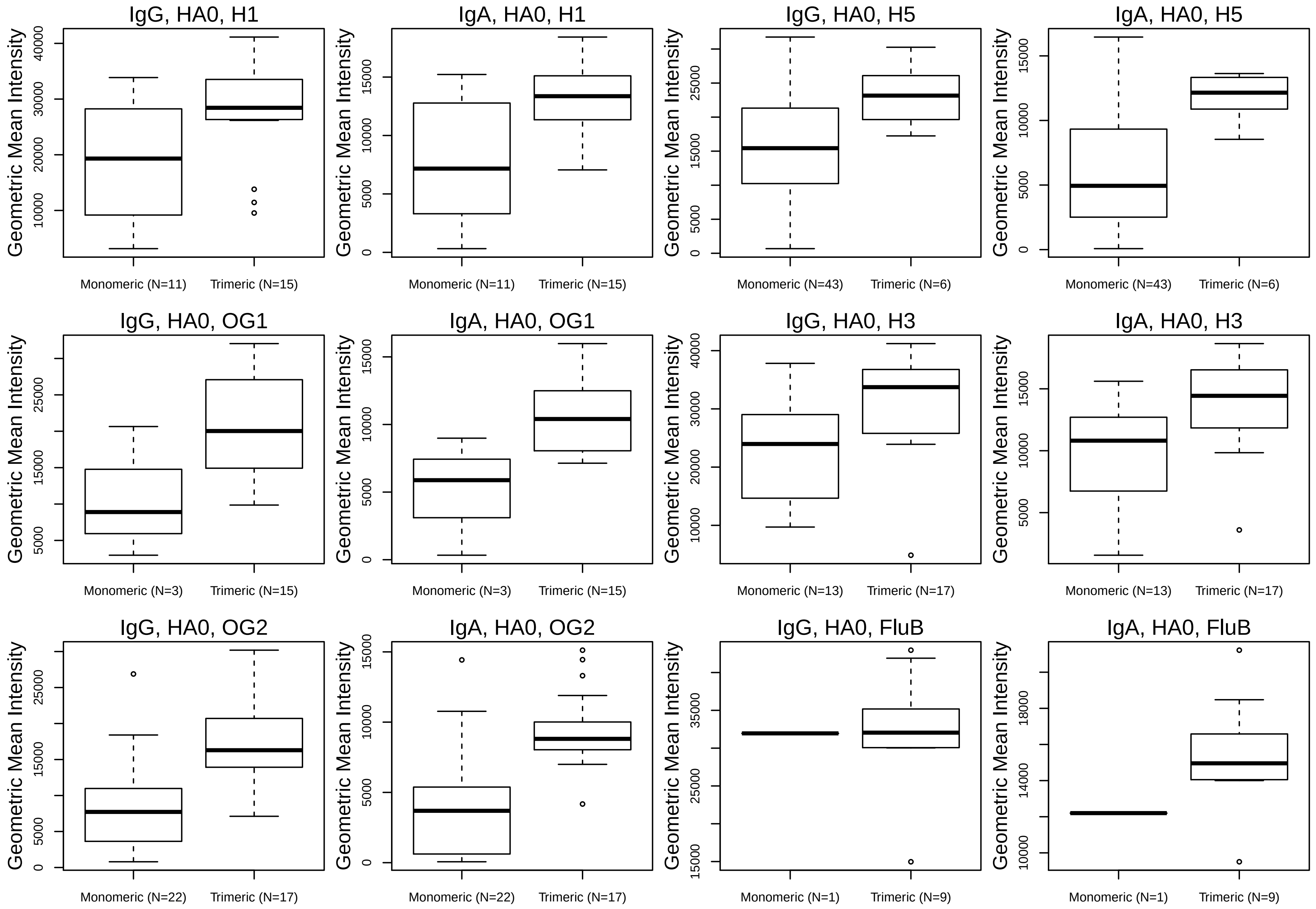

Supplementary Figure 2A Pre-vaccination geometric mean intensity by HA subtype (Array 1, Experiment 1). Each boxplot summarizes pre-vaccination geometric mean intensities by HA subtype across all 130 subjects. Results within subjects for multiple antigens of the same HA subtype were aggregated using the geometric mean. HA1: antibody responses against head HA proteins, HA0: antibody responses against full length HA proteins. The horizontal line in each boxplot represents the median.

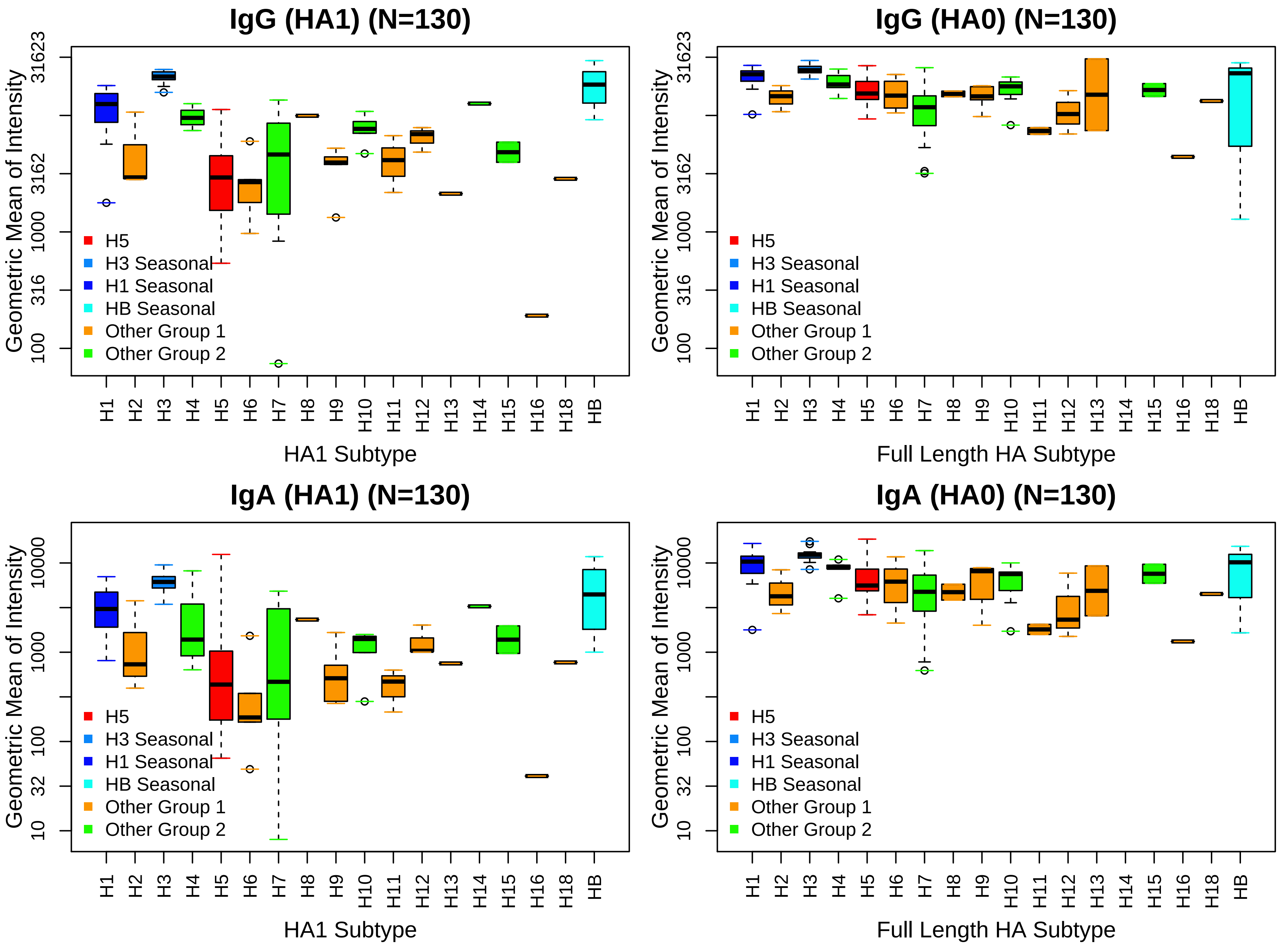

Supplementary Figure 2A Pre-vaccination geometric mean intensity by HA subtype (Array 1, Experiment 1). Each boxplot summarizes pre-vaccination geometric mean intensities by HA subtype across all 130 subjects. Results within subjects for multiple antigens of the same HA subtype were aggregated using the geometric mean. HA1: antibody responses against head HA proteins, HA0: antibody responses against full length HA proteins. The horizontal line in each boxplot represents the median.

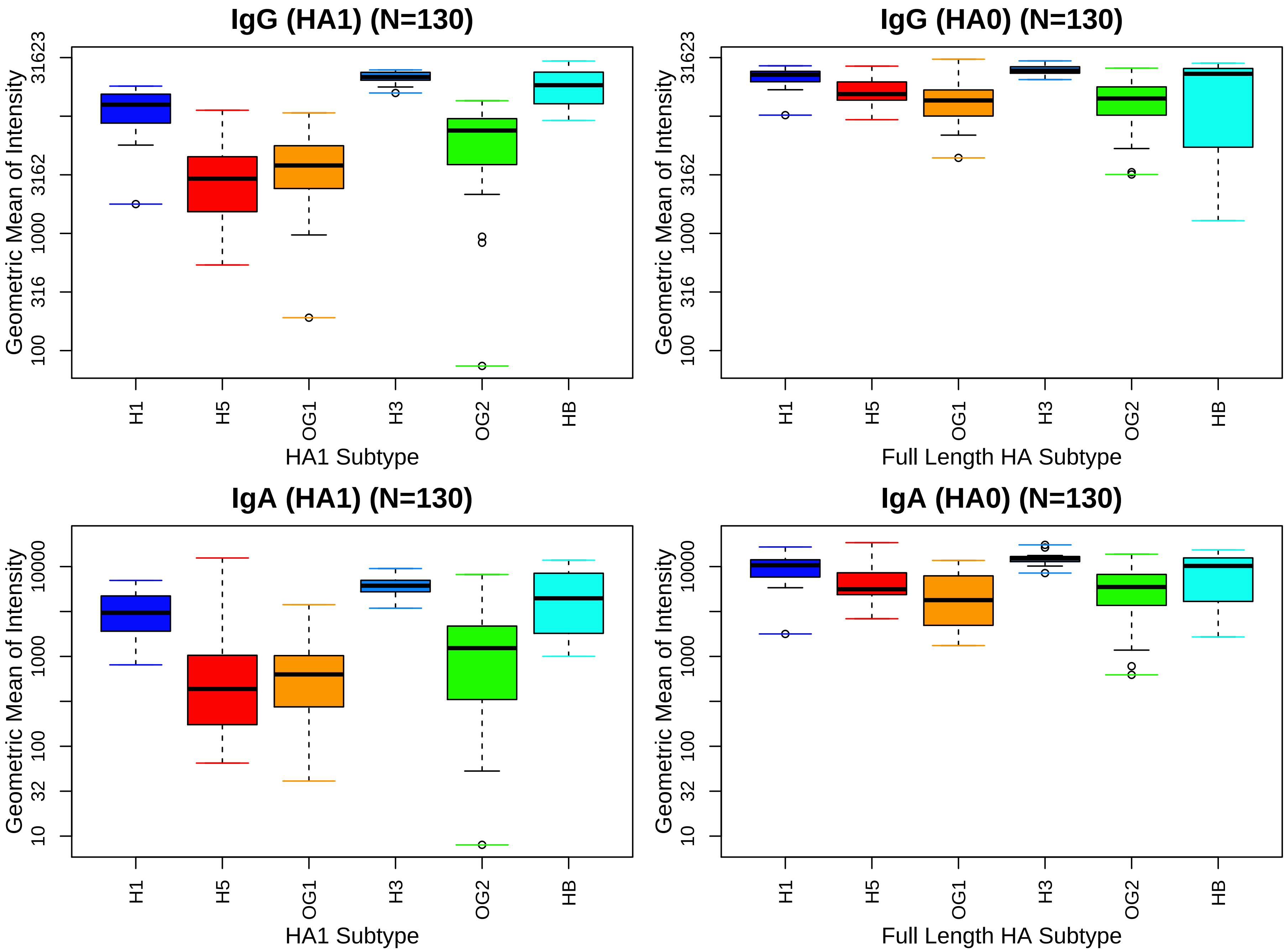

Supplementary Figure 2B Pre-vaccination geometric mean intensity by HA subtype (Array 2, Experiment 2). Each boxplot summarizes pre-vaccination geometric mean intensities by HA subtype across all 130 subjects. Results within subjects for multiple antigens of the same HA subtype were aggregated using the geometric mean. HA1: antibody responses against head HA proteins, HA0: antibody responses against full length HA proteins. The horizontal line in each boxplot represents the median.

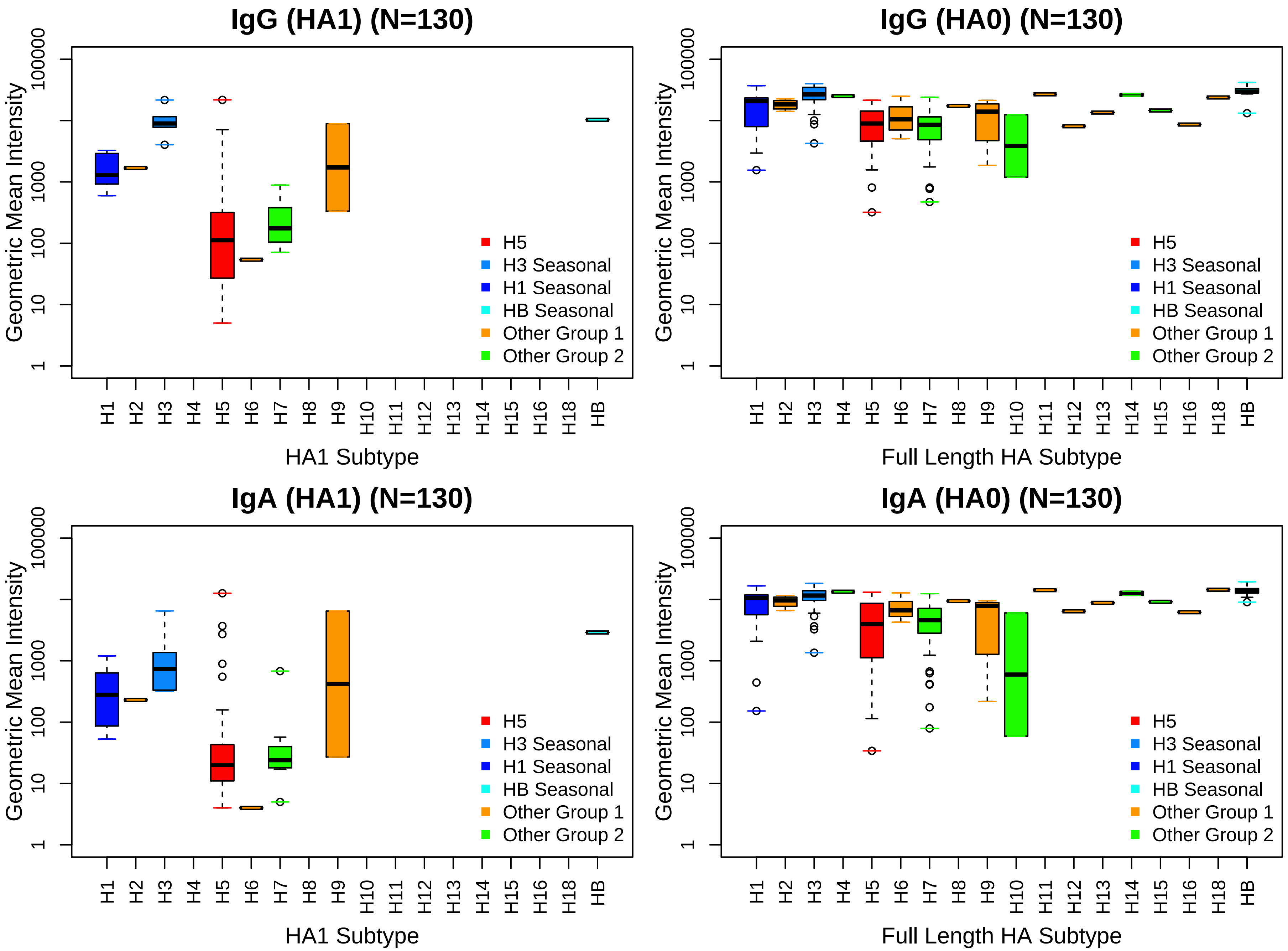

Supplementary Figure 2B Pre-vaccination geometric mean intensity by HA subtype (Array 2, Experiment 2). Each boxplot summarizes pre-vaccination geometric mean intensities by HA subtype across all 130 subjects. Results within subjects for multiple antigens of the same HA subtype were aggregated using the geometric mean. HA1: antibody responses against head HA proteins, HA0: antibody responses against full length HA proteins. The horizontal line in each boxplot represents the median.

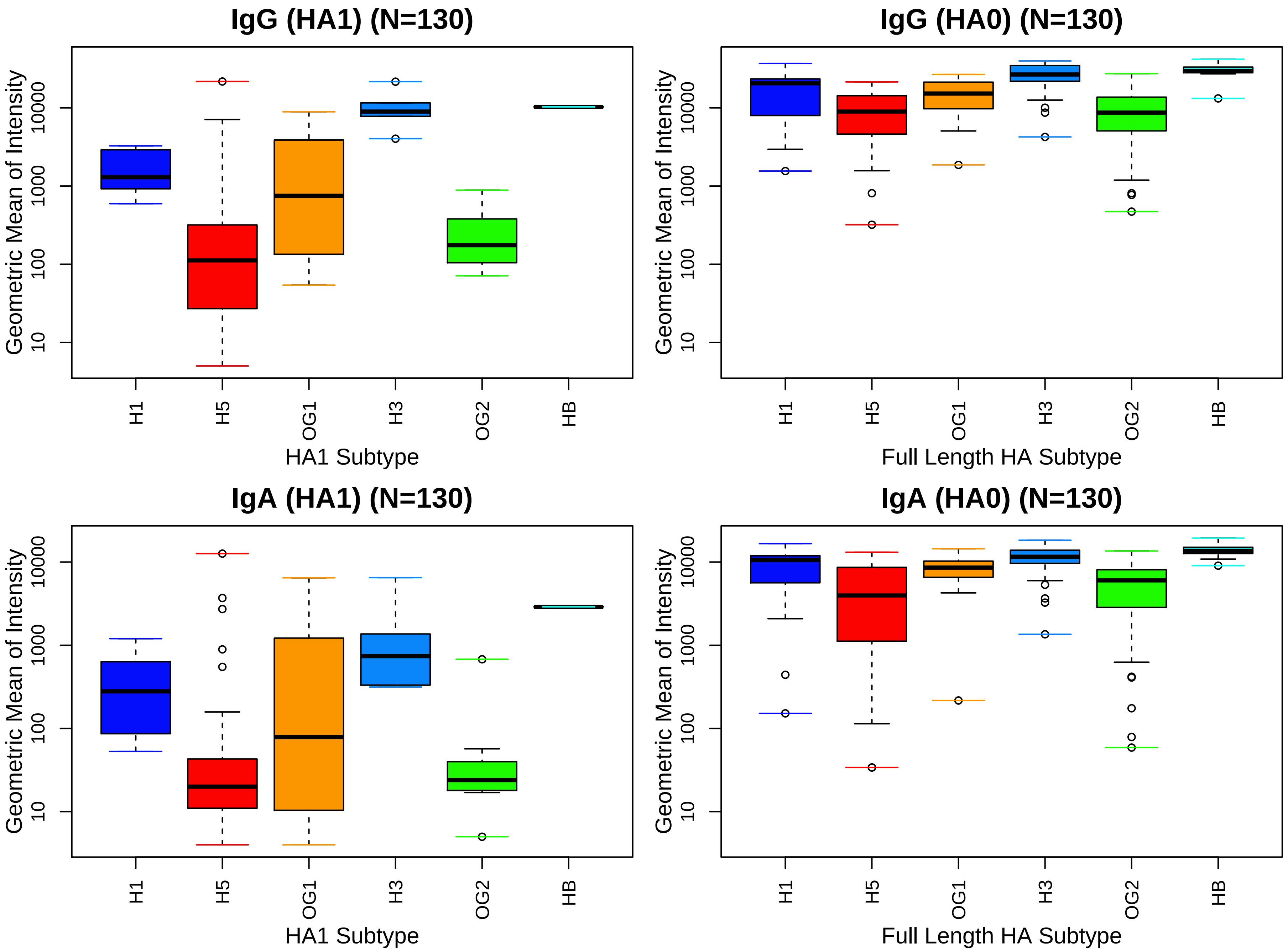

Supplementary Figure 3A Impact of prior seasonal influenza vaccination on H5 HA vaccination effects (Array 1, Experiment 1). Each boxplot summarizes the median log_2 fold change antibody responses across H5 HAs by vaccination status (subject receiving seasonal prior vaccinations within the past 2 years or those not receiving seasonal influenza vaccine within that time frame) and vaccine group for each antibody type (IgG and IgA) and post-vaccination day. Results within subjects for multiple H5 HA antigens were aggregated using the log_2 median fold change. The horizontal line in each boxplot represents the median. N represents the number of subjects.

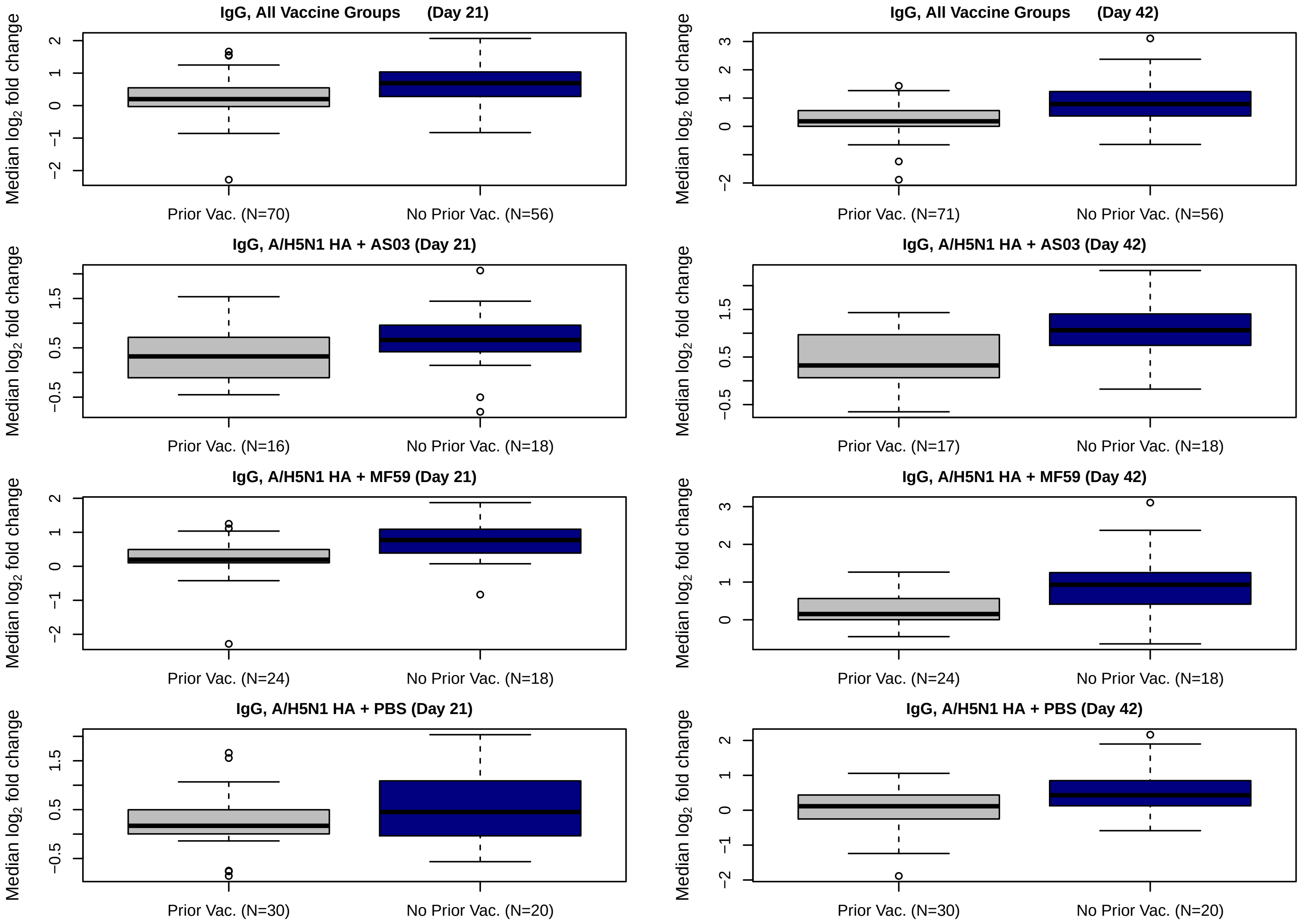

Supplementary Figure 3A Impact of prior seasonal influenza vaccination on H5 HA vaccination effects (Array 1, Experiment 1). Each boxplot summarizes the median log_2 fold change antibody responses across H5 HAs by vaccination status (subject receiving seasonal prior vaccinations within the past 2 years or those not receiving seasonal influenza vaccine within that time frame) and vaccine group for each antibody type (IgG and IgA) and post-vaccination day. Results within subjects for multiple H5 HA antigens were aggregated using the log_2 median fold change. The horizontal line in each boxplot represents the median. N represents the number of subjects.

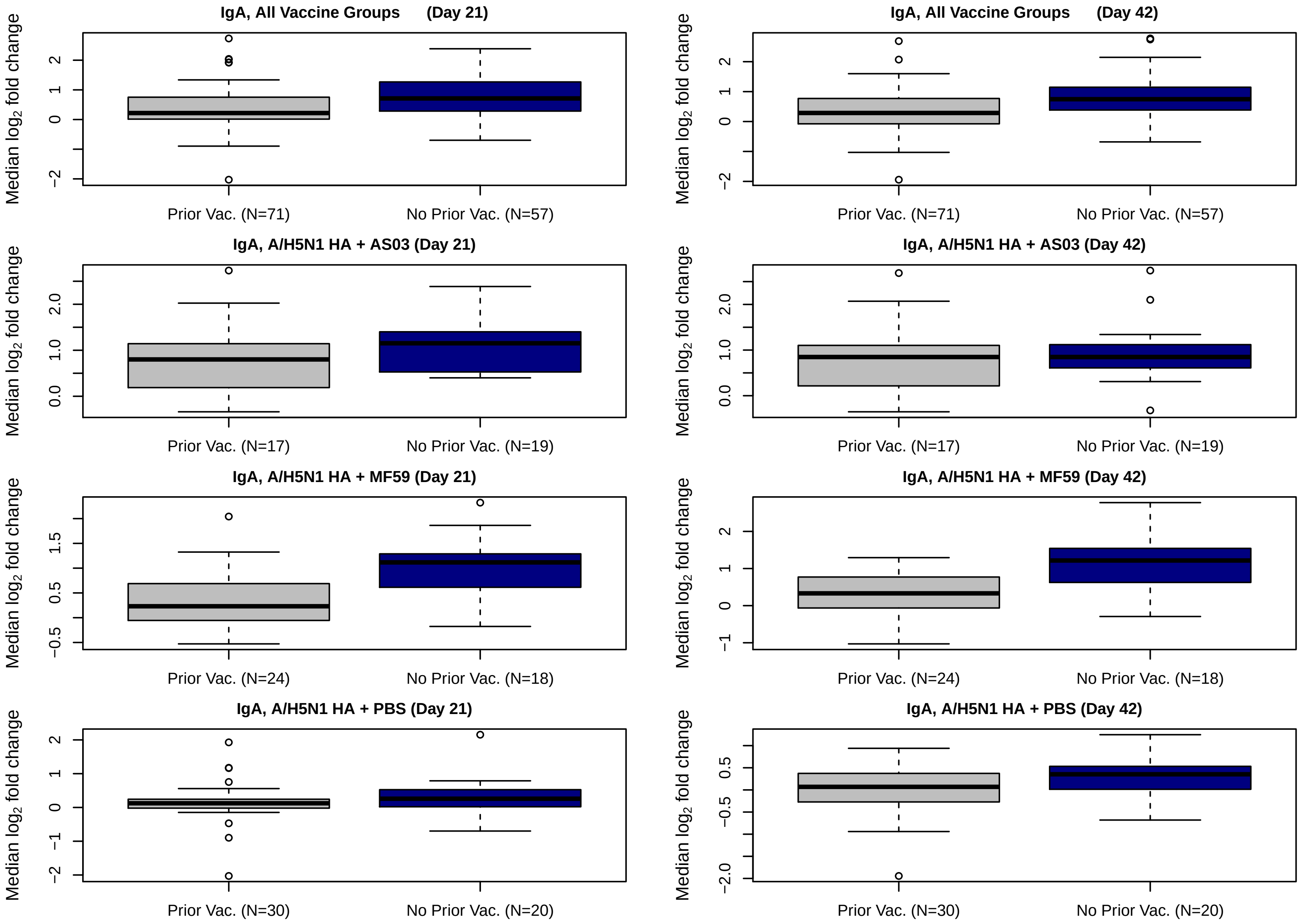

Supplementary Figure 3B Impact of prior seasonal influenza vaccination on H5N1 vaccination effects (Array 2, Experiment 2). Each boxplot summarizes the median log_2 fold change antibody responses across H5 HAs by vaccination status (subject receiving seasonal prior vaccinations within the past 2 years or those not receiving seasonal influenza vaccine within that time frame) and vaccine group for each antibody type (IgG and IgA) and post-vaccination day. Results within subjects for multiple H5 HA antigens were aggregated using the log_2 median fold change. The horizontal line in each boxplot represents the median. N represents the number of subjects.

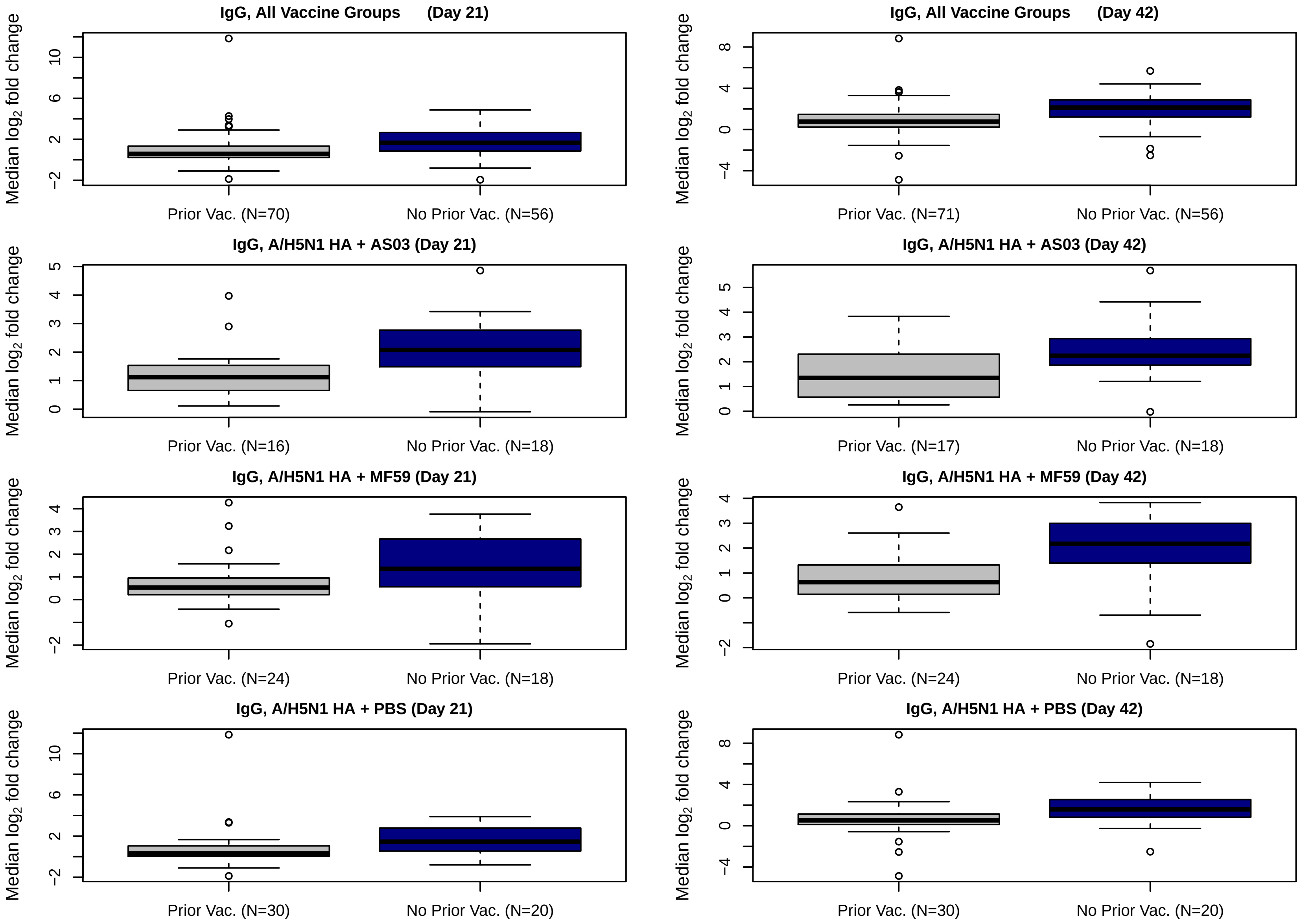

Supplementary Figure 3B Impact of prior seasonal influenza vaccination on H5N1 vaccination effects (Array 2, Experiment 2). Each boxplot summarizes the median log_2 fold change antibody responses across H5 HAs by vaccination status (subject receiving seasonal prior vaccinations within the past 2 years or those not receiving seasonal influenza vaccine within that time frame) and vaccine group for each antibody type (IgG and IgA) and post-vaccination day. Results within subjects for multiple H5 HA antigens were aggregated using the log_2 median fold change. The horizontal line in each boxplot represents the median. N represents the number of subjects.

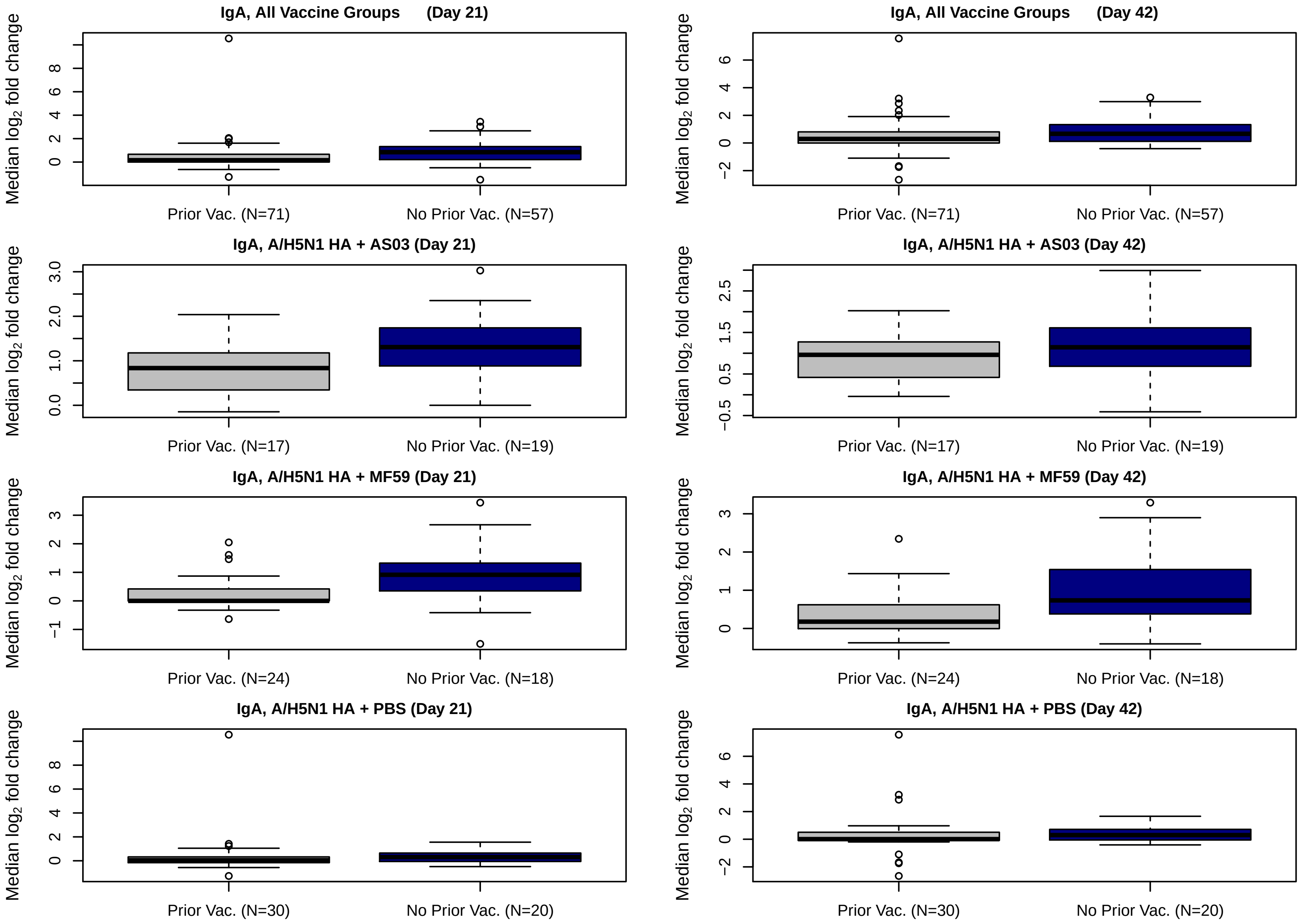

Supplementary Figure 4A Time trend plots of ranked IgG and IgA antibody responses against H5 HA1 and HA0 antigens based on geometric mean fluorescent intensity over time (1stprobing results). HA1: antibody responses against head H5 HA proteins, HA0: antibody responses against full length H5 HA proteins. GMI: Geometric mean fluorescent intensity.

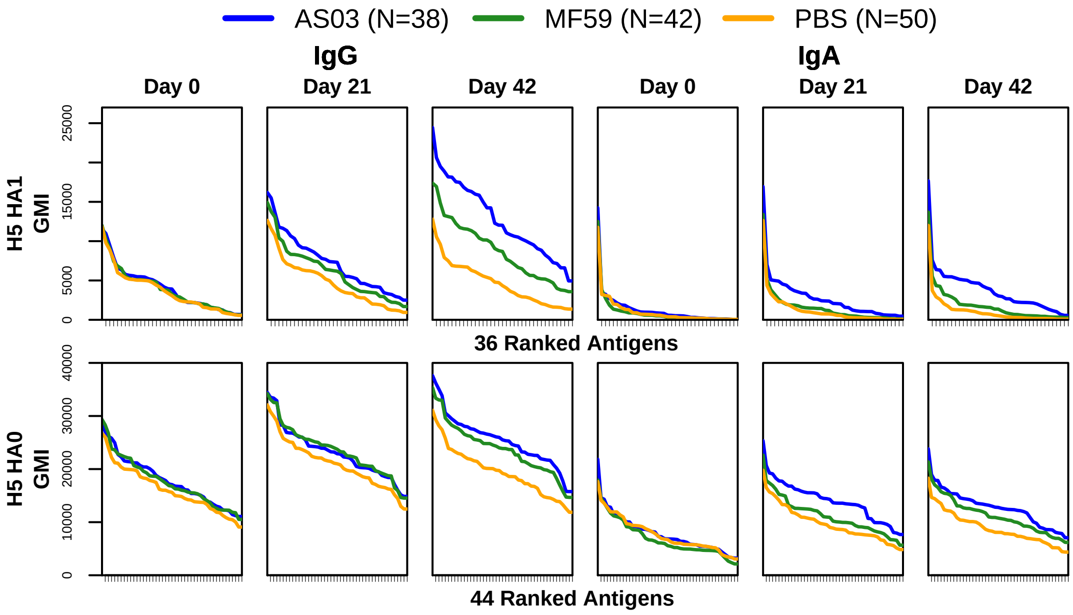

Supplementary Figure 4B Time trend plots of ranked IgG and IgA antibody responses against H5 HA1 and HA0 antigens based on geometric mean fluorescent intensity over time (2ndprobing results). HA1: antibody responses against head H5 HA proteins, HA0: antibody responses against full length H5 HA proteins. GMI: Geometric mean fluorescent intensity.

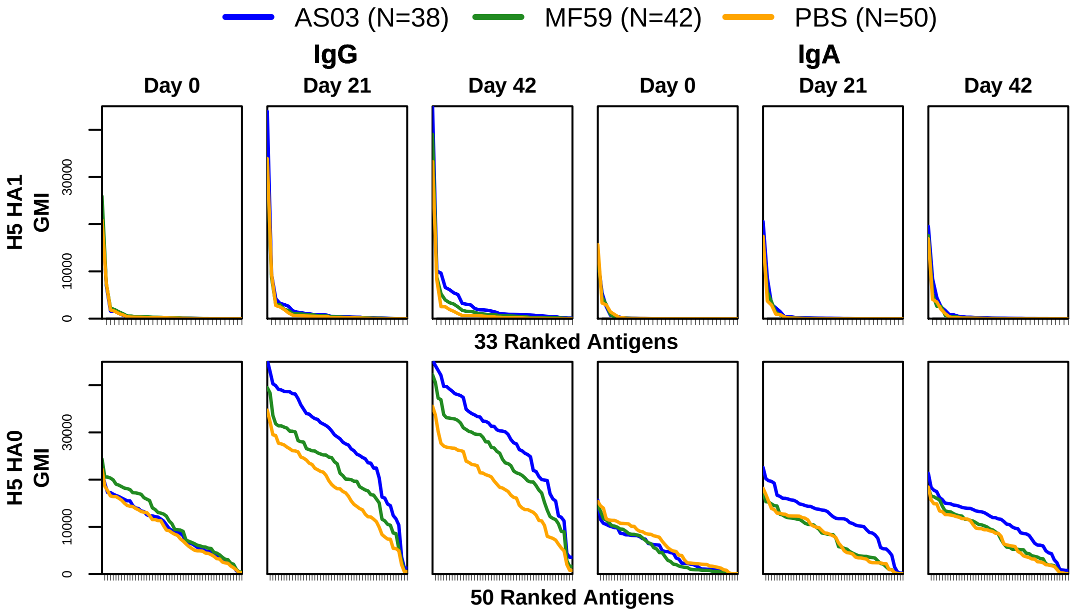

Supplementary Figure 4C Time trend plots of ranked IgG and IgA antibody responses against H5 HA1 and HA0 antigens based on geometric mean fluorescent intensity over time (1stprobing results). HA1: antibody responses against head H5 HA proteins, HA0: antibody responses against full length H5 HA proteins. GMI: Geometric mean fluorescent intensity.

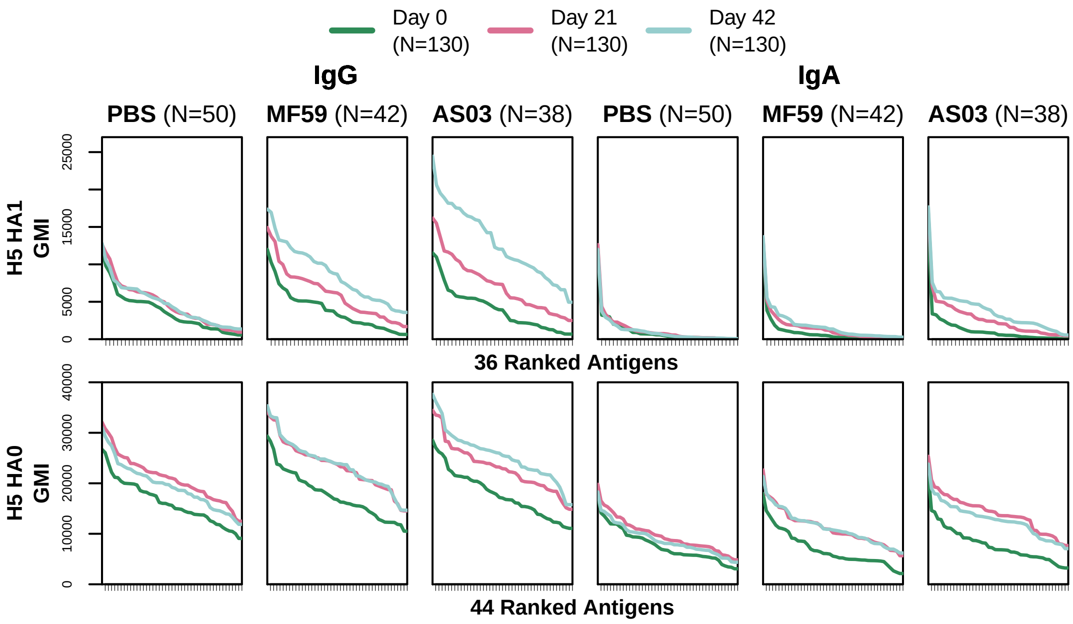

Supplementary Figure 4D Time trend plots of ranked IgG and IgA antibody responses against H5 HA1 and HA0 antigens based on geometric mean fluorescent intensity over time (2ndprobing results). HA1: antibody responses against head H5 HA proteins, HA0: antibody responses against full length H5 HA proteins. GMI: Geometric mean fluorescent intensity.

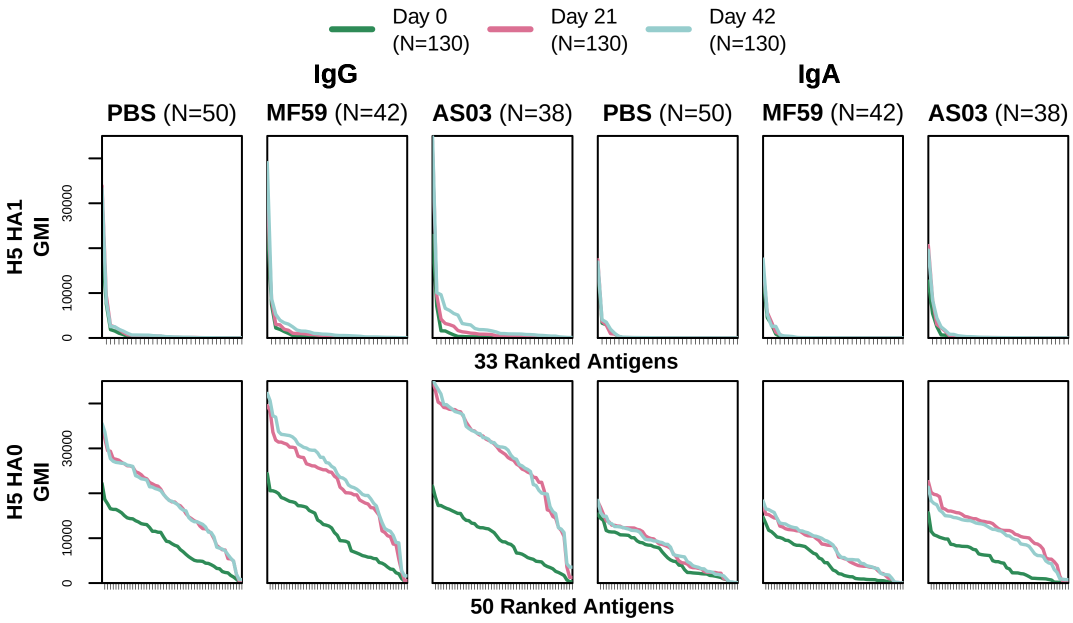

Supplementary Figure 5A IgG anti-stalk blocking effect by mini-HA concentration and HA subtype. Each trend line summarizes the geometric mean intensity by vaccine group, HA subtype across increasing mini-HA concentrations. Results within subjects for multiple antigens were aggregated using the geometric mean intensity.

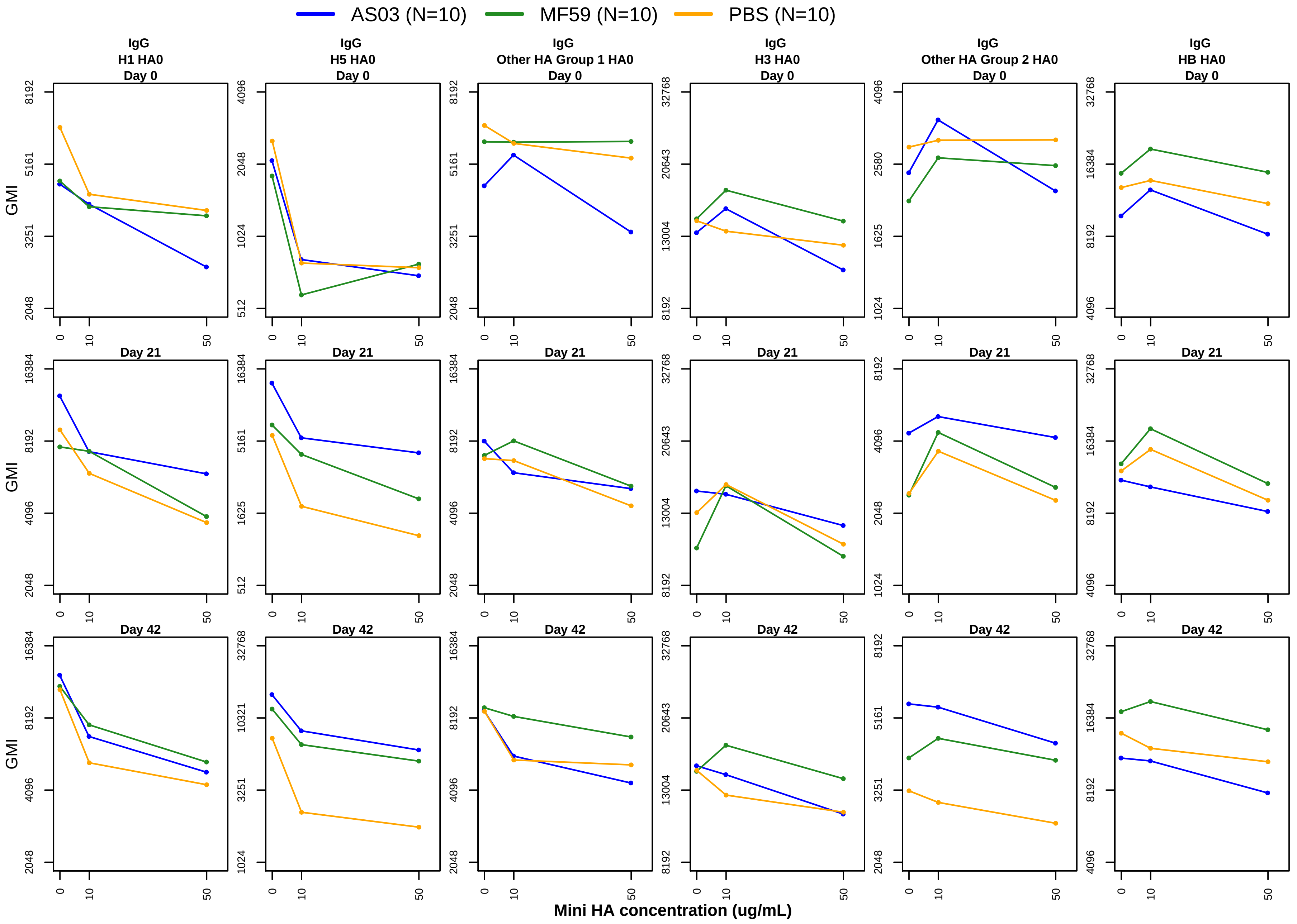

Supplementary Figure 5B IgA anti-stalk blocking effect by mini-HA concentration and HA subtype. Each trend line summarizes the geometric mean intensity by vaccine group, HA subtype across increasing mini-HA concentrations. Results within subjects for multiple antigens were aggregated using the geometric mean intensity.

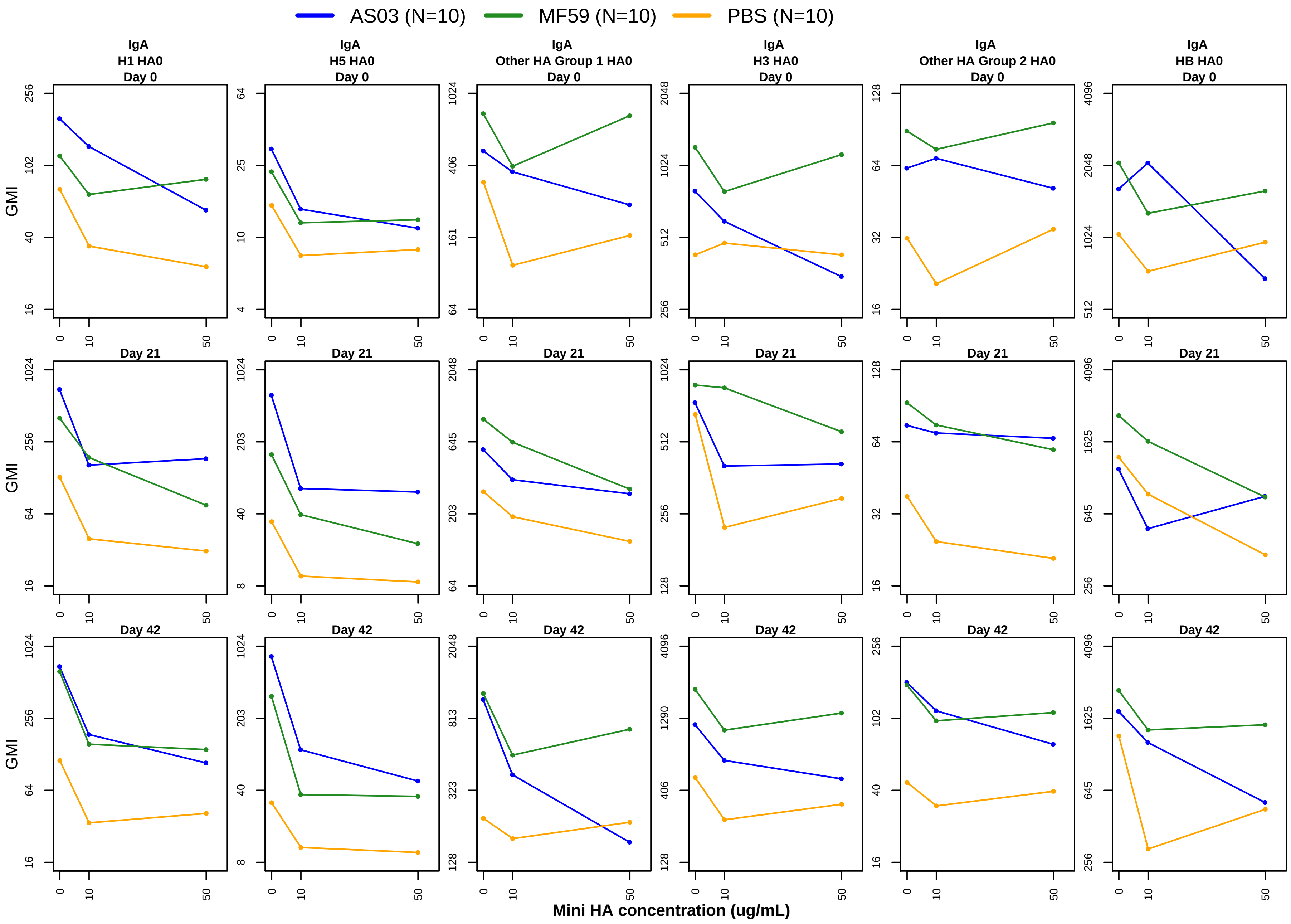

Supplementary Figure 6A Associations between dilution-based IgG titers based on microarray results and HAI and Microneutralization Titer. Results for 4 vaccine antigens are shown. The red line represents a locally weighted regression fit while the blue dashed line indicates a linear fit.

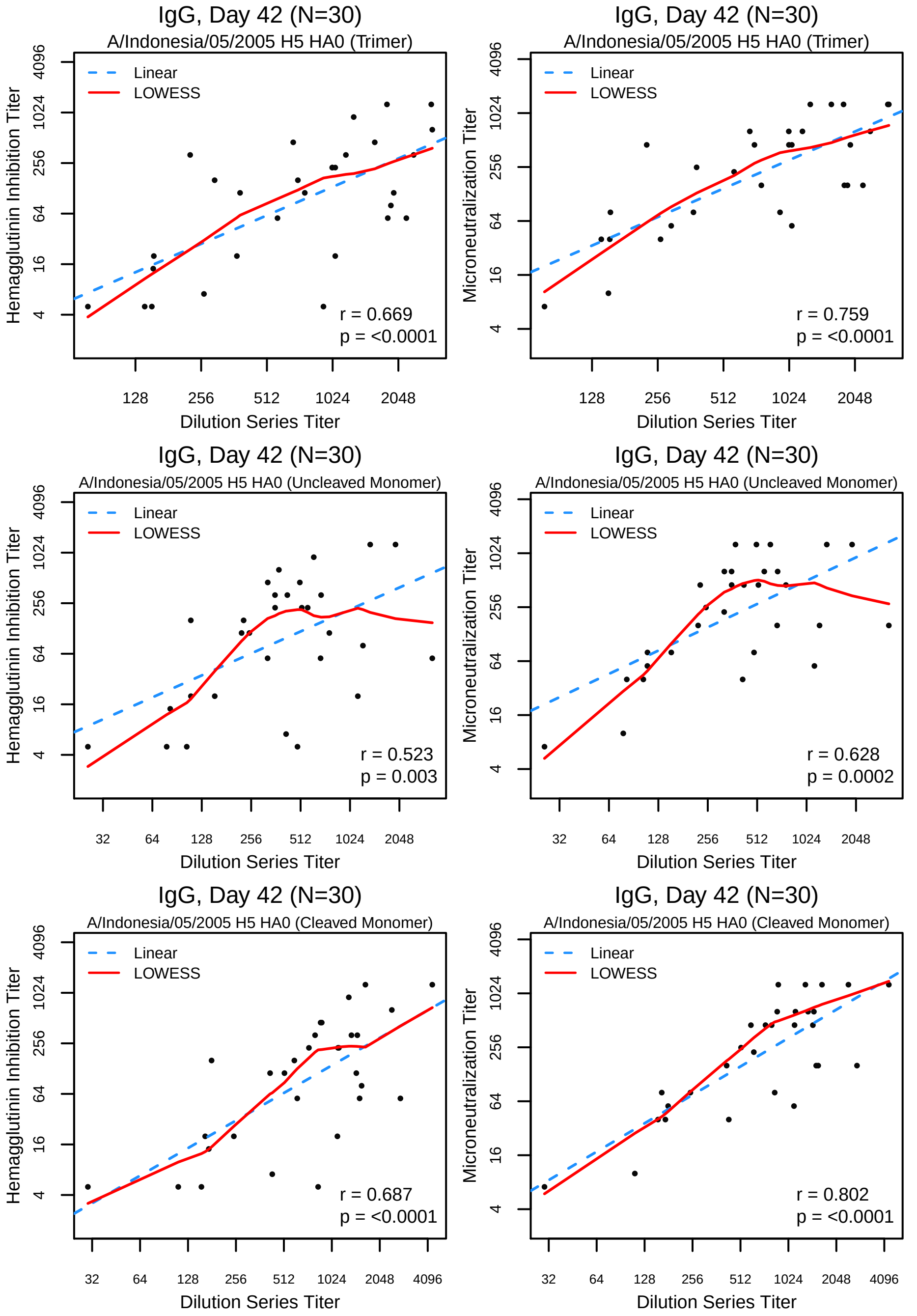

Supplementary Figure 6B Associations between dilution-based IgA titers based on microarray results and HAI and Microneutralization Titer. Results for 4 vaccine antigens are shown. The red line represents a locally weighted regression fit while the blue dashed line indicates a linear fit.

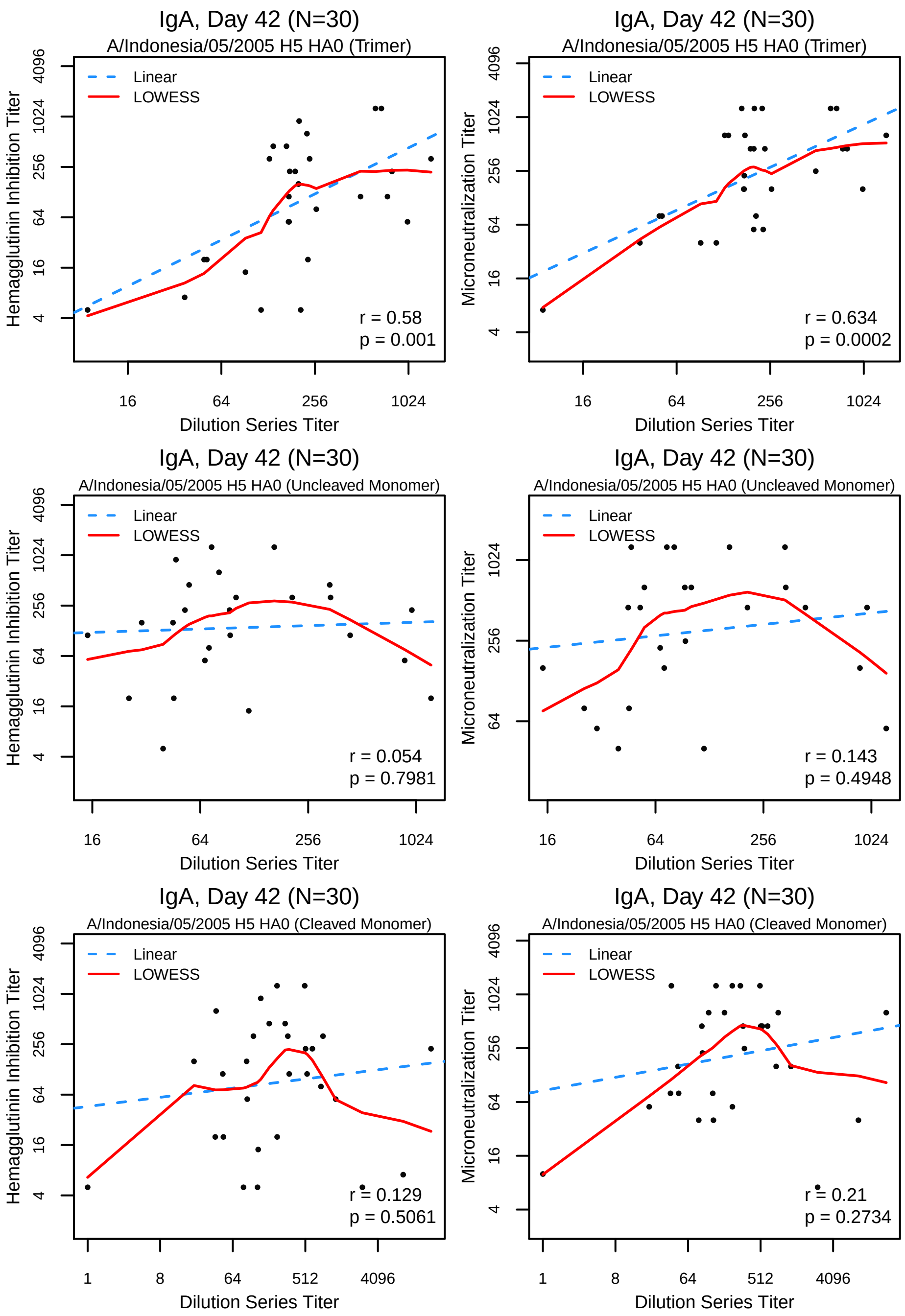

Supplementary Table 1A Demographics (Sex and Previous Vaccination)

		A/H5N1 HA + AS03 (N=38)		A/H5N1 HA + MF59 (N=42)		A/H5N1 HA + PBS (N=50)	
		**N**	**%**	**N**	**%**	**N**	**%**
Sex	Female	15	39.50%	13	31%	18	36%
	Male	23	60.50%	29	69%	32	64%
Previous vaccination	No	19	50%	18	42.90%	20	40%
	Yes	19	50%	24	57.10%	30	60%

Supplementary Table 1B Demographics (Age Distribution)

**Statistic**	**A/H5N1 HA + AS03 (N=38)**	**A/H5N1 HA + MF59 (N=42)**	**A/H5N1 HA + PBS (N=50)**
Mean	29.4	29.7	30.2
Standard Deviation	6.7	7.6	8.1
Median	27	28.5	28
Minimum	19	19	18
Maximum	46	49	47

Supplementary Table 2 List of Proteins on Array #1

Short ID	Long ID	Subtype	Mol ID1	Mol ID2	Is Group 1	Is Group 2	Source	Form1	Form2	GenBank	Expression System	Notes
10003-V04H2	A/VietNam/1203/2004	H5N1	HA	HA2	YES	NO	SinoBiological			AAW80717.1	Human cells	Fc tag (mouse)
10003-V06H1	A/VietNam/1203/2004	H5N1	HA	HA1	YES	NO	SinoBiological			AAW80717.1	Human cells	His Fc tag (mouse)
10003-V06H3	A/VietNam/1203/2004	H5N1	HA	HA0	YES	NO	SinoBiological	uncleaved		AAW80717.1	Human cells	His Fc tag (mouse)
11048-V06H1	A/Anhui/1/2005	H5N1	HA	HA0	YES	NO	SinoBiological	uncleaved		ABD28180.1	Human cells	His Fc tag (mouse)
11048-V08B	A/Anhui/1/2005 (B)	H5N1	HA	HA0	YES	NO	SinoBiological	uncleaved		ABD28180.1	Baculovirus	His tag
11048-V08H1	A/Anhui/1/2005 (H)	H5N1	HA	HA0	YES	NO	SinoBiological	uncleaved		ABD28180.1	Human cells	His tag
11048-V08H2	A/Anhui/1/2005	H5N1	HA	HA1	YES	NO	SinoBiological			ABD28180.1	Human cells	His tag
11048-V08H4	A/Anhui/1/2005	H5N1	HA	HA0	YES	NO	SinoBiological	cleaved	Native	ABD28180.1	Human cells	His tag
11048-VNAH2	A/Anhui/1/2005	H5N1	HA	HA1	YES	NO	SinoBiological			ABD 28180.1	Human cells	
11052-V08H	A/Brisbane/59/2007	H1N1	HA	HA0	YES	NO	SinoBiological	uncleaved	Native	ACA28844.1	Human cells	His tag
11052-V08H1	A/Brisbane/59/2007	H1N1	HA	HA1	YES	NO	SinoBiological			ACA28844.1	Human cells	His tag
11053-V04H2	B/Florida/4/2006	FluB	HA	HA2	NO	NO	SinoBiological			ACA33493.1	Human cells	Fc tag (mouse)
11053-V08H	B/Florida/4/2006	FluB	HA	HA0	NO	NO	SinoBiological	uncleaved	Native	ACA33493.1	Human cells	His tag
11053-V08H1	B/Florida/4/2006	FluB	HA	HA1	NO	NO	SinoBiological			ACA33493.1	Human cells	His tag
11055-V04H3	A/California/04/2009	H1N1	HA	HA2	YES	NO	SinoBiological			ACP41105.1	Human cells	Fc tag (mouse)
11055-V08B	A/California/04/2009 (B)	H1N1	HA	HA0	YES	NO	SinoBiological	uncleaved	Native	ACP41105.1	Baculovirus	His tag
11055-V08H	A/California/04/2009 (H)	H1N1	HA	HA0	YES	NO	SinoBiological	uncleaved	Native	ACP41105.1	Human cells	His tag
11055-V08H2	A/California/04/2009	H1N1	HA	HA0	YES	NO	SinoBiological	cleaved (partial)		ACP41105.1	Human cells	His tag
11055-V08H4	A/California/04/2009	H1N1	HA	HA1	YES	NO	SinoBiological			ACP41105.1	Human cells	His tag
11055-VNAB	A/California/04/2009	H1N1	HA	HA0	YES	NO	SinoBiological				Baculovirus	
11056-V08B	A/Brisbane/10/2007 (B)	H3N2	HA	HA0	NO	YES	SinoBiological			ABW23353.1	Baculovirus	His tag
11056-V08H	A/Brisbane/10/2007 (H)	H3N2	HA	HA0	NO	YES	SinoBiological	uncleaved	Native	ABW23353.1	Human cells	His tag
11056-V08H1	A/Brisbane/10/2007	H3N2	HA	HA1	NO	YES	SinoBiological			ABW23353.1	Human cells	His tag
11059-V08B1	A/bar-headed goose/Qinghai/14/2008 (B)	H5N1	HA	HA0	YES	NO	SinoBiological	uncleaved		ACL28277.1	Baculovirus	His tag
11059-V08H1	A/bar-headed goose/Qinghai/14/2008 (H)	H5N1	HA	HA0	YES	NO	SinoBiological	uncleaved		ACL28277.1	Human cells	His tag
11059-V08H2	A/bar-headed goose/Qinghai/14/2008	H5N1	HA	HA0	YES	NO	SinoBiological	cleaved	Native	ACL28277.1	Human cells	His tag
11060-V08H1	A/Indonesia/5/2005	H5N1	HA	HA0	YES	NO	SinoBiological	uncleaved		ABW06108.1	Human cells	His tag
11060-V08H2	A/Indonesia/5/2005	H5N1	HA	HA0	YES	NO	SinoBiological	cleaved	Native	ABW06108.1	Human cells	His tag
11061-V08H1	A/turkey/Turkey/1/2005	H5N1	HA	HA0	YES	NO	SinoBiological	uncleaved		ABD73284.1	Human cells	His tag
11061-V08H2	A/turkey/Turkey/1/2005	H5N1	HA	HA0	YES	NO	SinoBiological	cleaved	Native	ABD73284.1	Human cells	His tag
11062-V08H1	A/Vietnam/1194/2004	H5N1	HA	HA0	YES	NO	SinoBiological	uncleaved		AAT73273.1	Human cells	His tag
11062-V08H2	A/Vietnam/1194/2004	H5N1	HA	HA0	YES	NO	SinoBiological	cleaved	Native	AAT73273.1	Human cells	His tag
11068-V08H	A/Brevig Mission/1/1918	H1N1	HA	HA0	YES	NO	SinoBiological	uncleaved	Native	AAD17229.1	Human cells	His tag
11068-V08H1	A/Brevig Mission/1/1918	H1N1	HA	HA1	YES	NO	SinoBiological			AAD17229.1	Human cells	His tag
11082-V08B	A/Netherlands/219/03	H7N7	HA	HA0	NO	YES	SinoBiological		Native	AAR02640.1	Baculovirus	His tag
11082-V08H1	A/Netherlands/219/03	H7N7	HA	HA1	NO	YES	SinoBiological			AAR02640.1	Human cells	His tag
11085-V08B	A/California/07/2009 (B)	H1N1	HA	HA0	YES	NO	SinoBiological			ACP41953.1	Baculovirus	His tag
11085-V08H	A/California/07/2009 (H)	H1N1	HA	HA0	YES	NO	SinoBiological	uncleaved	2aadel	ACP44189.1	Human cells	His tag
11088-V08H	A/Japan/305/1957	H2N2	HA	HA0	YES	NO	SinoBiological	uncleaved		AAA43185.1	Human cells	His tag
11088-V08H1	A/Japan/305/1957	H2N2	HA	HA1	YES	NO	SinoBiological			AAO46269.1	Human cells	His tag
11212-V08B	A/chicken/Netherlands/1/03	H7N7	HA	HA0	NO	YES	SinoBiological		Native	AAR02639.1	Baculovirus	His tag
11212-V08H1	A/chicken/Netherlands/1/03	H7N7	HA	HA1	NO	YES	SinoBiological			AAR02639.1	Human cells	His tag
11229-V08H	A/Hong Kong/1073/99	H9N2	HA	HA0	YES	NO	SinoBiological		Native	NP$_8$59037.1	Human cells	His tag
11229-V08H1	A/HongKong/1073/99	H9N2	HA	HA1	YES	NO	SinoBiological			NP$_8$59037.1	Human cells	His tag
11683-V08H	A/New Caledonia/20/99	H1N1	HA	HA0	YES	NO	SinoBiological	uncleaved		AAP34324.1	Human cells	His tag
11683-V08H1	A/New Caledonia/20/99	H1N1	HA	HA1	YES	NO	SinoBiological			AAP34324.1	Human cells	His tag
11684-V08H	A/Puerto Rico/8/34	H1N1	HA	HA0	YES	NO	SinoBiological			ABD77675.1	Human cells	His tag
11684-V08H1	A/Puerto Rico/8/34	H1N1	HA	HA1	YES	NO	SinoBiological			ABD77675.1	Human cells	His tag
11685-V08H	A/duck/NZL/160/1976	H1N3	HA	HA0	YES	NO	SinoBiological	uncleaved		ABB20429.1	Human cells	His tag
11685-V08H1	A/duck/NZL/160/1976	H1N3	HA	HA1	YES	NO	SinoBiological			ABB20429.1	Human cells	His tag

Short ID	Long ID	Subtype	Mol ID1	Mol ID2	Is Group 1	Is Group 2	Source	Form1	Form2	GenBank	Expression System	Notes
11686-V08H1	A/chicken/Egypt/2253-1/2006	H5N1	HA	HA1	YES	NO	SinoBiological			ABG81039.1	Human cells	His tag
11687-V08H	A/Ohio/UR06-0091/2007	H1N1	HA	HA0	YES	NO	SinoBiological	uncleaved		ABW40422.1	Human cells	His tag
11687-V08H1	A/Ohio/UR06-0091/2007	H1N1	HA	HA1	YES	NO	SinoBiological			ABW40422.1	Human cells	His tag
11688-V08H	A/Canada/720/2005	H2N2	HA	HA0	YES	NO	SinoBiological	uncleaved		AAY28987.1	Human cells	His tag
11688-V08H1	A/Canada/720/2005	H2N2	HA	HA1	YES	NO	SinoBiological			AAY28987.1	Human cells	His tag
11689-V08H	A/Hong Kong/483/97	H5N1	HA	HA0	YES	NO	SinoBiological	uncleaved	Mut clv site	AAC32099.1	Human cells	His tag
11689-V08H1	A/Hong Kong/483/97	H5N1	HA	HA1	YES	NO	SinoBiological			AAC32099.1	Human cells	His tag
11690-V08H	A/goose/Guiyang/337/2006	H5N1	HA	HA0	YES	NO	SinoBiological	uncleaved	Mut clv site	ABJ96698.1	Human cells	His tag
11690-V08H1	A/goose/Guiyang/337/2006	H5N1	HA	HA1	YES	NO	SinoBiological			ABJ96698.1	Human cells	His tag
11692-V08B	A/WSN/1933 (B)	H1N1	HA	HA0	YES	NO	SinoBiological			ACF54598.1	Baculovirus	His tag
11692-V08H	A/WSN/1933 (H)	H1N1	HA	HA0	YES	NO	SinoBiological	uncleaved		ACF54598.1	Human cells	His tag
11692-V08H1	A/WSN/1933	H1N1	HA	HA1	YES	NO	SinoBiological			ACF54598.1	Human cells	His tag
11693-V08B	A/duck/Hong Kong/786/1979 (B)	H10N3	HA	HA0	NO	YES	SinoBiological			BAF46762.1	Baculovirus	His tag
11693-V08H	A/duck/Hong Kong/786/1979 (H)	H10N3	HA	HA0	NO	YES	SinoBiological	uncleaved		BAF46762.1	Human cells	His tag
11693-V08H1	A/duck/Hong Kong/786/1979	H10N3	HA	HA1	NO	YES	SinoBiological			BAF46762.1	Human cells	His tag
11694-V08H	A/Japanese white-eye/Hong Kong/1038/2006	H5N1	HA	HA0	YES	NO	SinoBiological	uncleaved	Mut clv site	ABJ96775.1	Human cells	His tag
11694-V08H1	A/Japanese white-eye/Hong Kong/1038/2006	H5N1	HA	HA1	YES	NO	SinoBiological			ABJ96775.1	Human cells	His tag
11696-V08H	A/duck/Hokkaido/167/2007	H5N3	HA	HA0	YES	NO	SinoBiological			BAG07130.2	Human cells	His tag
11696-V08H1	A/duck/Hokkaido/167/2007	H5N3	HA	HA1	YES	NO	SinoBiological			BAG07130.2	Human cells	His tag
11697-V08H	A/Egypt/2321-NAMRU3/2007	H5N1	HA	HA0	YES	NO	SinoBiological	uncleaved	Mut clv site	ABP96850.1	Human cells	His tag
11697-V08H1	A/Egypt/2321-NAMRU3/2007	H5N1	HA	HA1	YES	NO	SinoBiological			ABP96850.1	Human cells	His tag
11698-V08H	A/duck/Hunan/795/2002	H5N1	HA	HA0	YES	NO	SinoBiological	uncleaved	Mut clv site	ACA47835.1	Human cells	His tag
11698-V08H1	A/duck/Hunan/795/2002	H5N1	HA	HA1	YES	NO	SinoBiological			ACA47835.1	Human cells	His tag
11699-V08H	A/American green-winged teal/California/HKWF609/2007	H5N2	HA	HA0	YES	NO	SinoBiological	uncleaved		ACF47563.1	Human cells	His tag
11699-V08H1	A/American green-winged teal/California/HKWF609/2007	H5N2	HA	HA1	YES	NO	SinoBiological			ACF47563.1	Human cells	His tag
11700-V08H	A/Common magpie/Hong Kong/2256/2006	H5N1	HA	HA0	YES	NO	SinoBiological	uncleaved	Mut clv site	ABJ96777.1	Human cells	His tag
11700-V08H1	A/Common magpie/Hong Kong/2256/2006	H5N1	HA	HA1	YES	NO	SinoBiological			ABJ96777.1	Human cells	His tag
11701-V08H1	A/duck/Laos/3295/2006	H5N1	HA	HA1	YES	NO	SinoBiological			ABG67978.1	Human cells	His tag
11702-V08H	A/Egypt/N05056/2009	H5N1	HA	HA0	YES	NO	SinoBiological	uncleaved	Mut clv site	ACT15357.1	Human cells	His tag
11702-V08H1	A/Egypt/N05056/2009	H5N1	HA	HA1	YES	NO	SinoBiological			ACT15357.1	Human cells	His tag
11703-V08H	A/swine/Guangxi/13/2006	H1N2	HA	HA0	YES	NO	SinoBiological	uncleaved		ABQ42444.1	Human cells	His tag
11703-V08H1	A/swine/Guangxi/13/2006	H1N2	HA	HA1	YES	NO	SinoBiological			ABQ42444.1	Human cells	His tag
11704-V08H	A/mallard/Alberta/294/1977	H11N9	HA	HA0	YES	NO	SinoBiological	uncleaved		ABB87228.1	Human cells	His tag
11704-V08H1	A/mallard/Alberta/294/1977	H11N9	HA	HA1	YES	NO	SinoBiological			ABB87228.1	Human cells	His tag
11705-V08H	A/duck/Yangzhou/906/2002	H11N2	HA	HA0	YES	NO	F. Krammer Laboratory	uncleaved		AAY85533.1	Human cells	His tag
11705-V08H1	A/duck/Yangzhou/906/2002	H11N2	HA	HA1	YES	NO	SinoBiological			AAY85533.1	Human cells	His tag
11706-V08H	A/Swine/Ontario/01911-1/99	H4N6	HA	HA0	NO	YES	SinoBiological	uncleaved		AAG17429.1	Human cells	His tag
11706-V08H1	A/Swine/Ontario/01911-1/99	H4N6	HA	HA1	NO	YES	SinoBiological			AAG17429.1	Human cells	His tag
11707-V08H	A/Aichi/2/1968	H3N2	HA	HA0	NO	YES	SinoBiological	uncleaved		AAA43178.1	Human cells	His tag
11707-V08H1	A/Aichi/2/1968	H3N2	HA	HA1	NO	YES	SinoBiological			AAA43178.1	Human cells	His tag
11708-V08H	A/Solomon Islands/3/2006	H1N1	HA	HA0	YES	NO	SinoBiological	uncleaved		ABU99109.1	Human cells	His tag
11708-V08H1	A/Solomon Islands/3/2006	H1N1	HA	HA1	YES	NO	SinoBiological			ABU99109.1	Human cells	His tag
11709-V08H	A/whooper swan/Mongolia/244/2005	H5N1	HA	HA0	YES	NO	SinoBiological	uncleaved	Mut clv site	ACZ36881.1	Human cells	His tag
11709-V08H1	A/whooper swan/Mongolia/244/2005	H5N1	HA	HA1	YES	NO	SinoBiological			ACZ36881.1	Human cells	His tag
11710-V08B	A/Cambodia/R0405050/2007 (B)	H5N1	HA	HA0	YES	NO	SinoBiological				Baculovirus	His tag
11710-V08H	A/Cambodia/R0405050/2007 (H)	H5N1	HA	HA0	YES	NO	SinoBiological	uncleaved	Mut clv site	ACI06178.1	Human cells	His tag
11710-V08H1	A/Cambodia/R0405050/2007	H5N1	HA	HA1	YES	NO	SinoBiological			ACI06178.1	Human cells	His tag
11711-V08H	A/black-headed gull/Sweden/5/99	H16N3	HA	HA0	YES	NO	SinoBiological	uncleaved		AAV91217.1	Human cells	His tag
11711-V08H1	A/black-headed gull/Sweden/5/99	H16N3	HA	HA1	YES	NO	SinoBiological			AAV91217.1	Human cells	His tag

Short ID	Long ID	Subtype	Mol ID1	Mol ID2	Is Group 1	Is Group 2	Source	Form1	Form2	GenBank	Expression System	Notes
11712-V08B	A/chicken/India/NIV33487/06 (B)	H5N1	HA	HA0	YES	NO	SinoBiological			ABQ45850.1	Baculovirus	His tag
11712-V08H	A/chicken/India/NIV33487/06 (H)	H5N1	HA	HA0	YES	NO	SinoBiological	uncleaved	Mut clv site	ABQ45850.1	Human cells	His tag
11712-V08H1	A/chicken/India/NIV33487/06	H5N1	HA	HA1	YES	NO	SinoBiological			ABQ45850.1	Human cells	His tag
11713-V08H	A/Hong kong/213/2003	H5N1	HA	HA0	YES	NO	SinoBiological	uncleaved	Mut clv site	ABP51975.1	Human cells	His tag
11713-V08H1	A/Hong kong/213/2003	H5N1	HA	HA1	YES	NO	SinoBiological		1 aa mut	ABP51975.1	Human cells	His tag
11714-V08H	A/mallard/Ohio/657/2002	H4N6	HA	HA0	NO	YES	SinoBiological	uncleaved		ABI47995.1	Human cells	His tag
11714-V08H1	A/mallard/Ohio/657/2002	H4N6	HA	HA1	NO	YES	SinoBiological			ABI47995.1	Human cells	His tag
11715-V08H	A/Wyoming/03/2003	H3N2	HA	HA0	NO	YES	SinoBiological	uncleaved		ABX10525.1	Human cells	His tag
11715-V08H1	A/Wyoming/03/2003	H3N2	HA	HA1	NO	YES	SinoBiological			ABX10525.1	Human cells	His tag
11716-V08H	B/Malaysia/2506/2004	FluB	HA	HA0	NO	NO	SinoBiological	uncleaved		ACO05957.1	Human cells	His tag
11716-V08H1	B/Malaysia/2506/2004	FluB	HA	HA1	NO	NO	SinoBiological			ACO05957.1	Human cells	His tag
11717-V08H	A/duck/NY/191255-59/2002	H5N8	HA	HA0	YES	NO	SinoBiological	uncleaved		AAP72011.1	Human cells	His tag
11717-V08H1	A/duck/NY/191255-59/2002	H5N8	HA	HA1	YES	NO	SinoBiological			AAP72011.1	Human cells	His tag
11718-V08H	A/green-winged teal/ALB/199/1991	H12N5	HA	HA0	YES	NO	SinoBiological	uncleaved		ABB88110.1	Human cells	His tag
11718-V08H1	A/green-winged teal/ALB/199/1991	H12N5	HA	HA1	YES	NO	SinoBiological			ABB88110.1	Human cells	His tag
11719-V08H	A/Guinea fowl/Hong Kong/WF10/99	H9N2	HA	HA0	YES	NO	SinoBiological	uncleaved		AAO46082.1	Human cells	His tag
11719-V08H1	A/Guinea fowl/Hong Kong/WF10/99	H9N2	HA	HA1	YES	NO	SinoBiological			AAO46082.1	Human cells	His tag
11720-V08H	A/duck/AUS/341/1983	H15N8	HA	HA0	NO	YES	SinoBiological	uncleaved		ABB88132.1	Human cells	His tag
11720-V08H1	A/duck/AUS/341/1983	H15N8	HA	HA1	NO	YES	SinoBiological			ABB88132.1	Human cells	His tag
11721-V08B	A/black-headed gull/Netherlands/1/00	H13N8	HA	HA0	YES	NO	SinoBiological			AAV91212.1	Baculovirus	His tag
11721-V08H	A/black-headed gull/Netherlands/1/00	H13N8	HA	HA0	YES	NO	SinoBiological	uncleaved		AAV91212.1	Human cells	His tag
11721-V08H1	A/black-headed gull/Netherlands/1/00	H13N8	HA	HA1	YES	NO	SinoBiological			AAV91212.1	Human cells	His tag
11722-V08B	A/pintail duck/Alberta/114/1979 (B)	H8N4	HA	HA0	YES	NO	SinoBiological				Baculovirus	His tag
11722-V08H	A/pintail duck/Alberta/114/1979 (H)	H8N4	HA	HA0	YES	NO	SinoBiological	uncleaved		ABB87729.1	Human cells	His tag
11722-V08H1	A/pintail duck/Alberta/114/1979	H8N4	HA	HA1	YES	NO	SinoBiological			ABB87729.1	Human cells	His tag
11723-V08H	A/northern shoveler/California/HKWF115/2007	H6N1	HA	HA0	YES	NO	SinoBiological	uncleaved		ACE81692.1	Human cells	His tag
11723-V08H1	A/northern shoveler/California/HKWF115/2007	H6N1	HA	HA1	YES	NO	SinoBiological			ACE81692.1	Human cells	His tag
11972-V08B	A/Wisconsin/67/X-161/2005 (B)	H3N2	HA	HA0	NO	YES	SinoBiological			ACF41911.1	Baculovirus	His tag
11972-V08H	A/Wisconsin/67/X-161/2005 (H)	H3N2	HA	HA0	NO	YES	SinoBiological	uncleaved		ABO37609.1	Human cells	His tag
11972-V08H1	A/Wisconsin/67/X-161/2005	H3N2	HA	HA1	NO	YES	SinoBiological			ABO37609.1	Human cells	His tag
40001-V08H	A/Duck/Hong Kong/p46/97	H5N1	HA	HA0	YES	NO	SinoBiological	uncleaved	Mut clv site	AAF02306.1	Human cells	His tag
40001-V08H1	A/Duck/Hong Kong/p46/97	H5N1	HA	HA1	YES	NO	SinoBiological			AAF02306.1	Human cells	His tag
40003-V08B	A/Duck/HongKong/448/78	H9N2	HA	HA0	YES	NO	SinoBiological			AAO46079.1	Baculovirus	His tag
40004-V08H	A/Xinjiang/1/2006	H5N1	HA	HA0	YES	NO	SinoBiological	uncleaved	Mut clv site	ACJ68614.1	Human cells	His tag
40004-V08H1	A/Xinjiang/1/2006	H5N1	HA	HA1	YES	NO	SinoBiological			ACJ68614.1	Human cells	His tag
40005-V08H	A/England/195/2009	H1N1	HA	HA0	YES	NO	SinoBiological	uncleaved		ACR15621.1	Human cells	His tag
40005-V08H1	A/England/195/2009	H1N1	HA	HA1	YES	NO	SinoBiological			ACR15621.1	Human cells	His tag
40006-V08H	A/Texas/05/2009	H1N1	HA	HA0	YES	NO	SinoBiological	uncleaved		ACP41934.1	Human cells	His tag
40006-V08H1	A/Texas/05/2009	H1N1	HA	HA1	YES	NO	SinoBiological			ACP41934.1	Human cells	His tag
40007-V08H	A/Ohio/07/2009	H1N1	HA	HA0	YES	NO	SinoBiological	uncleaved		ACQ63286.1	Human cells	His tag
40007-V08H1	A/Ohio/07/2009	H1N1	HA	HA1	YES	NO	SinoBiological			ACR38870.1	Human cells	His tag
40008-V08B	A/mallard duck/Alberta/299/1977 (B)	H4N4	HA	HA0	NO	YES	SinoBiological			ABB87495.1	Baculovirus	His tag
40008-V08H	A/mallard duck/Alberta/299/1977 (H)	H4N4	HA	HA0	NO	YES	SinoBiological				Human cells	His tag
40008-V08H1	A/mallard duck/Alberta/299/1977	H4N4	HA	HA1	NO	YES	SinoBiological			Q0A4G1	Human cells	His tag
40009-V08B	A/New York/18/2009 (B)	H1N1	HA	HA0	YES	NO	SinoBiological			ACR08536.1	Baculovirus	His tag
40009-V08H	A/New York/18/2009 (H)	H1N1	HA	HA0	YES	NO	SinoBiological	uncleaved		ACU13097.1	Human cells	His tag
40009-V08H1	A/New York/18/2009	H1N1	HA	HA1	YES	NO	SinoBiological			ACU13097.1	Human cells	His tag
40014-V08H1	A/ostrich/South Africa/AI1091/2006	H5N2	HA	HA1	YES	NO	SinoBiological			ABQ24010.1	Human cells	His tag
40015-V08B	A/Hubei/1/2010 (B)	H5N1	HA	HA0	YES	NO	SinoBiological			AEO89181.1	Baculovirus	His tag
40015-V08H	A/Hubei/1/2010 (H)	H5N1	HA	HA0	YES	NO	SinoBiological	cleaved			Human cells	His tag
40015-V08H1	A/Hubei/2011	H5N1	HA	HA1	YES	NO	SinoBiological				Human cells	His tag
40016-V08H	B/Brisbane/60/2008	FluB	HA	HA0	NO	NO	SinoBiological			ACN29380.1	Human cells	His tag
40016-V08H1	B/Brisbane/60/2008	FluB	HA	HA1	NO	NO	SinoBiological			ACN29383.1	Human cells	His tag
40022-V08H1	A/Vietnam/UT31413II/2008	H5N1	HA	HA1	YES	NO	SinoBiological			ADF83651.1	Human cells	His tag

Short ID	Long ID	Subtype	Mol ID1	Mol ID2	Is Group 1	Is Group 2	Source	Form1	Form2	GenBank	Expression System	Notes
40024-V08B	A/Goose/Guangdong/1/96	H5N1	HA	HA0	YES	NO	SinoBiological			YP$_3$08669.1	Baculovirus	His tag
40025-V08H	A/chicken/Alabama/1/1975	H4N8	HA	HA0	NO	YES	SinoBiological				Human cells	His tag
40025-V08H1	A/chicken/Alabama/1/1975	H4N8	HA	HA1	NO	YES	SinoBiological			P19695.1	Human cells	His tag
40026-V08H	A/Cambodia/S1211394/2008	H5N1	HA	HA0	YES	NO	SinoBiological			ADM95445.1	Human cells	His tag
40026-V08H1	A/Cambodia/S1211394/2008	H5N1	HA	HA1	YES	NO	SinoBiological			ADM95445.1	Human cells	His tag
40027-V08B	A/chicken/Hong Kong/17/1977 (B)	H6N4	HA	HA0	YES	NO	SinoBiological			CAC84244.1	Baculovirus	His tag
40027-V08H	A/chicken/HongKong/17/77 (H)	H6N4	HA	HA0	YES	NO	SinoBiological			CAC84244.1	Human cells	His tag
40027-V08H1	A/chicken/Hong Kong/17/77	H6N4	HA	HA1	YES	NO	SinoBiological			CAC84244.1	Human cells	His tag
40028-V08B	A/duck/Hong Kong/562/1979 (B)	H10N9	HA	HA0	NO	YES	SinoBiological			ABI84469.1	Baculovirus	His tag
40028-V08H	A/duck/Hong Kong/562/1979 (H)	H10N9	HA	HA0	NO	YES	SinoBiological			ABI84469.1	Human cells	His tag
40028-V08H1	A/duck/Hong Kong/562/1979	H10N9	HA	HA1	NO	YES	SinoBiological			ABI84469.1	Human cells	His tag
40029-V08B	A/mallard duck/Alberta/342/1983 (B)	H12N1	HA	HA0	YES	NO	SinoBiological			ABB88099.1	Baculovirus	His tag
40029-V08H	A/mallard duck/Alberta/342/1983 (H)	H12N1	HA	HA0	YES	NO	SinoBiological			ABB88099.1	Human cells	His tag
40029-V08H1	A/mallard duck/Alberta/342/1983	H12N1	HA	HA1	YES	NO	SinoBiological			ABB88099.1	Human cells	His tag
40035-V08H	A/Beijing/22808/2009	H1N1	HA	HA0	YES	NO	SinoBiological			ADD64203.1	Human cells	His tag
40035-V08H1	A/Beijing/22808/2009	H1N1	HA	HA1	YES	NO	SinoBiological			ABI84516.1	Human cells	His tag
40036-V08H1	A/Chicken/Hong Kong/G9/97	H9N2	HA	HA1	YES	NO	SinoBiological			AAF00701.1	Human cells	His tag
40043-V08H	A/Perth/16/2009	H3N2	HA	HA0	NO	YES	SinoBiological			ACS71642.1	Human cells	His tag
40043-V08H1	A/Perth/16/2009	H3N2	HA	HA1	NO	YES	SinoBiological			ACS71642.1	Human cells	His tag
40044-V08H	A/common magpie/Hong Kong/5052/2007	H5N1	HA	HA0	YES	NO	SinoBiological			ACJ26242.1	Human cells	His tag
40044-V08H1	A/common magpie/Hong Kong/5052/2007	H5N1	HA	HA1	YES	NO	SinoBiological			ACJ26242.1	Human cells	His tag
40049-V08H1	A/Egypt/3300-NAMRU3/2008	H5N1	HA	HA1	YES	NO	SinoBiological				Human cells	His tag
40058-V08B	A/Victoria/210/2009	H3N2	HA	HA0	NO	YES	SinoBiological			ADI52838.1	Baculovirus	His tag
40058-V08H1	A/reassortant/IVR-155(Victoria/210/2009 x Puerto Rico/8/1934)	H3N2	HA	HA1	NO	YES	SinoBiological				Human cells	His tag
40059-V08H	A/X-31	H3N2	HA	HA0	NO	YES	SinoBiological			P03438	Human cells	His tag
40060-V08H1	A/Hubei/1/2010	H5N1	HA	HA1	YES	NO	SinoBiological			AEO89181.1	Human cells	His tag
40064-V07H	A/Thailand/1(KAN-1)/2004	H5N1			NO	NO	SinoBiological				Human cells	His tag
40064-V07H-B	A/Thailand/1(KAN-1)/2004	H5N1			NO	NO	SinoBiological				Baculovirus	His tag + Bi-otin
40065-V08H1	A/Thailand/1(KAN-1)/2004	H5N1	HA	HA1	YES	NO	SinoBiological				Human cells	His tag
40088-V08H1	A/chicken/Yamaguchi/7/2004	H5N1	HA	HA1	YES	NO	SinoBiological			BAD89305.1	Human cells	His tag
40090-V08B	A/New York/1/1918	H1N1	HA	HA0	YES	NO	SinoBiological			AAD17219.1	Baculovirus	His tag
40090-V08H1	A/New York/1/1918	H1N1	HA	HA1	YES	NO	SinoBiological			AAD17219.1	Human cells	His tag
40101-V08H1	A/Memphis/1/68	H3N2	HA	HA1	NO	YES	SinoBiological			ABB54514.1	Human cells	His tag
40103-V08B	A/Anhui/1/2013 (B)	H7N9	HA	HA0	NO	YES	SinoBiological			EPI439507	Baculovirus	His tag
40103-V08H	A/Anhui/1/2013 (H)	H7N9	HA	HA0	NO	YES	SinoBiological			EPI439507	Human cells	His tag
40103-V08H1	A/Anhui/1/2013	H7N9	HA	HA1	NO	YES	SinoBiological			AGJ51953.1	Human cells	His tag
40103-V08H4	A/Anhui/1/2013	H7N9	HA	HA0	NO	YES	SinoBiological	cleaved		EPI439507	Human cells	His tag
40104-V08B	A/Shanghai/1/2013 (B)	H7N9	HA	HA0	NO	YES	SinoBiological				Baculovirus	His tag
40104-V08B1	A/Shanghai/1/2013 (B)	H7N9	HA	HA1	NO	YES	SinoBiological				Baculovirus	His tag
40104-V08H	A/Shanghai/1/2013 (Hi)	H7N9	HA	HA0	NO	YES	SinoBiological				Human cells	His tag
40104-V08H1	A/Shanghai/1/2013 (H)	H7N9	HA	HA1	NO	YES	SinoBiological				Human cells	His tag
40104-V08H4	A/Shanghai/1/2013 (Hii)	H7N9	HA	HA0	NO	YES	SinoBiological	cleaved			Human cells	His tag
40105-V08B	A/Hangzhou/1/2013 (B)	H7N9	HA	HA0	NO	YES	SinoBiological			AGI60301.1	Baculovirus	His tag
40105-V08H	A/Hangzhou/1/2013 (H)	H7N9	HA	HA0	NO	YES	SinoBiological			AGI60301.1	Human cells	His tag
40105-V08H1	A/Hangzhou/1/2013	H7N9	HA	HA1	NO	YES	SinoBiological			AGI60301.1	Human cells	His tag
40106-V08B	A/Pigeon/Shanghai/S1069/2013 (B)	H7N9	HA	HA0	NO	YES	SinoBiological				Baculovirus	His tag
40106-V08B1	A/Pigeon/Shanghai/S1069/2013 (B)	H7N9	HA	HA1	NO	YES	SinoBiological				Baculovirus	His tag
40106-V08H	A/Pigeon/Shanghai/S1069/2013 (H)	H7N9	HA	HA0	NO	YES	SinoBiological				Human cells	His tag
40109-V07H	A/Shanghai/1/2013	H7N9			NO	NO	SinoBiological				Human cells	His tag
40109-VNAHC	A/Shanghai/1/2013	H7N9			NO	NO	SinoBiological				Human cells	Active (bio-activity tested)
40111-V08B	A/Shanghai/2/2013	H7N9	NP	NP	NO	NO	SinoBiological			AGL44439.1	Baculovirus	His tag

Short ID	Long ID	Subtype	Mol ID1	Mol ID2	Is Group 1	Is Group 2	Source	Form1	Form2	GenBank	Expression System	Notes
40116-V08B	A/Hong Kong/1/1968	H3N2	HA	HA0	NO	YES	SinoBiological			Q91MA7	Baculovirus	His tag
40116-V08H1	A/Hong Kong/1/1968	H3N2	HA	HA1	NO	YES	SinoBiological			AAK51718.1	Human cells	His tag
40117-V08B	A/bar-headed goose/Qinghai/1A/2005	H5N1	HA	HA0	YES	NO	SinoBiological			ABF93441.1	Baculovirus	His tag
40117-V08H1	A/bar-headed goose/Qinghai/1A/2005	H5N1	HA	HA1	YES	NO	SinoBiological			ABF93441.1	Human cells	His tag
40118-V08B	A/California/7/2004	H3N2	HA	HA0	NO	YES	SinoBiological			ABW80975.1	Baculovirus	His tag
40118-V08H1	A/California/7/2004	H3N2	HA	HA1	NO	YES	SinoBiological			ABW80975.1	Human cells	His tag
40119-V08B	A/Guiyang/1/1957	H2N2	HA	HA0	YES	NO	SinoBiological			ACD85231.1	Baculovirus	His tag
40119-V08H1	A/Guiyang/1/1957	H2N2	HA	HA1	YES	NO	SinoBiological			ACD85231.1	Human cells	His tag
40120-V08B	A/Fujian/411/2002	H3N2	HA	HA0	NO	YES	SinoBiological			AFG72823.1	Baculovirus	His tag
40123-V08B	A/Hangzhou/3/2013	H7N9	HA	HA0	NO	YES	SinoBiological			EPI442713	Baculovirus	His tag
40125-V08B	A/Zhejiang/1/2013	H7N9	HA	HA0	NO	YES	SinoBiological			EPI443034	Baculovirus	His tag
40126-V08B	A/Shanghai/4664T/2013	H7N9	HA	HA0	NO	YES	SinoBiological			AGI60292.1	Baculovirus	His tag
40128-V08B	A/turkey/Italy/214845/2002	H7N3	HA	HA0	NO	YES	SinoBiological			CAF33017.1	Baculovirus	His tag
40128-V08H1	A/turkey/Italy/214845/2002	H7N3	HA	HA1	NO	YES	SinoBiological			CAF33017.1	Human cells	His tag
40129-V08H1	A/chicken/SK/HR-00011/2007	H7N3	HA	HA1	NO	YES	SinoBiological			ACA25329.1	Human cells	His tag
40131-V08B	A/Taiwan/01/1986	H1N1	HA	HA0	YES	NO	SinoBiological			ABF21274.1	Baculovirus	His tag
40132-V08H1	A/Texas/36/1991	H1N1	HA	HA1	YES	NO	SinoBiological			ACF41933.1	Human cells	His tag
40133-V08B	A/Beijing/262/1995	H1N1	HA	HA0	YES	NO	SinoBiological			ACF41867.1	Baculovirus	His tag
40134-V08B	A/USSR/90/1977	H1N1	HA	HA0	YES	NO	SinoBiological			P03453.2	Baculovirus	His tag
40134-V08H1	A/USSR/90/1977	H1N1	HA	HA1	YES	NO	SinoBiological			P03453	Human cells	His tag
40135-V08H1	A/Egyptian goose/South Africa/AI1448/2007	H1N8	HA	HA1	YES	NO	SinoBiological				Human cells	His tag
40136-V08B	A/mallard/Ohio/265/1987	H1N9	HA	HA0	YES	NO	SinoBiological			ABK40634.1	Baculovirus	His tag
40136-V08H1	A/mallard/Ohio/265/1987	H1N9	HA	HA1	YES	NO	SinoBiological			ABK40634.1	Human cells	His tag
40140-V08B	A/swine/Korea/PZ72-1/2006	H3N1	HA	HA0	NO	YES	SinoBiological			ACS71642.1	Baculovirus	His tag
40140-V08H1	A/swine/Korea/PZ72-1/2006	H3N1	HA	HA1	NO	YES	SinoBiological			ACS71642.1	Human cells	His tag
40145-V08H1	A/Victoria/361/2011	H3N2	HA	HA1	NO	YES	SinoBiological			AGB08328.1	Human cells	His tag
40146-V08B	A/Hong Kong/CUHK31987/2011	H3N2	HA	HA0	NO	YES	SinoBiological			AGC13545.1	Baculovirus	His tag
40149-V08B	A/Sydney/5/1997	H3N2	HA	HA0	NO	YES	SinoBiological			ACO95259.1	Baculovirus	His tag
40151-V08B	A/Victoria/208/2009	H3N2	HA	HA0	NO	YES	SinoBiological			ADG21005.1	Baculovirus	His tag
40152-V08B	A/Guangdong-Luohu/1256/2009	H3N2	HA	HA0	NO	YES	SinoBiological			AFM72872.1	Baculovirus	His tag
40153-V08B	A/Babol/36/2005	H3N2	HA	HA0	NO	YES	SinoBiological				Baculovirus	His tag
40154-V08B	A/Moscow/10/1999	H3N2	HA	HA0	NO	YES	SinoBiological			ABE73115.1	Baculovirus	His tag
40155-V08B	A/equine/Gansu/7/2008 (B)	H3N8	HA	HA1	NO	YES	SinoBiological			ACE81938.1	Baculovirus	His tag
40155-V08H1	A/equine/Gansu/7/2008 (H)	H3N8	HA	HA1	NO	YES	SinoBiological			ACE81938.1	Human cells	His tag
40157-V08H1	B/Yamagata/16/1988	FluB	HA	HA1	NO	NO	SinoBiological				Human cells	His tag
40158-V08B	A/chicken/VietNam/NCVD-016/2008 (Bi)	H5N1	HA	HA0	YES	NO	SinoBiological			ACO07033.1	Baculovirus	His tag
40158-V08B2	A/chicken/VietNam/NCVD-016/2008 (Bii)	H5N1	HA	HA0	YES	NO	SinoBiological	uncleaved	Mut clv site	ACO07033.1	Baculovirus	His tag
40158-V08H1	A/chicken/VietNam/NCVD-016/2008	H5N1	HA	HA1	YES	NO	SinoBiological			ACO07033.1	Human cells	His tag
40160-V08B	A/barnswallow/HongKong/D10-1161/2010 (Bi)	H5N1	HA	HA0	YES	NO	SinoBiological			AGC13463.1	Baculovirus	His tag
40160-V08B1	A/barnswallow/HongKong/D10-1161/2010 (Bii)	H5N1	HA	HA0	YES	NO	SinoBiological	uncleaved	Mut clv site		Baculovirus	His tag
40160-V08H1	A/barn swallow/Hong Kong/D10-1161/2010	H5N1	HA	HA1	YES	NO	SinoBiological			AGC13463.1	Human cells	His tag
40164-V08B2	A/turkey/Ireland/1378/1983	H5N8	HA	HA2	YES	NO	SinoBiological			ABI85117.1	Baculovirus	His tag
40164-V08H1	A/turkey/Ireland/1378/1983	H5N8	HA	HA1	YES	NO	SinoBiological			P11135	Human cells	His tag
40165-V08B	A/chicken/Italy/22A/1998	H5N9	HA	HA0	YES	NO	SinoBiological			ABR37720.1	Baculovirus	His tag
40165-V08H1	A/chicken/Italy/22A/1998	H5N9	HA	HA1	YES	NO	SinoBiological			ABR37720.1	Human cells	His tag
40166-V08H1	A/duck/Shantou/83/2000	H6N2	HA	HA1	YES	NO	SinoBiological			ADG44842.1	Human cells	His tag
40167-V08H1	A/shearwater/Australia/1/1973	H6N5	HA	HA1	YES	NO	SinoBiological			ADD64203.1	Human cells	His tag
40168-V08B	A/mallard/Ohio/217/1998	H6N8	HA	HA0	YES	NO	SinoBiological			ABO52049.1	Baculovirus	His tag
40168-V08H1	A/mallard/Ohio/217/1998	H6N8	HA	HA1	YES	NO	SinoBiological			ABO52049.1	Human cells	His tag
40169-V08H1	A/turkey/Italy/4602/99	H7N1	HA	HA1	NO	YES	SinoBiological			CAD38286.1	Human cells	His tag

Short ID	Long ID	Subtype	Mol ID1	Mol ID2	Is Group 1	Is Group 2	Source	Form1	Form2	GenBank	Expression System	Notes
40170-V08B	A/ruddy turnstone/New Jersey/563/2006	H7N2	HA	HA0	NO	YES	SinoBiological			ACS68445.1	Baculovirus	His tag
40170-V08H1	A/ruddy turnstone/New Jersey/563/2006	H7N2	HA	HA1	NO	YES	SinoBiological			ACS68445.1	Human cells	His tag
40171-V08B	A/equine/Kentucky/1a/1975	H7N7	HA	HA0	NO	YES	SinoBiological			ACL12085.1	Baculovirus	His tag
40172-V08B	A/mallard/Netherlands/33/2006	H7N8	HA	HA0	NO	YES	SinoBiological			ACR59554.1	Baculovirus	His tag
40172-V08H1	A/mallard/Netherlands/33/2006	H7N8	HA	HA1	NO	YES	SinoBiological			EPI182113	Human cells	His tag
40174-V08B	A/Hong Kong/35820/2009	H9N2	HA	HA0	YES	NO	SinoBiological			ADC41853.1	Baculovirus	His tag
40174-V08H1	A/Hong Kong/35820/2009	H9N2	HA	HA1	YES	NO	SinoBiological				Human cells	His tag
40181-V08B	A/shorebird/DE/261/2003	H9N5	HA	HA0	YES	NO	SinoBiological			ABB87950.1	Baculovirus	His tag
40181-V08H1	A/shorebird/DE/261/2003	H9N5	HA	HA1	YES	NO	SinoBiological			ABB87950.1	Human cells	His tag
40184-V08B	A/mallard/Minnesota/Sg-00194/2007	H10N3	HA	HA0	NO	YES	SinoBiological			ACT84107.1	Baculovirus	His tag
40184-V08H1	A/mallard/Minnesota/Sg-00194/2007	H10N3	HA	HA1	NO	YES	SinoBiological			ACT84107.1	Human cells	His tag
40188-V08H1	A/duck/England/1/1956	H11N6	HA	HA1	YES	NO	SinoBiological			AGB50949.1	Human cells	His tag
40189-V08H1	A/bar headed goose/Mongolia/143/2005	H12N3	HA	HA1	YES	NO	SinoBiological			ACV86810.1	Human cells	His tag
40192-V08H1	A/mallard/Astrakhan/263/1982	H14N5	HA	HA1	NO	YES	SinoBiological			P26136	Human cells	His tag
40193-V08B	A/Australian shelduck/Western Australia/1756/1983	H15N2	HA	HA0	NO	YES	SinoBiological			ABB90704.1	Baculovirus	His tag
40193-V08H1	A/Australian shelduck/Western Australia/1756/1983	H15N2	HA	HA1	NO	YES	SinoBiological			ABB90704.1	Human cells	His tag
40239-V08B	A/Shanghai/2/2013 (B)	H7N9	HA	HA0	NO	YES	SinoBiological				Baculovirus	His tag
40239-V08H	A/Shanghai/2/2013 (H)	H7N9	HA	HA0	NO	YES	SinoBiological				Human cells	His tag
40324-V08B	A/flat-faced bat/Peru/033/2010	H18N11	HA	HA0	YES	NO	SinoBiological			AGX84934.1	Baculovirus	His tag
40324-V08H1	A/flat-faced bat/Peru/033/2010	H18N11	HA	HA1	YES	NO	SinoBiological			AGX84934.1	Human cells	His tag
40325-V08B	A/Zhejiang/DTID-ZJU10/2013 (B)	H7N9	HA	HA0	NO	YES	SinoBiological			AHA11500.1	Baculovirus	His tag
40325-V08H	A/Zhejiang/DTID-ZJU10/2013 (H)	H7N9	HA	HA0	NO	YES	SinoBiological			AHA11500.1	Human cells	His tag
40350-V08H1	A/California/06/2009	H1N1	HA	HA1	YES	NO	SinoBiological			ACP41935.1	Human cells	His tag
40351-V08H1	A/duck/Guangdong/E1/2012	H10N8	HA	HA1	NO	YES	SinoBiological				Human cells	His tag
40354-V08H1	A/Texas/50/2012	H3N2	HA	HA1	NO	YES	SinoBiological			AFH57070.1	Human cells	His tag
40359-V08B	A/Jiangxi-Donghu/346/2013 (Bi)	H10N8	HA	HA0	NO	YES	SinoBiological			EPI497477	Baculovirus	His tag
40359-VNAB	A/Jiangxi-Donghu/346/2013 (HA0; Bii)	H10N8	HA	HA0	NO	YES	SinoBiological			EPI497477	Baculovirus	
40360-V08H1	A/duck/Hunan/S11205/2012	H10N3	HA	HA1	NO	YES	SinoBiological			AGO87051.1	Human cells	His tag
40372-V08B	A/chicken/Jilin/9/2004	H5N1	HA	HA0	YES	NO	SinoBiological			AAT76166.1	Baculovirus	His tag
40372-V08H1	A/chicken/Jilin/9/2004	H5N1	HA	HA1	YES	NO	SinoBiological				Human cells	His tag

Supplementary Table 3 List of Proteins on Array #2

Short ID	Long ID	Subtype	Mol ID1	Mol ID2	Is Group 1	Is Group 2	Source	Form1	Form2	GenBank	Expression System	Notes
10003-V04H2	A/VietNam/1203/2004	H5N1	HA	HA2	YES	NO	SinoBiological			AAW80717.1	Human cells	Fc tag (mouse)
10003-V06H1	A/VietNam/1203/2004	H5N1	HA	HA1	YES	NO	SinoBiological			AAW80717.1	Human cells	His
Fc tag (mouse)												
10003-V06H3	A/VietNam/1203/2004	H5N1	HA	HA0	YES	NO	SinoBiological	uncleaved		AAW80717.1	Human cells	His
Fc tag (mouse)												
11048-V06H1	A/Anhui/1/2005	H5N1	HA	HA0	YES	NO	SinoBiological	uncleaved		ABD28180.1	Human cells	His
Fc tag (mouse)												
11048-V08B	A/Anhui/1/2005 (B)	H5N1	HA	HA0	YES	NO	SinoBiological	uncleaved		ABD28180.1	Baculovirus	His tag
11048-V08H1.1	A/Anhui/1/2005 (H)	H5N1	HA	HA0	YES	NO	SinoBiological	uncleaved		ABD28180.1	Human cells	His tag
11048-V08H2	A/Anhui/1/2005	H5N1	HA	HA1	YES	NO	SinoBiological			ABD28180.1	Human cells	His tag
11048-V08H4	A/Anhui/1/2005	H5N1	HA	HA0	YES	NO	SinoBiological	cleaved	Native	ABD28180.1	Human cells	His tag
11048-VNAH2	A/Anhui/1/2005	H5N1	HA	HA1	YES	NO	SinoBiological			ABD 28180.1	Human cells	
11052-V08H	A/Brisbane/59/2007	H1N1	HA	HA0	YES	NO	SinoBiological	uncleaved	Native	ACA28844.1	Human cells	His tag
11052-V08H1	A/Brisbane/59/2007	H1N1	HA	HA1	YES	NO	SinoBiological			ACA28844.1	Human cells	His tag
11055-V08B	A/California/04/2009 (B)	H1N1	HA	HA0	YES	NO	SinoBiological	uncleaved	Native	ACP41105.1	Baculovirus	His tag
11055-V08H	A/California/04/2009 (H)	H1N1	HA	HA0	YES	NO	SinoBiological	uncleaved	Native	ACP41105.1	Human cells	His tag
11055-V08H2	A/California/04/2009	H1N1	HA	HA0	YES	NO	SinoBiological	cleaved (partial)		ACP41105.1	Human cells	His tag
11055-V08H4	A/California/04/2009	H1N1	HA	HA1	YES	NO	SinoBiological			ACP41105.1	Human cells	His tag
11055-VNAB	A/California/04/2009	H1N1	HA	HA0	YES	NO	SinoBiological				Baculovirus	
11056-V08B	A/Brisbane/10/2007 (B)	H3N2	HA	HA0	NO	YES	SinoBiological			ABW23353.1	Baculovirus	His tag
11056-V08H	A/Brisbane/10/2007 (H)	H3N2	HA	HA0	NO	YES	SinoBiological	uncleaved	Native	ABW23353.1	Human cells	His tag
11056-V08H1	A/Brisbane/10/2007	H3N2	HA	HA1	NO	YES	SinoBiological			ABW23353.1	Human cells	His tag
11059-V08B1	A/bar-headed goose/Qinghai/14/2008 (B)	H5N1	HA	HA0	YES	NO	SinoBiological	uncleaved		ACL28277.1	Baculovirus	His tag
11059-V08H1	A/bar-headed goose/Qinghai/14/2008 (H)	H5N1	HA	HA0	YES	NO	SinoBiological	uncleaved		ACL28277.1	Human cells	His tag
11059-V08H2	A/bar-headed goose/Qinghai/14/2008	H5N1	HA	HA0	YES	NO	SinoBiological	cleaved	Native	ACL28277.1	Human cells	His tag
11060-V08H1	A/Indonesia/5/2005	H5N1	HA	HA0	YES	NO	SinoBiological	uncleaved		ABW06108.1	Human cells	His tag
11060-V08H2	A/Indonesia/5/2005	H5N1	HA	HA0	YES	NO	SinoBiological	cleaved	Native	ABW06108.1	Human cells	His tag
11061-V08H1	A/turkey/Turkey/1/2005	H5N1	HA	HA0	YES	NO	SinoBiological	uncleaved		ABD73284.1	Human cells	His tag
11061-V08H2	A/turkey/Turkey/1/2005	H5N1	HA	HA0	YES	NO	SinoBiological	cleaved	Native	ABD73284.1	Human cells	His tag
11062-V08H1	A/Vietnam/1194/2004	H5N1	HA	HA0	YES	NO	SinoBiological	uncleaved		AAT73273.1	Human cells	His tag
11062-V08H2	A/Vietnam/1194/2004	H5N1	HA	HA0	YES	NO	SinoBiological	cleaved	Native	AAT73273.1	Human cells	His tag
11068-V08H	A/Brevig Mission/1/1918	H1N1	HA	HA0	YES	NO	SinoBiological	uncleaved	Native	AAD17229.1	Human cells	His tag
11068-V08H1	A/Brevig Mission/1/1918	H1N1	HA	HA1	YES	NO	SinoBiological			AAD17229.1	Human cells	His tag
11082-V08B	A/Netherlands/219/03	H7N7	HA	HA0	NO	YES	SinoBiological		Native	AAR02640.1	Baculovirus	His tag
11082-V08H1	A/Netherlands/219/03	H7N7	HA	HA1	NO	YES	SinoBiological			AAR02640.1	Human cells	His tag
11212-V08B	A/chicken/Netherlands/1/03	H7N7	HA	HA0	NO	YES	SinoBiological		Native	AAR02639.1	Baculovirus	His tag
11212-V08H1	A/chicken/Netherlands/1/03	H7N7	HA	HA1	NO	YES	SinoBiological			AAR02639.1	Human cells	His tag
11229-V08H	A/Hong Kong/1073/99	H9N2	HA	HA0	YES	NO	SinoBiological		Native	$NP_8$59037.1	Human cells	His tag
11229-V08H1	A/HongKong/1073/99	H9N2	HA	HA1	YES	NO	SinoBiological			$NP_8$59037.1	Human cells	His tag
11683-V08H	A/New Caledonia/20/99	H1N1	HA	HA0	YES	NO	SinoBiological	uncleaved		AAP34324.1	Human cells	His tag
11683-V08H1	A/New Caledonia/20/99	H1N1	HA	HA1	YES	NO	SinoBiological			AAP34324.1	Human cells	His tag
11685-V08H	A/duck/NZL/160/1976	H1N3	HA	HA0	YES	NO	SinoBiological	uncleaved		ABB20429.1	Human cells	His tag
11685-V08H1	A/duck/NZL/160/1976	H1N3	HA	HA1	YES	NO	SinoBiological			ABB20429.1	Human cells	His tag
11686-V08H1	A/chicken/Egypt/2253-1/2006	H5N1	HA	HA1	YES	NO	SinoBiological			ABG81039.1	Human cells	His tag
11687-V08H	A/Ohio/UR06-0091/2007	H1N1	HA	HA0	YES	NO	SinoBiological	uncleaved		ABW40422.1	Human cells	His tag
11687-V08H1	A/Ohio/UR06-0091/2007	H1N1	HA	HA1	YES	NO	SinoBiological			ABW40422.1	Human cells	His tag
11689-V08H	A/Hong Kong/483/97	H5N1	HA	HA0	YES	NO	SinoBiological	uncleaved	Mut clv site	AAC32099.1	Human cells	His tag
11689-V08H1	A/Hong Kong/483/97	H5N1	HA	HA1	YES	NO	SinoBiological			AAC32099.1	Human cells	His tag
11690-V08H	A/goose/Guiyang/337/2006	H5N1	HA	HA0	YES	NO	SinoBiological	uncleaved	Mut clv site	ABJ96698.1	Human cells	His tag
11690-V08H1	A/goose/Guiyang/337/2006	H5N1	HA	HA1	YES	NO	SinoBiological			ABJ96698.1	Human cells	His tag
11693-V08H	A/duck/Hong Kong/786/1979 (H)	H10N3	HA	HA0	NO	YES	SinoBiological	uncleaved		BAF46762.1	Human cells	His tag
11694-V08H	A/Japanese white-eye/Hong Kong/1038/2006	H5N1	HA	HA0	YES	NO	SinoBiological	uncleaved	Mut clv site	ABJ96775.1	Human cells	His tag
11694-V08H1	A/Japanese white-eye/Hong Kong/1038/2006	H5N1	HA	HA1	YES	NO	SinoBiological			ABJ96775.1	Human cells	His tag

Short ID	Long ID	Subtype	Mol ID1	Mol ID2	Is Group 1	Is Group 2	Source	Form1	Form2	GenBank	Expression System	Notes
11696-V08H	A/duck/Hokkaido/167/2007	H5N3	HA	HA0	YES	NO	SinoBiological			BAG07130.2	Human cells	His tag
11696-V08H1	A/duck/Hokkaido/167/2007	H5N3	HA	HA1	YES	NO	SinoBiological			BAG07130.2	Human cells	His tag
11697-V08H	A/Egypt/2321-NAMRU3/2007	H5N1	HA	HA0	YES	NO	SinoBiological	uncleaved	Mut clv site	ABP96850.1	Human cells	His tag
11697-V08H1	A/Egypt/2321-NAMRU3/2007	H5N1	HA	HA1	YES	NO	SinoBiological			ABP96850.1	Human cells	His tag
11698-V08H	A/duck/Hunan/795/2002	H5N1	HA	HA0	YES	NO	SinoBiological	uncleaved	Mut clv site	ACA47835.1	Human cells	His tag
11698-V08H1	A/duck/Hunan/795/2002	H5N1	HA	HA1	YES	NO	SinoBiological			ACA47835.1	Human cells	His tag
11699-V08H	A/American green-winged teal/California/HKWF609/2007	H5N2	HA	HA0	YES	NO	SinoBiological	uncleaved		ACF47563.1	Human cells	His tag
11699-V08H1	A/American green-winged teal/California/HKWF609/2007	H5N2	HA	HA1	YES	NO	SinoBiological			ACF47563.1	Human cells	His tag
11700-V08H	A/Common magpie/Hong Kong/2256/2006	H5N1	HA	HA0	YES	NO	SinoBiological	uncleaved	Mut clv site	ABJ96777.1	Human cells	His tag
11700-V08H1	A/Common magpie/Hong Kong/2256/2006	H5N1	HA	HA1	YES	NO	SinoBiological			ABJ96777.1	Human cells	His tag
11701-V08H1	A/duck/Laos/3295/2006	H5N1	HA	HA1	YES	NO	SinoBiological			ABG67978.1	Human cells	His tag
11702-V08H	A/Egypt/N05056/2009	H5N1	HA	HA0	YES	NO	SinoBiological	uncleaved	Mut clv site	ACT15357.1	Human cells	His tag
11702-V08H1	A/Egypt/N05056/2009	H5N1	HA	HA1	YES	NO	SinoBiological			ACT15357.1	Human cells	His tag
11708-V08H	A/Solomon Islands/3/2006	H1N1	HA	HA0	YES	NO	SinoBiological	uncleaved		ABU99109.1	Human cells	His tag
11708-V08H1	A/Solomon Islands/3/2006	H1N1	HA	HA1	YES	NO	SinoBiological			ABU99109.1	Human cells	His tag
11709-V08H	A/whooper swan/Mongolia/244/2005	H5N1	HA	HA0	YES	NO	SinoBiological	uncleaved	Mut clv site	ACZ36881.1	Human cells	His tag
11709-V08H1	A/whooper swan/Mongolia/244/2005	H5N1	HA	HA1	YES	NO	SinoBiological			ACZ36881.1	Human cells	His tag
11710-V08B	A/Cambodia/R0405050/2007 (B)	H5N1	HA	HA0	YES	NO	SinoBiological				Baculovirus	His tag
11710-V08H	A/Cambodia/R0405050/2007 (H)	H5N1	HA	HA0	YES	NO	SinoBiological	uncleaved	Mut clv site	ACI06178.1	Human cells	His tag
11710-V08H1	A/Cambodia/R0405050/2007	H5N1	HA	HA1	YES	NO	SinoBiological			ACI06178.1	Human cells	His tag
11712-V08B	A/chicken/India/NIV33487/06 (B)	H5N1	HA	HA0	YES	NO	SinoBiological			ABQ45850.1	Baculovirus	His tag
11712-V08H	A/chicken/India/NIV33487/06 (H)	H5N1	HA	HA0	YES	NO	SinoBiological	uncleaved	Mut clv site	ABQ45850.1	Human cells	His tag
11712-V08H1	A/chicken/India/NIV33487/06	H5N1	HA	HA1	YES	NO	SinoBiological			ABQ45850.1	Human cells	His tag
11713-V08H	A/Hong kong/213/2003	H5N1	HA	HA0	YES	NO	SinoBiological	uncleaved	Mut clv site	ABP51975.1	Human cells	His tag
11713-V08H1	A/Hong kong/213/2003	H5N1	HA	HA1	YES	NO	SinoBiological		1 aa mut	ABP51975.1	Human cells	His tag
11715-V08H	A/Wyoming/03/2003	H3N2	HA	HA0	NO	YES	SinoBiological	uncleaved		ABX10525.1	Human cells	His tag
11715-V08H1	A/Wyoming/03/2003	H3N2	HA	HA1	NO	YES	SinoBiological			ABX10525.1	Human cells	His tag
11716-V08H	B/Malaysia/2506/2004	B	HA	HA0	NO	NO	SinoBiological	uncleaved		ACO05957.1	Human cells	His tag
11716-V08H1	B/Malaysia/2506/2004	B	HA	HA1	NO	NO	SinoBiological			ACO05957.1	Human cells	His tag
11717-V08H	A/duck/NY/191255-59/2002	H5N8	HA	HA0	YES	NO	SinoBiological	uncleaved		AAP72011.1	Human cells	His tag
11717-V08H1	A/duck/NY/191255-59/2002	H5N8	HA	HA1	YES	NO	SinoBiological			AAP72011.1	Human cells	His tag
11972-V08B	A/Wisconsin/67/X-161/2005 (B)	H3N2	HA	HA0	NO	YES	SinoBiological			ACF41911.1	Baculovirus	His tag
11972-V08H	A/Wisconsin/67/X-161/2005 (H)	H3N2	HA	HA0	NO	YES	SinoBiological	uncleaved		ABO37609.1	Human cells	His tag
11972-V08H1	A/Wisconsin/67/X-161/2005	H3N2	HA	HA1	NO	YES	SinoBiological			ABO37609.1	Human cells	His tag
40001-V08H	A/Duck/Hong Kong/p46/97	H5N1	HA	HA0	YES	NO	SinoBiological	uncleaved	Mut clv site	AAF02306.1	Human cells	His tag
40001-V08H1	A/Duck/Hong Kong/p46/97	H5N1	HA	HA1	YES	NO	SinoBiological			AAF02306.1	Human cells	His tag
40004-V08H	A/Xinjiang/1/2006	H5N1	HA	HA0	YES	NO	SinoBiological	uncleaved	Mut clv site	ACJ68614.1	Human cells	His tag
40004-V08H1	A/Xinjiang/1/2006	H5N1	HA	HA1	YES	NO	SinoBiological			ACJ68614.1	Human cells	His tag
40014-V08H1	A/ostrich/South Africa/AI1091/2006	H5N2	HA	HA1	YES	NO	SinoBiological			ABQ24010.1	Human cells	His tag
40015-V08B.1	A/Hubei/1/2010 (B)	H5N1	HA	HA0	YES	NO	SinoBiological			AEO89181.1	Baculovirus	His tag
40015-V08H	A/Hubei/1/2010 (H)	H5N1	HA	HA0	YES	NO	SinoBiological	cleaved			Human cells	His tag
40022-V08H1	A/Vietnam/UT31413II/2008	H5N1	HA	HA1	YES	NO	SinoBiological			ADF83651.1	Human cells	His tag
40024-V08B	A/Goose/Guangdong/1/96	H5N1	HA	HA0	YES	NO	SinoBiological			YP$_{3}$08669.1	Baculovirus	His tag
40043-V08H	A/Perth/16/2009	H3N2	HA	HA0	NO	YES	SinoBiological			ACS71642.1	Human cells	His tag
40043-V08H1	A/Perth/16/2009	H3N2	HA	HA1	NO	YES	SinoBiological			ACS71642.1	Human cells	His tag
40044-V08H	A/common magpie/Hong Kong/5052/2007	H5N1	HA	HA0	YES	NO	SinoBiological			ACJ26242.1	Human cells	His tag
40044-V08H1	A/common magpie/Hong Kong/5052/2007	H5N1	HA	HA1	YES	NO	SinoBiological			ACJ26242.1	Human cells	His tag
40049-V08H1	A/Egypt/3300-NAMRU3/2008	H5N1	HA	HA1	YES	NO	SinoBiological				Human cells	His tag
40060-V08H1	A/Hubei/1/2010	H5N1	HA	HA1	YES	NO	SinoBiological			AEO89181.1	Human cells	His tag
40064-V07H	A/Thailand/1(KAN-1)/2004	H5N1			NO	NO	SinoBiological				Human cells	His tag

Short ID	Long ID	Subtype	Mol ID1	Mol ID2	Is Group 1	Is Group 2	Source	Form1	Form2	GenBank	Expression System	Notes
40064-V07H-B	A/Thailand/1(KAN-1)/2004	H5N1			NO	NO	SinoBiological				Baculovirus	His tag + Bi-otin
40065-V08H1	A/Thailand/1(KAN-1)/2004	H5N1	HA	HA1	YES	NO	SinoBiological				Human cells	His tag
40103-V08H	A/Anhui/1/2013 (H)	H7N9	HA	HA0	NO	YES	SinoBiological			EPI439507	Human cells	His tag
40103-V08H1	A/Anhui/1/2013	H7N9	HA	HA1	NO	YES	SinoBiological			AGJ51953.1	Human cells	His tag
40103-V08H4	A/Anhui/1/2013	H7N9	HA	HA0	NO	YES	SinoBiological	cleaved		EPI439507	Human cells	His tag
40104-V08B	A/Shanghai/1/2013 (B)	H7N9	HA	HA0	NO	YES	SinoBiological				Baculovirus	His tag
40104-V08B1	A/Shanghai/1/2013 (B)	H7N9	HA	HA1	NO	YES	SinoBiological				Baculovirus	His tag
40104-V08H	A/Shanghai/1/2013 (Hi)	H7N9	HA	HA0	NO	YES	SinoBiological				Human cells	His tag
40104-V08H1	A/Shanghai/1/2013 (H)	H7N9	HA	HA1	NO	YES	SinoBiological				Human cells	His tag
40104-V08H4	A/Shanghai/1/2013 (Hii)	H7N9	HA	HA0	NO	YES	SinoBiological	cleaved			Human cells	His tag
40105-V08B	A/Hangzhou/1/2013 (B)	H7N9	HA	HA0	NO	YES	SinoBiological			AGI60301.1	Baculovirus	His tag
40105-V08H	A/Hangzhou/1/2013 (H)	H7N9	HA	HA0	NO	YES	SinoBiological			AGI60301.1	Human cells	His tag
40105-V08H1	A/Hangzhou/1/2013	H7N9	HA	HA1	NO	YES	SinoBiological			AGI60301.1	Human cells	His tag
40106-V08H	A/Pigeon/Shanghai/S1069/2013 (H)	H7N9	HA	HA0	NO	YES	SinoBiological				Human cells	His tag
40109-V07H	A/Shanghai/1/2013	H7N9			NO	NO	SinoBiological				Human cells	His tag
40109-VNAHC	A/Shanghai/1/2013	H7N9			NO	NO	SinoBiological				Human cells	bio-activity tested
40111-V08B	A/Shanghai/2/2013	H7N9	NP	NP	NO	NO	SinoBiological			AGL44439.1	Baculovirus	His tag
40116-V08B	A/Hong Kong/1/1968	H3N2	HA	HA0	NO	YES	SinoBiological			Q91MA7	Baculovirus	His tag
40116-V08H1	A/Hong Kong/1/1968	H3N2	HA	HA1	NO	YES	SinoBiological			AAK51718.1	Human cells	His tag
40117-V08B	A/bar-headed goose/Qinghai/1A/2005	H5N1	HA	HA0	YES	NO	SinoBiological			ABF93441.1	Baculovirus	His tag
40117-V08H1	A/bar-headed goose/Qinghai/1A/2005	H5N1	HA	HA1	YES	NO	SinoBiological			ABF93441.1	Human cells	His tag
40119-V08B	A/Guiyang/1/1957	H2N2	HA	HA0	YES	NO	SinoBiological			ACD85231.1	Baculovirus	His tag
40119-V08H1	A/Guiyang/1/1957	H2N2	HA	HA1	YES	NO	SinoBiological			ACD85231.1	Human cells	His tag
40120-V08B	A/Fujian/411/2002	H3N2	HA	HA0	NO	YES	SinoBiological			AFG72823.1	Baculovirus	His tag
40123-V08B	A/Hangzhou/3/2013	H7N9	HA	HA0	NO	YES	SinoBiological			EPI442713	Baculovirus	His tag
40125-V08B	A/Zhejiang/1/2013	H7N9	HA	HA0	NO	YES	SinoBiological			EPI443034	Baculovirus	His tag
40126-V08B	A/Shanghai/4664T/2013	H7N9	HA	HA0	NO	YES	SinoBiological			AGI60292.1	Baculovirus	His tag
40128-V08B	A/turkey/Italy/214845/2002	H7N3	HA	HA0	NO	YES	SinoBiological			CAF33017.1	Baculovirus	His tag
40128-V08H1	A/turkey/Italy/214845/2002	H7N3	HA	HA1	NO	YES	SinoBiological			CAF33017.1	Human cells	His tag
40129-V08H1	A/chicken/SK/HR-00011/2007	H7N3	HA	HA1	NO	YES	SinoBiological			ACA25329.1	Human cells	His tag
40134-V08B	A/USSR/90/1977	H1N1	HA	HA0	YES	NO	SinoBiological			P03453.2	Baculovirus	His tag
40134-V08H1	A/USSR/90/1977	H1N1	HA	HA1	YES	NO	SinoBiological			P03453	Human cells	His tag
40146-V08B	A/Hong Kong/CUHK31987/2011	H3N2	HA	HA0	NO	YES	SinoBiological			AGC13545.1	Baculovirus	His tag
40149-V08B	A/Sydney/5/1997	H3N2	HA	HA0	NO	YES	SinoBiological			ACO95259.1	Baculovirus	His tag
40153-V08B	A/Babol/36/2005	H3N2	HA	HA0	NO	YES	SinoBiological				Baculovirus	His tag
40154-V08B	A/Moscow/10/1999	H3N2	HA	HA0	NO	YES	SinoBiological			ABE73115.1	Baculovirus	His tag
40158-V08B	A/chicken/VietNam/NCVD-016/2008 (Bi)	H5N1	HA	HA0	YES	NO	SinoBiological			ACO07033.1	Baculovirus	His tag
40158-V08B2	A/chicken/VietNam/NCVD-016/2008 (Bii)	H5N1	HA	HA0	YES	NO	SinoBiological	uncleaved	Mut clv site	ACO07033.1	Baculovirus	His tag
40158-V08H1	A/chicken/VietNam/NCVD-016/2008	H5N1	HA	HA1	YES	NO	SinoBiological			ACO07033.1	Human cells	His tag
40160-V08B	A/barnswallow/HongKong/D10-1161/2010 (Bi)	H5N1	HA	HA0	YES	NO	SinoBiological			AGC13463.1	Baculovirus	His tag
40160-V08B1	A/barnswallow/HongKong/D10-1161/2010 (Bii)	H5N1	HA	HA0	YES	NO	SinoBiological	uncleaved	Mut clv site		Baculovirus	His tag
40160-V08H1	A/barn swallow/Hong Kong/D10-1161/2010	H5N1	HA	HA1	YES	NO	SinoBiological			AGC13463.1	Human cells	His tag
40164-V08B2	A/turkey/Ireland/1378/1983	H5N8	HA	HA2	YES	NO	SinoBiological			ABI85117.1	Baculovirus	His tag
40164-V08H1	A/turkey/Ireland/1378/1983	H5N8	HA	HA1	YES	NO	SinoBiological			P11135	Human cells	His tag
40165-V08B	A/chicken/Italy/22A/1998	H5N9	HA	HA0	YES	NO	SinoBiological			ABR37720.1	Baculovirus	His tag
40165-V08H1	A/chicken/Italy/22A/1998	H5N9	HA	HA1	YES	NO	SinoBiological			ABR37720.1	Human cells	His tag
40168-V08B	A/mallard/Ohio/217/1998	H6N8	HA	HA0	YES	NO	SinoBiological			ABO52049.1	Baculovirus	His tag
40168-V08H1	A/mallard/Ohio/217/1998	H6N8	HA	HA1	YES	NO	SinoBiological			ABO52049.1	Human cells	His tag
40169-V08H1	A/turkey/Italy/4602/99	H7N1	HA	HA1	NO	YES	SinoBiological			CAD38286.1	Human cells	His tag

Short ID	Long ID	Subtype	Mol ID1	Mol ID2	Is Group 1	Is Group 2	Source	Form1	Form2	GenBank	Expression System	Notes
40170-V08B	A/ruddy turnstone/New Jersey/563/2006	H7N2	HA	HA0	NO	YES	SinoBiological			ACS68445.1	Baculovirus	His tag
40170-V08H1	A/ruddy turnstone/New Jersey/563/2006	H7N2	HA	HA1	NO	YES	SinoBiological			ACS68445.1	Human cells	His tag
40171-V08B	A/equine/Kentucky/1a/1975	H7N7	HA	HA0	NO	YES	SinoBiological			ACL12085.1	Baculovirus	His tag
40172-V08B	A/mallard/Netherlands/33/2006	H7N8	HA	HA0	NO	YES	SinoBiological			ACR59554.1	Baculovirus	His tag
40172-V08H1	A/mallard/Netherlands/33/2006	H7N8	HA	HA1	NO	YES	SinoBiological			EPI182113	Human cells	His tag
40239-V08B	A/Shanghai/2/2013 (B)	H7N9	HA	HA0	NO	YES	SinoBiological				Baculovirus	His tag
40239-V08H	A/Shanghai/2/2013 (H)	H7N9	HA	HA0	NO	YES	SinoBiological				Human cells	His tag
40325-V08B	A/Zhejiang/DTID-ZJU10/2013 (B)	H7N9	HA	HA0	NO	YES	SinoBiological			AHA11500.1	Baculovirus	His tag
40325-V08H	A/Zhejiang/DTID-ZJU10/2013 (H)	H7N9	HA	HA0	NO	YES	SinoBiological			AHA11500.1	Human cells	His tag
40354-V08B	A/Texas/50/2012	H3N2	HA	HA0	NO	YES	SinoBiological			EPI537015	Baculovirus	His tag
40354-V08H1	A/Texas/50/2012	H3N2	HA	HA1	NO	YES	SinoBiological			AFH57070.1	Human cells	His tag
40372-V08B	A/chicken/Jilin/9/2004	H5N1	HA	HA0	YES	NO	SinoBiological			AAT76166.1	Baculovirus	His tag
40372-V08H1	A/chicken/Jilin/9/2004	H5N1	HA	HA1	YES	NO	SinoBiological				Human cells	His tag
1918	A/South Carolina/1/1918 (H1)	H1N1	HA	HA0	YES	NO	F. Krammer Laboratory				Baculovirus	His tag
AHT	A/Anhui/1/2013 (H7)	H7N9	HA	HA0	NO	YES	F. Krammer Laboratory				Baculovirus	His tag
Alabama H3	A/Alabama/1/1981 (H3)	H3N2	HA	HA0	NO	YES	F. Krammer Laboratory				Baculovirus	His tag
Anhui07	A/Anhui/1/2013 (H7)	H7N9	HA	HA0	NO	YES	F. Krammer Laboratory				Baculovirus	His tag
asH1	A/swine/Jiangsu/40/2011	H1N1	HA	HA0	YES	NO	F. Krammer Laboratory				Baculovirus	His tag
B/ Wisc NA	B/Wisconsin/1/2010 (B-NA)	B			NO	NO	F. Krammer Laboratory				Baculovirus	His tag
B/Bris NA	B/Brisbane/60/2008 (B-NA)	B			NO	NO	F. Krammer Laboratory				Baculovirus	His tag
B/Flo HA	B/Florida/4/2006	B	HA	HA0	NO	NO	F. Krammer Laboratory				Baculovirus	His tag
B/Flo NA	B/Florida/4/2006 (B-NA)	B			NO	NO	F. Krammer Laboratory				Baculovirus	His tag
B/Flo NA.1	B/Florida/4/2006 (B-NA)	B			NO	NO	F. Krammer Laboratory				Baculovirus	His tag
B/Lee HA	B/Lee/1940	B	HA	HA0	NO	NO	F. Krammer Laboratory				Baculovirus	His tag
B/Mal HA	B/Malaysia/2506/2004	B	HA	HA0	NO	NO	F. Krammer Laboratory				Baculovirus	His tag
B/Mal NA$_0$1/25/2018	B/Malaysia/2506/2004 (B-NA)	B			NO	NO	F. Krammer Laboratory				Baculovirus	His tag
B/Mal NA$_0$9/25/2017	B/Malaysia/2506/2004 (B-NA)	B			NO	NO	F. Krammer Laboratory				Baculovirus	His tag
B/Mal NA$_2$/2/2018	B/Malaysia/2506/2004 (B-NA)	B			NO	NO	F. Krammer Laboratory				Baculovirus	His tag
B/Mass HA	B/Massachusetts/2/2012	B	HA	HA0	NO	NO	F. Krammer Laboratory				Baculovirus	His tag
B/Phu HA	B/Phuket/3073/2013	B	HA	HA0	NO	NO	F. Krammer Laboratory				Baculovirus	His tag
B/Phuket/HA	B/Phuket/3073/2013	B	HA	HA0	NO	NO	F. Krammer Laboratory				Baculovirus	His tag
B/Vic HA	B/Victoria/2/1987	B	HA	HA0	NO	NO	F. Krammer Laboratory				Baculovirus	His tag
B/Wisc HA	B/Wisconsin/1/2010	B	HA	HA0	NO	NO	F. Krammer Laboratory				Baculovirus	His tag
B/Yam	B/Yamagata/16/1988 (B-NA)	B			NO	NO	F. Krammer Laboratory				Baculovirus	His tag

Short ID	Long ID	Subtype	Mol ID1	Mol ID2	Is Group 1	Is Group 2	Source	Form1	Form2	GenBank	Expression System	Notes
B/Yam HA	B/Yamagata/16/1988	B	HA	HA0	NO	NO	F. Krammer Laboratory				Baculovirus	His tag
B/Yam NA	B/Yamagata/16/1988 (B-NA)	B			NO	NO	F. Krammer Laboratory				Baculovirus	His tag
BC04	A/chicken/BC/CN-6/2004 (H7)	H7N3	HA	HA0	NO	YES	F. Krammer Laboratory				Baculovirus	His tag
Cal 09 H1	A/California/04/2009 (H1)	H1N1	HA	HA0	YES	NO	F. Krammer Laboratory				Baculovirus	His tag
Cal09 HA	A/California/04/2009 (H1)	H1N1	HA	HA0	YES	NO	F. Krammer Laboratory				Baculovirus	His tag
Cal09 NA	A/California/04/2009 (N1)	H1N1			NO	NO	F. Krammer Laboratory				Baculovirus	His tag
cH6/1 PR8 (6/IPR8)	cH6/1	H6	HA	HA0	YES	NO	F. Krammer Laboratory				Baculovirus	His tag
cH9/1 PR8	cH9/1	H9	HA	HA0	YES	NO	F. Krammer Laboratory				Baculovirus	His tag
Chick it h7	A/chicken/Italy/13474/1999 (H7)	H7	HA	HA0	NO	YES	F. Krammer Laboratory				Baculovirus	His tag
ckH9 (c6H9)	A/chicken/Hong Kong/G9/1997 (H9)	H9N2	HA	HA0	YES	NO	F. Krammer Laboratory				Baculovirus	His tag
ckN2	A/chicken/Hong Kong/G9/1997 (N2)	H9N2			NO	NO	F. Krammer Laboratory				Baculovirus	His tag
ckNL H5	A/chicken/Netherlands/14015531/2014 (H5 from novel H5N8)	H5N8	HA	HA0	YES	NO	F. Krammer Laboratory				Baculovirus	His tag
ckNL N8	A/chicken/Netherlands/14015531/2014 (N8 from novel H5N8)	H5N8			NO	NO	F. Krammer Laboratory				Baculovirus	His tag
ddH3	A/canine/NY/120106.2/2011 (H3 equine lineage-dog isolate)	H3	HA	HA0	NO	YES	F. Krammer Laboratory				Baculovirus	His tag
Denv h1	A/Denver/1/1957 (H1)	H1N1	HA	HA0	YES	NO	F. Krammer Laboratory				Baculovirus	His tag
dH3	A/canine/Texas/12/2004 (H3 equine lineage-dog isolate)	H3	HA	HA0	NO	YES	F. Krammer Laboratory				Baculovirus	His tag
DR13	A/DR/7293/2013 (H1)	H1N1	HA	HA0	YES	NO	F. Krammer Laboratory				Baculovirus	His tag
FM1	A/Fort/Monmouth/1/1947 (H1)	H1N1	HA	HA0	YES	NO	F. Krammer Laboratory				Baculovirus	His tag
FM1.1	A/Fort/Monmouth/1/1947 (H1)	H1N1	HA	HA0	YES	NO	F. Krammer Laboratory				Baculovirus	His tag
gfH9 (sfH9)	A/guinea fowl/Hong Kong/WF10/1999 (H9)	H9N2	HA	HA0	YES	NO	F. Krammer Laboratory				Baculovirus	His tag
Guan H7.1	A/Guangdong/17SF003/2016	H7N9	HA	HA0	NO	YES	F. Krammer Laboratory	Yangtse River clade, high path			Baculovirus	His tag
H11	A/shoveler/Netherlands/18/1999 (H11)	H11	HA	HA0	YES	NO	F. Krammer Laboratory				Baculovirus	His tag
H12	A/mallard/Interior Alaska/7MP0167/2007 (H12)	H12	HA	HA0	YES	NO	F. Krammer Laboratory				Baculovirus	His tag
H13	A/black headed gull/Sweden/1/1999 (H13)	H13	HA	HA0	YES	NO	F. Krammer Laboratory				Baculovirus	His tag
H14$_1$0/13/2014	A/mallard/Gurjev/263/1982 (H14)	H14	HA	HA0	NO	YES	F. Krammer Laboratory				Baculovirus	His tag
H14$_2$/1/2017	A/mallard/Gurjev/263/1982 (H14)	H14	HA	HA0	NO	YES	F. Krammer Laboratory				Baculovirus	His tag
H15	A/shearwater/West Australia/2576/1979 (H15)	H15	HA	HA0	NO	YES	F. Krammer Laboratory				Baculovirus	His tag
H16	A/black headed gull/Sweden/5/1999 (H16)	H16	HA	HA0	YES	NO	F. Krammer Laboratory				Baculovirus	His tag

Short ID	Long ID	Subtype	Mol ID1	Mol ID2	Is Group 1	Is Group 2	Source	Form1	Form2	GenBank	Expression System	Notes
H17	A/yellow shouldered bat/Guatemala/06/2010 (H17)	H17N10	HA	HA0	YES	NO	F. Krammer Laboratory				Baculovirus	His tag
H18	A/bat/Peru/33/2010 (H18)	H18N11	HA	HA0	YES	NO	F. Krammer Laboratory				Baculovirus	His tag
H2 Mal	A/mallard/Netherlands/5/1999 (H2)	H2N9	HA	HA0	YES	NO	F. Krammer Laboratory				Baculovirus	His tag
H3v$_0$5/15/2015	A/Indiana/10/2011 (H3v)	H3N2	HA	HA0	NO	YES	F. Krammer Laboratory				Baculovirus	His tag
H3v$_0$9/17/2015	A/Indiana/10/2011 (H3v)	H3N2	HA	HA0	NO	YES	F. Krammer Laboratory				Baculovirus	His tag
H4	A/duck/Czech/1956 (H4)	H4N6	HA	HA0	NO	YES	F. Krammer Laboratory				Baculovirus	His tag
H6	A/mallard/Sweden/81/2002 (H6)	H6	HA	HA0	YES	NO	F. Krammer Laboratory				Baculovirus	His tag
H7 Mallard	A/mallard/Netherlands/12/2000 (H7)	H7N3	HA	HA0	NO	YES	F. Krammer Laboratory				Baculovirus	His tag
H8	A/mallard/Sweden/24/2002 (H8)	H8	HA	HA0	YES	NO	F. Krammer Laboratory				Baculovirus	His tag
H9 head	H9 head-only	H9	HA	HA1	YES	NO	F. Krammer Laboratory				Baculovirus	His tag
HK14 N2	A/HongKong/2014/2017	H7N2			NO	NO	F. Krammer Laboratory	Pearl River clade, low path			Baculovirus	His tag
HK14N2	A/HongKong/2014/2017	H7N2			NO	NO	F. Krammer Laboratory	Pearl River clade, low path			Baculovirus	His tag
HK17 H7	A/HongKong/2014/2017	H7N9	HA	HA0	NO	YES	F. Krammer Laboratory	Pearl River clade, low path			Baculovirus	His tag
HK68 H3$_0$8/21/2017	A/Hong Kong/1/1968 (H3)	H3N2	HA	HA0	NO	YES	F. Krammer Laboratory				Baculovirus	His tag
HK68 H3$_6$/6/2016	A/Hong Kong/1/1968 (H3)	H3N2	HA	HA0	NO	YES	F. Krammer Laboratory				Baculovirus	His tag
Hk68 NA	A/Hong Kong/1/1968 (N2)	H3N2			NO	NO	F. Krammer Laboratory				Baculovirus	His tag
Hunan H7.1	A/Hunan/02285/2017	H7N9	HA	HA0	NO	YES	F. Krammer Laboratory	Yangtse River clade, low path			Baculovirus	His tag
Indo H5.1	A/Indonesia/05/2005 (H5)	H5N1	HA	HA0	YES	NO	F. Krammer Laboratory				Baculovirus	His tag
jal	A/chicken/Jalisco/12283/2012 (H7)	H7N3	HA	HA0	NO	YES	F. Krammer Laboratory				Baculovirus	His tag
Jap57 H2	A/Japan/305/1957 (H2)	H2N2	HA	HA0	YES	NO	F. Krammer Laboratory				Baculovirus	His tag
JDH10	A/Jiangxi/Donghu/346/2013 (H10)	H10N8	HA	HA0	NO	YES	F. Krammer Laboratory				Baculovirus	His tag
Mich15	A/Michigan/45/15 (pdmH1N1)	H1N1	HA	HA0	YES	NO	F. Krammer Laboratory				Baculovirus	His tag
N3	A/swine/Missouri/4296424/2006 (N3)	N3			NO	NO	F. Krammer Laboratory				Baculovirus	His tag
N4	A/mallard/Sweden/24/2002 (N4)	N4			NO	NO	F. Krammer Laboratory				Baculovirus	His tag
N5	A/mallard/Sweden/86/2003 (N5)	H12N5			NO	NO	F. Krammer Laboratory				Baculovirus	His tag
N6	A/mallard/Netherlands/1/1999 (N6)	N6			NO	NO	F. Krammer Laboratory				Baculovirus	His tag
N7	A/mallard/IA/10BM01929/2010 (N7)	H10N7			NO	NO	F. Krammer Laboratory				Baculovirus	His tag
NC99 HA	A/New Caledonia/20/1999 (H1)	H1N1	HA	HA0	YES	NO	F. Krammer Laboratory				Baculovirus	His tag

Short ID	Long ID	Subtype	Mol ID1	Mol ID2	Is Group 1	Is Group 2	Source	Form1	Form2	GenBank	Expression System	Notes
NC99 NA	A/New Caledonia/20/1999 (N1)	H1N1			NO	NO	F. Krammer Laboratory				Baculovirus	His tag
NYC H7	A/feline/New York/16-040082-1/2016	H7N2	HA	HA0	NO	YES	F. Krammer Laboratory				Baculovirus	His tag
Pan99 NA	A/Panama/2007/1999 (N2)	H3N2			NO	NO	F. Krammer Laboratory				Baculovirus	His tag
Panama H3	A/Panama/2007/1999 (H3)	H3N2	HA	HA0	NO	YES	F. Krammer Laboratory				Baculovirus	His tag
Perth H3	A/Perth/16/2009 (H3)	H3N2	HA	HA0	NO	YES	F. Krammer Laboratory				Baculovirus	His tag
Phil82 (H3)	A/Philippines/2/1982 (H3)	H3N2	HA	HA0	NO	YES	F. Krammer Laboratory				Baculovirus	His tag
Phil82 H3	A/Philippines/2/1982 (H3)	H3N2	HA	HA0	NO	YES	F. Krammer Laboratory				Baculovirus	His tag
Pintail H5	A/Northern Pintail/WA/40964/2014 (H5 from novel H5N2)	H5N2	HA	HA0	YES	NO	F. Krammer Laboratory				Baculovirus	His tag
PR8 HA	A/PR/8/1934 (H1)	H1N1	HA	HA0	YES	NO	F. Krammer Laboratory				Baculovirus	His tag
PR8 NA	A/PR/8/1934 (N1)	H1N1			NO	NO	F. Krammer Laboratory				Baculovirus	His tag
Rhea H7	A/rhea/North Carolina/39482/93 (H7)	H7N1	HA	HA0	NO	YES	F. Krammer Laboratory				Baculovirus	His tag
SGN2	A/Singapore/1/1957 (N2)	H2N2			NO	NO	F. Krammer Laboratory				Baculovirus	His tag
SH3	A/harbor seal/Massachusetts/1/2011 (H3)	H3N8	HA	HA0	NO	YES	F. Krammer Laboratory				Baculovirus	His tag
Shenzen H5	A/Shenzen/1/16 (H5 from lethal human H5N6 case)	H5N6	HA	HA0	YES	NO	F. Krammer Laboratory				Baculovirus	His tag
SHT.1	A/Shanghai/1/2013 (H7)	H7N9	HA	HA0	NO	YES	F. Krammer Laboratory				Baculovirus	His tag
Swiss H3	A/Switzerland/9715293/2013	H3N2	HA	HA0	NO	YES	F. Krammer Laboratory				Baculovirus	His tag
Swiss Miss H2	A/swine/Missouri/4296424/2006 (H2)	H2N3	HA	HA0	YES	NO	F. Krammer Laboratory				Baculovirus	His tag
TW H6	A/Taiwan/2/13 (H6)	H6N1	HA	HA0	YES	NO	F. Krammer Laboratory				Baculovirus	His tag
Tx 91 h1	A/Texas/36/1991 (H1)	H1N1	HA	HA0	YES	NO	F. Krammer Laboratory				Baculovirus	His tag
TX12N2	A/Texas/50/2012 (N2)	H9N2			NO	NO	F. Krammer Laboratory				Baculovirus	His tag
USSR	A/USSR/1977 (H1)	H1N1	HA	HA0	YES	NO	F. Krammer Laboratory				Baculovirus	His tag
USSR.1	A/USSR/1977 (H1)	H1N1	HA	HA0	YES	NO	F. Krammer Laboratory				Baculovirus	His tag
Vic H3	A/Victoria/361/2011 (H3)	H3N2	HA	HA0	NO	YES	F. Krammer Laboratory				Baculovirus	His tag
Vic11 H3	A/Victoria/361/2011 (H3)	H3N2	HA	HA0	NO	YES	F. Krammer Laboratory				Baculovirus	His tag
Vn04 H5 (HA).1	A/Vietnam/1204/2004 (H5)	H5N1	HA	HA0	YES	NO	F. Krammer Laboratory				Baculovirus	His tag
Vn04 NA	A/Vietnam/1204/2004 (N1)	H5N1			NO	NO	F. Krammer Laboratory				Baculovirus	HisÂătag
Vn04H5	A/Vietnam/1204/2004 (H5)	H5N1	HA	HA0	YES	NO	F. Krammer Laboratory				Baculovirus	His tag
Wisc H3	A/Wisconsin/67/2005 (H3)	H3N2	HA	HA0	NO	YES	F. Krammer Laboratory				Baculovirus	His tag

Short ID	Long ID	Subtype	Mol ID1	Mol ID2	Is Group 1	Is Group 2	Source	Form1	Form2	GenBank	Expression System	Notes
WSN	A/William Smith Neurotropic/1933 (H1)	H1N1	HA	HA0	YES	NO	F. Krammer Laboratory				Baculovirus	His tag
Wyo H3	A/Wyoming/3/2003 (H3)	H3N2	HA	HA0	NO	YES	F. Krammer Laboratory				Baculovirus	His tag
gyrfalcon/Washington/41088-6/2014 (H5N8).1	A/gyrfalcon/Washington/41088-6/2014 (H5N8)	H5N8	HA	HA0	YES	NO	Vanderbilt University					
