# Supplementary material for: The antibody landscapes following AS03 and MF59 adjuvanted H5N1 vaccination
Source: NPJ Vaccines. 2022 Aug 30;7:103. doi: 10.1038/s41541-022-00524-7 (PMC9427073; doi:10.1038/s41541-022-00524-7)
Supplement: Supplementary file 2 — REPORTING SUMMARY [file 41541_2022_524_MOESM2_ESM.pdf]

nature portfolio

Corresponding author(s): Philip Felgner

Last updated by author(s): Philip Felgner

Reporting Summary

Nature Portfolio wishes to improve the reproducibility of the work that we publish. This form provides structure for consistency and transparency in reporting. For further information on Nature Portfolio policies, see our Editorial Policies and the Editorial Policy Checklist.

Statistics

For all statistical analyses, confirm that the following items are present in the figure legend, table legend, main text, or Methods section.

n/a	Confirmed	
☐	☒	The exact sample size (n) for each experimental group/condition, given as a discrete number and unit of measurement
☐	☒	A statement on whether measurements were taken from distinct samples or whether the same sample was measured repeatedly
☐	☒	The statistical test(s) used AND whether they are one- or two-sided<br>*Only common tests should be described solely by name; describe more complex techniques in the Methods section.*
☐	☒	A description of all covariates tested
☐	☒	A description of any assumptions or corrections, such as tests of normality and adjustment for multiple comparisons
☐	☒	A full description of the statistical parameters including central tendency (e.g. means) or other basic estimates (e.g. regression coefficient) AND variation (e.g. standard deviation) or associated estimates of uncertainty (e.g. confidence intervals)
☐	☒	For null hypothesis testing, the test statistic (e.g. F, t, r) with confidence intervals, effect sizes, degrees of freedom and P value noted<br>*Give P values as exact values whenever suitable.*
☒	☐	For Bayesian analysis, information on the choice of priors and Markov chain Monte Carlo settings
☒	☐	For hierarchical and complex designs, identification of the appropriate level for tests and full reporting of outcomes
☐	☒	Estimates of effect sizes (e.g. Cohen's d, Pearson's r), indicating how they were calculated

Our web collection on statistics for biologists contains articles on many of the points above.

Software and code

Policy information about availability of computer code

Data collection	n/a
Data analysis	n/a

For manuscripts utilizing custom algorithms or software that are central to the research but not yet described in published literature, software must be made available to editors and reviewers. We strongly encourage code deposition in a community repository (e.g. GitHub). See the Nature Portfolio guidelines for submitting code & software for further information.

Data

Policy information about availability of data

All manuscripts must include a data availability statement. This statement should provide the following information, where applicable:

- Accession codes, unique identifiers, or web links for publicly available datasets
- A description of any restrictions on data availability
- For clinical datasets or third party data, please ensure that the statement adheres to our policy

All underlying data will be made available via the supplemental information in the form of CSV files. Additional information about the data files will be made available via the supplemental text that will be included as part of the supplemental information.

Field-specific reporting

Please select the one below that is the best fit for your research. If you are not sure, read the appropriate sections before making your selection.

☒ Life sciences ☐ Behavioural & social sciences ☐ Ecological, evolutionary & environmental sciences

For a reference copy of the document with all sections, see nature.com/documents/nr-reporting-summary-flat.pdf

Life sciences study design

All studies must disclose on these points even when the disclosure is negative.

Sample size	Future use study without a prior defined hypothesis and thus sample size
Data exclusions	Four samples were excluded from the IgG and two samples were excluded from the IgA analysis due to globally outlying profiles in PCA plots.
Replication	The supplemental protein microarray used for the second, third, and fourth probing experiments included 283 non-control antigens including 11 antigens that were spotted on the array more<br>than once and one duplicate pair of antigens that was removed due to low correlation between replicates<br>(gyrfalcon/Washington/41088-6/2014 (H5N8)_batch12)
Randomization	Subjects were randomized to treatment
Blinding	The two parent clinical trials were double blinded (Participant and Investigator)

Reporting for specific materials, systems and methods

We require information from authors about some types of materials, experimental systems and methods used in many studies. Here, indicate whether each material, system or method listed is relevant to your study. If you are not sure if a list item applies to your research, read the appropriate section before selecting a response.

Materials & experimental systems

n/a	Involved in the study	
☒	☐	Antibodies
☒	☐	Eukaryotic cell lines
☒	☐	Palaeontology and archaeology
☒	☐	Animals and other organisms
☐	☒	Human research participants
☐	☒	Clinical data
☒	☐	Dual use research of concern

Methods

n/a	Involved in the study	
☒	☐	ChIP-seq
☒	☐	Flow cytometry
☒	☐	MRI-based neuroimaging

Human research participants

Policy information about studies involving human research participants

Population characteristics	healthy adult male and non-pregnant females age 18 to 49 years
Recruitment	Recruitment was performed in the local community through IRB-approved advertisements
Ethics oversight	The study was reviewed and approved by each site's IRB.

Note that full information on the approval of the study protocol must also be provided in the manuscript.

Clinical data

Policy information about clinical studies

All manuscripts should comply with the ICMJE guidelines for publication of clinical research and a completed CONSORT checklist must be included with all submissions.

Clinical trial registration	NCT01317758 and NCT01317745
Study protocol	The future-use specimens analyzed in this study were collected according to the study protocols as published in the referenced peer-review publications indicated in NCT01317758 and NCT01317745
Data collection	Data were extracted from the completed datasets of the primary studies referenced in NCT01317758 and NCT01317745

Outcomes | Future use analysis of serum collected at Days 1, 21, and 42
